# Twelve New Seco-Pregnane Glycosides from *Cynanchum taihangense*

**DOI:** 10.3390/molecules27175500

**Published:** 2022-08-26

**Authors:** Yu-Bo Wang, Dan Zhao, Shan-Shan Su, Gang Chen, Hai-Feng Wang, Yue-Hu Pei

**Affiliations:** 1 School of Pharmacy, Jinzhou Medical University, Jinzhou 121001, China; 2 School of Traditional Chinese Materia Medica, Shenyang Pharmaceutical University, Shenyang 110016, China; 3 Research & Development Center, Zhejiang Xianju Pharmaceutical Co., Ltd., Taizhou 317300, China; 4 Key Laboratory of Food Safety Research in Qinghai Province, Xining Customs District, Xining 810003, China

**Keywords:** *Cynanchum taihangense*, pregnane steroidal glycosides, cynataihoside I–T, NMR data, cytotoxicity

## Abstract

For our interest in the potential biologically active and structurally unique steroidal glycosides, continued phytochemical investigation of *Cynanchum taihangense* was carried out; twelve new seco-pregnane glycosides, cynataihosides I–L (**1**–**4**), M-T (**7**–**14**), and two known glycosides, glaucoside A (**5**) and atratcynoside F (**6**), were isolated from the 95% ethanol extract of *Cynanchum taihangense*. Two new aglycones were found among compounds **10**, **11**, **13**, and **14**. The structures of the glycosides were elucidated based on 1D and 2D NMR spectroscopic data, HR-ESI-MS analysis, and chemical evidence. The cytotoxicity of compounds against three human tumor cell lines (HL*-*60, THP-1, and PC-3) were evaluated by MTT assay. Compound **11** displayed significant cytotoxicity against THP-1 and PC-3 cell line with IC_50_ values of 5.08 and 22.75 μm, respectively. Compounds **3** and **14** exhibited moderate and selective cytotoxicity on HL*-*60 and THP-1 with IC_50_ values of 17.78 and 16.02 μm, respectively.

## 1. Introduction

**Figure 1:**
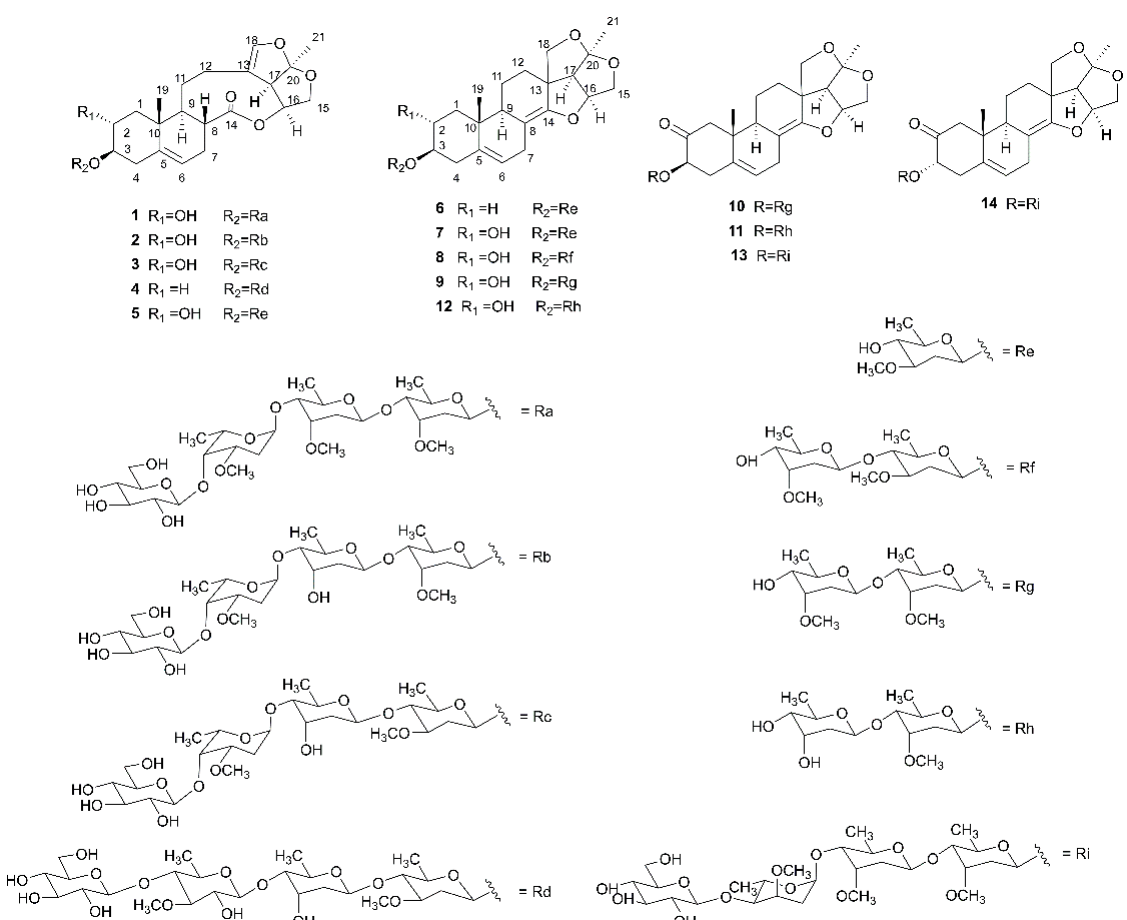
Structures of compounds **1**–**14**.

The chemical structures of C_21_ steroidal glycosides were classified into polyhydroxypregnane-type and seco-pregnae-type glycosides [1,2,3]. Seco-pregnane glycosides, as one higher oxidation degree type of C_21_ steroidal glycosides with extensive biological activities, have been found in Sect. Vincetoxicum of *Cynanchum* which includes many plants used in traditional Chinese medicine [4,5,6,7,8,9]. *Cynanchum taihangense* (“Tai-Hang-Bai-Qian” in Chinese) is a herbaceous plant and is chiefly distributed in Shanxi Province, China [10]. To investigate the potential pregnane glycosides chemical structure of the plant and their activity, we studied the 95% EtOH extract of *Cynanchum taihangense* (*C. taihangense*) previously [11,12,13]. Here, we further report twelve new pregnane glycosides, namely, cynataihosides I–L (**1**–**4**), M–T (**7**–**14**), with totally two new and four known types of aglycones, and two known glycosides, glaucoside A (**5**) and atratcynoside F (**6**), which were obtained from the continued phytochemical research of *C. taihangense*. Their structures are shown in Figure 1. The structural elucidation of new compounds and cytotoxicity of all compounds for human cancer cell lines (HL*-*60, THP-1, and/or PC-3) are described.

## 2. Results and Discussion

The fresh plants of *C. taihangense* were extracted with 95% ethanol. After concentration, the water suspension of the extraction was partitioned with petroleum ether, ethyl acetate, and *n*-butanol, successively. Then, the ethyl acetate and *n*-butanol extract were subjected to various chromatographic isolation methods, respectively, to give twelve new and two known seco-pregnane glycosides. Their structures and the absolute configurations were elucidated by analysis of 1D/2D NMR spectroscopic data, HR-ESI-MS analysis, and chemical evidence (Supplementary Figures S1–S88).

### 2.1. Structure Elucidation

**Figure 2:**
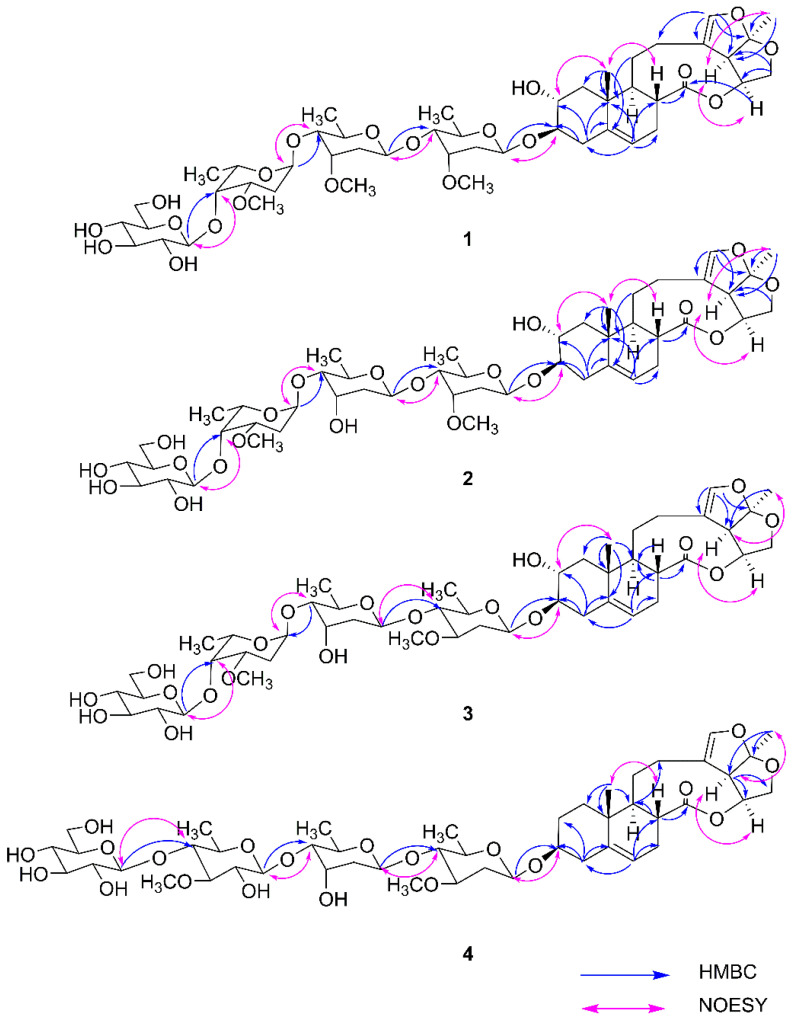
Key HMBC and NOESY correlations of compounds **1**–**4**.

Cynataihoside I (1) was afforded as light yellow amorphous gum, [*α*${]}_{D}^{20}$ −33.18° (*c* 0.75, MeOH). Its positive HR-ESI-MS showed an ion peak at *m/z* 993.4678 [M + Na]^+^ (calculated (calcd) for C_48_H_74_NaO_20_, 993.4671), indicating a molecular formula of C_48_H_74_O_20_. The ^1^H NMR spectrum of **1** displayed characteristic signals of steroidal glycoside with a 13,14:14,15-disecopregnane-type skeleton aglycone: two tertiary methyl groups (*δ*_H_ 0.89 (3H, s, H-19) and 1.53 (3H, s, H-21)), an oxygen-substituted methine proton (*δ*_H_ 5.43 (1H, m), H-16)), one olefinic proton (*δ*_H_ 5.41 (1H, m, H-6)), and one olefinic deshielded proton (6.47 (1H, s, H-18)). The ^1^H and ^13^C NMR signals were assigned by the HSQC spectrum. The HMBC cross-peaks of H-19/C-10, C-1, C-9, and C-5 indicated the connection of rings A and B. Furthermore, correlations of H-21/C-20, C-21, and C-17 were observed. HMBC correlations from *δ*_H_ 5.41 (H-6) to *δ*_C_ 28.2 (C-7), 37.3 (C-4), 39.2 (C-10), and 40.0 (C-8), and from *δ*_H_ 6.47 (H-18) to *δ*_C_ 114.1 (C-13), 23.6 (C-12), 55.9 (C-17), and 118.3 (C-20) proved the presence of the Δ^5–6^ and Δ^13–18^ olefinic groups. Interactions from *δ*_H_ 2.47 (H-8) and 5.43 (H-16) to *δ*_C_ 175.1 (C-14), though ^2^*J*_CH_ and ^3^*J*_CH_, respectively, verified that C-16 of the furan ring and C-8 were connected through an ester carbonyl group (Figure 2). Further HMBC spectrum detailed analysis confirmed the structure of the aglycone (Figure 2). The ^1^H and ^13^C NMR data (Table 1) of the aglycone part of **1** were basically consistent with a known steroidal aglycone, glaucogenin A [14], except for the glycosidation shifts at C-2 (−2.5), C-3 (+8.7), and C-4 (−2.6). In addition, the data were almost the same as those of glaucogenin A in sublanceoside B_2_ [15], which implied that **1** was glaucogenin A linked to a sugar chain at its C-3 hydroxyl group. The ^13^C NMR data of the sugar moiety of **1** were virtually identical to those of cynamooreoside H [16]. Thus, it was assumed that **1** possessed four sugar units and the sequences were *β*-*D*-glucopyranosyl-(1→4)*-α*-*L*-diginopyranosyl-(1→4)-*β*-*D*-cymaropyranosyl-(1→4)-*β*-*D*-cymaropyranosyl. These were supported by spectroscopic analyses and acid hydrolysis. The linkage of the four sugars were determined by HMBC correlations from *δ*_H_ 4.96 (H-1′′′′ of *β*-glucopyranose) to *δ*_C_ 74.1 (C-4′′′ of *α*-diginopyranose), from *δ*_H_ 5.20 (H-1′′′ of *α*-diginopyranose) to *δ*_C_ 82.0 (C-4′′ of *β*-cymaropyranose), from *δ*_H_ 5.05 (H-1′′ of *β*-cymaropyranose) to *δ*_C_ 82.7 (C-4′ of *β*-cymaropyranose), and from *δ*_H_ 5.18 (H-1′ of *β*-cymaropyranose) to *δ*_C_ 85.2 (C-3) (Figure 2). On acid hydrolysis, **1** gave cymarose, diginose, and glucose. In addition, the orientations of the aglycone and sugars were elucidated through the NOESY experiments: H-19/H-8, H-2 (*β*-orientated); H-17/H-16 and H-21 (*α*-orientated); H-1′/H-3, H-5′, and C-3′-OCH_3_ (*α*-orientated); H-1′′/H-5′′ (*α*-orientated); H-3ʹʹʹ/H-5ʹʹʹ (*β*-orientated); H-1ʹʹʹʹ/ H-3ʹʹʹʹ, H-5ʹʹʹʹ (*β*-orientated) (Figure 2). Thus, the structure of **1** was established as glaucogenin A 3-*O*-*β*-*D-*glucopyranosyl-(1→4)*-α*-*L-*diginopyranosyl-(1→4)-*β*-*D-*cymaropyranosyl-(1→4)-*β*-*D-*cymaropyranoside, and named cynataihoside I.

Cynataihoside J (**2**) was isolated as light yellow amorphous gum. Its molecular structure was deduced as C_47_H_72_O_20_ based on a quasi-molecular ion peak at *m/z* 979.4509 (calcd, 979.4515) for [M + Na]^+^ ion in the HR-ESI-MS data. Detailed analysis of ^1^H and ^13^C NMR spectra revealed that the structure of **2** was similar to that of compound **1,** and the difference was only in the second sugar, which was attached to the first cymarose. A comparison of the NMR data of **2** with those of cynataihoside C [11] indicated that they had the same aglycone and two inside deoxysugars, *β*-*D*-digitoxopyranosyl-(1→4)-*β*-*D-*cymaropyranosyl, linked at the C-3 hydroxyl group. Thus, the sugar unit different from compound **1** was supposed to be *β*-*D*-digitoxopyranosyl. Acid hydrolysis of **2** afforded four sugars: cymarose, digitoxose, diginose, and glucose. The sugar sequence and the linkage sites to the aglycone moiety of **2** were demonstrated by the HMBC correlations between H-1′-Cym (*δ*_H_ 5.19) and C-3 (*δ*_C_ 85.2); H-1′′-Dgt (*δ*_H_ 5.32) and C-4′-Cym (*δ*_C_ 82.7); H-1′′′-Dgn (*δ*_H_ 5.27) and C-4′′-Dgt (*δ*_C_ 82.0); H-1′′′′-Glc (*δ*_H_ 4.94) and C-4′′′-Dgn (*δ*_C_ 74.4). In the NOESY spectrum, correlations for H-19 to H-2 (*β*-orientated), for H-17 to H-21 (*α*-orientated), for H-1ʹ to H-5ʹ (*α*-orientated), for H-1ʹʹ to H-5ʹʹ(*α*-orientated), for H-5ʹʹʹ to H-3ʹʹʹ (*β*-orientated), and for H-1ʹʹʹʹ to H-3ʹʹʹʹ and H-5ʹʹʹʹ(*α*-orientated) suggested the relative stereochemistry (Figure 2). Therefore, compound **2** was determined to be glaucogenin A 3-*O*-*β*-*D*-glucopyranosyl-(1→4)*-α*-*L-*diginopyranosyl-(1→4)-*β*-*D*-digitoxopyranosyl-(1→4)-*β*-*D*-cymaropyranoside, and named cynataihoside J.

Cynataihoside K (**3**) was isolated as light yellow amorphous gum. Its molecular formula was determined to be C_47_H_72_O_20_ by HR-ESI-MS (*m/z* 979.4515 [M + Na]^+^, calcd for 979.4515). From its ^1^H and ^13^C NMR spectrum, **3** possessed the similar structure to **2** except for the replacement of the inner sugar. Comparison of ^1^H and ^13^C NMR data of **3** with cynataihoside H [13] showed that **3** had the same aglycone and the first inner sugar as those in cynataihoside H: glaucogenin A and oleandrose. Meanwhile, ^13^C NMR data of *β*-*D*-digitoxopyranosyl connected with *β*-*D*-oleandropyranosyl in cynamooreoside O [16] were essentially in agreement with those of the corresponding part of **3**, indicating that *β*-*D*-oleandropyranosyl was a segment of the sugar chain. By detailed analyses of its 2D (HSQC, HMBC and NOESY) spectra, the structure of **3** was further confirmed. On acid hydrolysis, **3** yielded four corresponding sugars. Thus, the above-mentioned evidences determined the structure of **3** as glaucogenin A 3-*O*-*β*-*D*-glucopyranosyl-(1→4)*-α*-*L*-diginopyranosyl-(1→4)-*β*-*D*-digitoxopyranosyl-(1→4)-*β*-*D*-oleandropyranoside, and named cynataihoside K.

Cynataihoside L (**4**) was obtained as light yellow amorphous gum. The molecular formula was established as C_47_H_72_O_20_ based on the quasi-molecular ion peak at *m/z* 979.4507 (calcd, 979.4515) for [M + Na]^+^ ion observed in its HR-ESI-MS spectrum. The ^1^H NMR of **4** showed the presence of two tertiary methyl groups (*δ*_H_ 0.83 (3H, s, H-19) and 1.54 (3H, s, H-21)), one olefinic proton (*δ*_H_ 5.39 (1H, m, H-6)), one olefinic deshielded proton (*δ*_H_ 6.47 (1H, s, H-18)) connected with the trisubstituted double bond, and two oxygen-substituted methine protons (*δ*_H_ 3.74 (1H, overlapped with other signals, H-3) and 5.43 (1H, m, H-16)). The data and corresponding ^13^C NMR (Table 1) were basically consistent with those of glaucogenin C, a known steroidal aglycone, in cynataihoside E [12] and cynatratoside E [7]. The ^1^H NMR spectrum of **4** showed four anomeric proton signals at (*δ*_H_ 4.79 (1H, br d, *J* = 9.6 Hz), 5.51 (1H, br d, *J* = 9.6 Hz), 5.12 (1H, d, *J* = 7.9 Hz), and 4.74 (1H, d, *J* = 7.8 Hz)), indicating the existence of four *β*-linked sugars. The ^1^H and ^13^C NMR signals were assigned with the help of an extensive study of HMQC, HMBC, and NOESY experiments. The existence of *β*-*D*-oleandropyranosyl, *β*-*D*-digitoxopyranosyl, *β*-*D*-thevetopyranosyl, and *β*-*D-*glucopyranosyl units was confirmed by a further comparison of the sugar chain spectroscopic data of **4** with those of *β*-*D*-oleandropyranosyl, *β*-*D*-digitoxopyranosyl, and *β*-*D-*glucopyranosyl in cynataihoside E [12] and cynatratoside E [7], *β*-*D*-glucopyranosyl-(1→4)-*β*-*D*-thevetopyranosyl in hoodigoside E [17], and *β*-*D*-digitoxopyranosyl in stauntoside L [18]. HMBC correlations further consolidated the sugar sequence: H-1′-Ole (*δ*_H_ 4.79) with C-3 (*δ*_C_ 77.3), H-1′′-Dgt (*δ*_H_ 5.51) with C-4′-Ole (*δ*_C_ 82.9), H-1′′′-The (*δ*_H_ 4.74) with C-4′′-Dgt (*δ*_C_ 83.3), and H-1′′′′-Glc (*δ*_H_ 5.12) with C-4′′′-The (*δ*_C_ 82.6). In the NOESY spectrum, correlations of H-19 to H-8 (*β*-orientated), of H-17 to H-21 (*α*-orientated), of H-1′ to H-3, H-3′, and H-5′ (*α*-orientated), of H-1′′ to H-5′′ (*α*-orientated), of H-1′′′ to 3′′′ and 5′′′ (*α*-orientated), and of H-1′′′′ to H-3′′′′ and 5′′′′ (*α*-orientated) indicated the orientation of the aglycone and sugars (Figure 2). Thus, compound **4** was elucidated to be glaucogenin C 3-*O*-*β*-*D*-glucopyranosyl-(1→4)*-β*-*D*-thevetopyranosyl-(1→4)-*β*-*D*-digitoxopyranosyl-(1→4)-*β*-*D*-oleandropyranoside, and named cynataihoside L.

**Figure 3:**
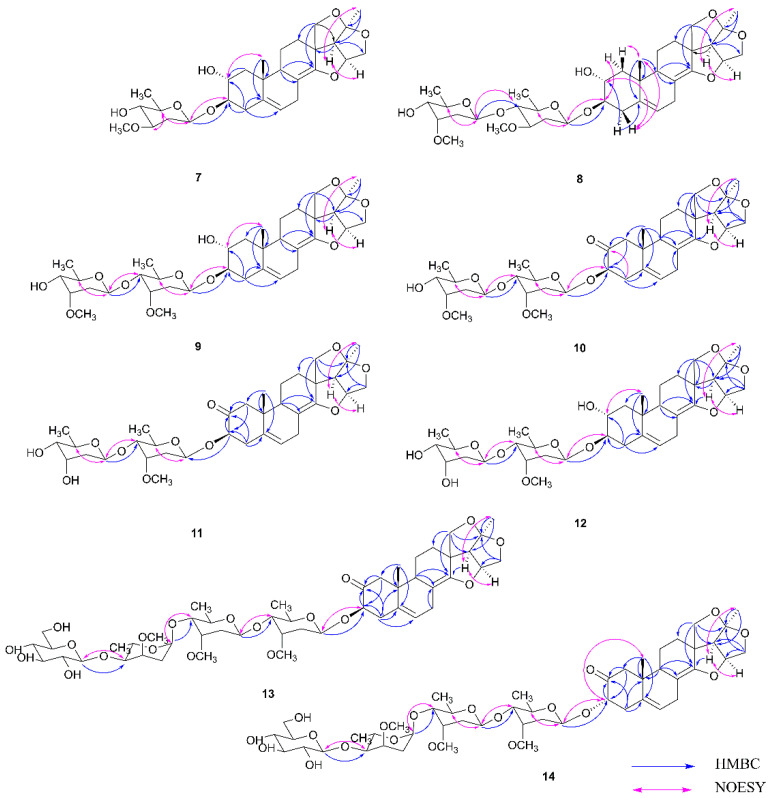
Key HMBC and NOESY correlations of compounds **7**–**14**.

Cynataihoside M (**7**) was isolated as white amorphous gum. The [M + Na]^+^ ion in HR-ESI-MS (*m/z* 527.2572, calcd, 527.2621) indicated that the molecular formula of **7** was C_28_H_40_O_8_. The ^1^H NMR spectrum of **7** showed two tertiary methyl singlets at *δ*_H_ 0.84 (3H, s, H-19) and 1.56 (3H, s, H-21), two oxygen-substituted methine protons at *δ*_H_ 4.25 (1H, br d, *J* = 10.5 Hz, H-15a), 3.78 (1H, dd, *J* = 10.5, 4.3 Hz, H-15b), 4.07 (1H, d, *J* = 8.7 Hz, H-18a), and 4.01 (1H, d, *J* = 8.7 Hz, H-18b), and one olefinic proton at *δ*_H_ 5.45 (1H, m, H-6). In the ^13^C NMR spectrum, four olefinic carbons at *δ*_c_ 140.1 (C-5), 120.2 (C-6), 103.4 (C-8), and 152.8 (C-14) were observed. The NMR data were basically consistent with those of a known steroidal aglycone 2*α*-hydroxyanhydrohirundigenin in sublanceoside E_4_ [15]. Thus, the glycoside consisted of 2*α*-hydroxyanhydrohirundigenin with a sugar chain linked at its C-3 hydroxyl group. The proton signals attributed to one secondary methyl and one methoxyl methyl group of deoxysugar, and one anomeric proton signal at *δ*_H_ 4.83 (1H, dd, *J* = 9.7, 1.5 Hz) indicated that **7** had one deoxysugar with *β*-linkages. The data and its corresponding ^13^C NMR data was identical to that of *β*-*D*-oleandropyranosyl in glaucoside-A [14]. Acid hydrolysis yielded oleandrose. All the ^1^H NMR and ^13^C NMR resonance signals were assigned using HSQC and HMBC spectra. HMBC correlation from *δ*_H_ 4.83 (H-1′) to *δ*_C_ 84.3 (C-3) further revealed the connection site to the aglycone moiety. Relative configuration of **7** was supported by the correlations: H-19/H-2 (*β*-orientated); H-17/H-16 and H-21 (*α*-orientated); H-1′/H-3 and H-5′, and H-5′/ H-3′ (*α*-orientated) (Figure 3). Consequently, the structure of **7** was established as 2*α*-hydroxyanhydrohirundigenin 3-*O*-*β*-*D*-oleandropyranoside, and named cynataihoside M.

Cynataihoside N (**8**) was isolated as white amorphous gum, with a molecular formula of C_35_H_52_O_11_, which was determined by HR-ESI-MS (*m/z* 671.3423 [M + Na]^+^, calcd for 671.3407). The ^1^H and ^13^C NMR spectra of **8** presented similar spectral features to those of **7**. From the spectral data similarities, they shared the same aglycone structure and one of the sugars linked at its C-3 hydroxyl group. The anomeric proton resonances at *δ*_H_ 4.78 (1H, br d, *J* = 8.6 Hz, H-1′) and 5.23 (1H, br d, *J* = 8.7 Hz, H-1′′) implied the existence of two *β*-form sugars. The ^13^C NMR data of the sugar units were basically in accordance with those of *β*-*D*-oleandropyranosyl in cynataihoside H [13] and the methyl *β*-*D*-cymaropyranoside [7]. Acid hydrolysis of **8** gained oleandrose and cymarose. In HMBC spectrum, the key correlations (Figure 3) between 4.78 (H-1′) and *δ*_C_ 84.1 (C-3), 5.23 (H-1′′) and *δ*_C_ 82.4 (C-4′) suggested the sequence of the sugar chain at C-3. The NOE correlations (Figure 3) from *δ*_H_ 0.83 (H-19) to 2.45 (H-4b) (*β*-orientated), from *δ*_H_ 2.45 (H-4b) to 3.98 (H-2) (*β*-orientated), from *δ*_H_ 3.67 (H-3) to 1.41 (H-1b) (*α*-orientated), from *δ*_H_ 2.20 (H-1a) to 0.83 (H-19) (*β*-orientated), from H-17 to H-21 (*α*-orientated), from H-1′ to H-3 and H-5′ (*α*-orientated), and from H-1′′ to H-5′′ (*α*-orientated) revealed the orientations. Thus, compound **8** was characterized as 2*α*-hydroxyanhydrohirundigenin 3-*O*-*β*-*D*-cymaropyranosyl-(1→4)-*β*-*D*-oleandropyranoside, and named cynataihoside N.

Cynataihoside O (**9**) was purified as white amorphous powder, [*α*${]}_{D}^{20}$ −65.00° (*c* 0.20, MeOH). Its molecular formula was established to be C_35_H_52_O_11_ on the basis of HR-ESI-MS at *m*/*z* 671.3374 [M + Na]^+^ (calcd for 671.3407). The ^1^H NMR spectrum of **9** showed the signals corresponding to tertiary methyl groups (*δ*_H_ 0.81 (3H, s, H-19) and 1.56 (3H, s, H-21)) and an olefinic proton (*δ*_H_ 5.37 (1H, m, H-6)). In ^13^C NMR spectrum, 21 carbon signals were ascribed to the aglycone with a seco-pregnane skeleton. The ^1^H and ^13^C NMR data (Table 2) of the aglycone part of **9** were basically in consistency with those of **7**. Thus, the glycoside was also composed of 2*α*-hydroxyanhydrohirundigenin with a sugar chain linked at its C-3 hydroxyl group. For the sugar moiety, two secondary methyl signals (*δ*_H_ 1.34 (3H, d, *J* = 6.1 Hz, H-6′) and *δ*_H_ 1.52 (3H, d, *J* = 6.1 Hz, H-6′′)), two methoxyl methyl signals (*δ*_H_ 3.57 (3H, s, H-3′-OMe) and *δ*_H_ 3.45 (3H, s, H-3′′-OMe)), and two anomeric protons signals (*δ*_H_ 5.18 (1H, br d, *J* = 9.5 Hz, H-1′) and 5.05 (1H, br d, *J* = 9.6 Hz, H-1′′)) in the ^1^H NMR spectrum revealed that **9** had two *β*-form deoxysugars units. Comparing the ^13^C NMR data with those of the first cymarose in sublanceoside G_4_ [15] and the methyl *β*-*D*-cymaropyranoside [7] deduced that compound **9** contained two *β*-*D*-cymaropyranosyl groups. Acid hydrolysis yielded *D*-cymarose. The HMBC correlation from *δ*_H_ 5.18 (H-1′) to *δ*_c_ 84.4 (C-3), and from *δ*_H_ 5.05 (H-1′′) to *δ*_c_ 82.6 (C-4′) indicate the connectivity of the sugars and aglycone. The relative stereochemistry of **9** was elucidated by the NOESY spectrum: correlations for H-19 to H-2 (*β*-orientated), for H-17 to H-21 (*α*-orientated), for H-1ʹ to H-5ʹ (*α*-orientated), and for H-1ʹʹ to H-5ʹʹ(*α*-orientated). Therefore, the structure of **9** was determined to be 2*α*-hydroxyanhydrohirundigenin 3-*O*-*β*-*D*-cymaropyranosyl-(1→4)-*β*-*D*-cymaropyranoside, and named cynataihoside O.

Cynataihoside P (**10**) was obtained as white amorphous powder, with a molecular formula of C_35_H_50_O_11_ by the [M + Na]^+^ ion peak at *m/z* 669.3246 [M + Na]^+^ (calcd for C_35_H_50_NaO_11_ 669.3251) in HR-ESI-MS. The ^1^H NMR data revealed the presence of tertiary methyl groups (*δ*_H_ 0.77 (3H, s, H-19) and *δ*_H_ 1.56 (3H, s, H-21)), one oxygen-substituted methine proton (4.75 (1H, dd, *J* = 12.7, 7.0 Hz, H-3)), four oxygen-substituted methylene protons (4.25 (1H, d, 11.0 Hz, H-15a), 3.78 (1H, dd, *J* = 11.0, 4.3 Hz, H-15b), 4.04 (1H, br d, *J* = 8.7 Hz, H-18a), and 3.98 (1H, d, *J* = 8.7 Hz, H-18b)), and one olefinic proton (*δ*_H_ 5.44 (1H, m, H-6)). In combination with analysis of the HMQC and HMBC spectra, the ^1^H NMR and corresponding ^13^C NMR spectral data (Table 2) of the aglycone moiety of **10** were similar to 2*α*-hydroxyanhydrohirundigenin in **9** except the data due to the A ring and B ring. In the ^13^C NMR spectrum, a characteristic carbon signal at *δ*_C_ 205.7 inferred that the carbon C-2 was carbonylated, which was confirmed by the HMBC correlations from *δ*_H_ 4.75 (H-3), 2.95 (H-4a), 2.37 (H-1a), and 2.33 (H-1b) to *δ*_C_ 205.7, as well as from *δ*_H_ 0.77 (H-19) to *δ*_c_ 137.6 (C-5), 51.5 (C-1), and 44.5 (C-9). Furthermore, correlations between *δ*_H_ 2.34 (H-9) and *δ*_C_ 102.5 (C-8), 153.4 (C-14) established the connectivity between the rings B and C. In addition, the connection of the three furan rings and their connection to the C ring were demonstrated by HMBC correlations of *δ*_H_ 1.56 (H-21) to *δ*_C_ 118.0 (C-20) and 63.3 (C-17), of *δ*_H_ 4.25 (H-15a) to *δ*_C_ 84.3 (C-16), 63.3 (C-17), and 118.0 (C-20), of *δ*_H_ 3.98 (H-18b) to *δ*_C_ 53.6 (C-13), 63.3 (C-17), and 118.0 (C-20), and of *δ*_H_ 4.04 (H-18a) to *δ*_C_ 31.4 (C-12) and 153.4 (C-14). The *α-*orientation of H-3 was suggested by the large coupling constant between H-3 (*δ*_H_ 4.75 (1H, dd, *J* = 12.4, 7.0 Hz)) and H-4 (*δ*_H_ 2.95 (1H, dd, *J* = 14.2, 7.0 Hz, H-4a) and 2.63 (1H, m, H-4b)), as well as the NOE correlations for H-3/H-1b (*δ*_H_ 2.33) and H-4a (*α*-orientated), and for H-19/H-4b (*β*-orientated). In addition, the NOE correlations for H-17/Me-21 indicated their *α-*orientation (Figure 3). Thus, the aglycone structure was determined to be 2-carbonylanhydrohirundigenin. It was a new seco-pregnane-type steroidal aglycone, and named cynataihogenin P. The sugar chain was linked to its C-3 hydroxyl group. The ^1^H NMR displayed two secondary methyl and two methoxyl methyl signals of deoxysugars and two anomeric proton signals at *δ*_H_ 5.28 (1H, dd, *J* = 9.4, 1.7 Hz, H-1′) and 5.06 (1H, dd, *J* = 9.7, 1.6 Hz, H-1′′), which implied the presence of two deoxysugar units with *β*-linkages. The NMR data of sugar moiety were basically consistent with those of **9** except the proton signals due to the anomeric atom connected with the aglycone, which were identical to the data of *β*-*D*-cymarose linked with the aglycone of cynataihoside F [12]. Hydrolysis of **10** gave *D*-cymarose. Thus, compound **10** possessed the same sugar chain as that of **9**. The attachments of the sugars and aglycone were determined by HMBC (Figure 3). The NOE spectrum further suggested the relative stereochemistry of the sugar moiety of **10**: correlations for H-1ʹ to H-5ʹ (*α*-orientated), and for H-1ʹʹ to H-5ʹʹ(*α*-orientated) (Figure 3). Thus, the structure of **10** was established as cynataihogenin P 3-*O*-*β*-*D*-cymaropyranosyl-(1→4)-*β*-*D*-cymaropyranoside, and named cynataihoside P.

Cynataihoside Q (**11**) was white amorphous powder and its positive HR-ESI-MS gave a quasi-molecular ion peak at *m/z* 655.3096 (calcd, 655.3094) for [M + Na]^+^ ion, based on which the molecular formula was calculated as C_34_H_48_O_11_. A detailed comparison between the ^1^H and ^13^C NMR data of **11** and those of **10** in Table 2 indicated they had the same aglycone and one *β*-*D-*cymaropyranosyl connected with their C-3 hydroxyl group, but different terminal sugar. From the ^13^C NMR spectra, the outer deoxysugar units were in good agreement with the terminal *β*-*D-*digitoxopyranosyl in sublanceoside B_2_ [15]. The HMBC spectrum showed correlations from *δ*_H_ 5.35 (1H, br d, *J* = 9.6 Hz, H-1′′) to *δ*_c_ 82.7 (C-4′), and from *δ*_H_ 4.75 (H-3) to *δ*_c_ 95.5 (C-1′), which further confirmed the linkage sugar sequence. On acid hydrolysis of **11**, cymarose and digitoxose were afforded. Thus, all the above-mentioned evidences with the NOESY spectrum in Figure 3 confirmed **11** as cynataihogenin P 3-*O*-*β*-*D-*cymaropyranosyl-(1→4)-*β*-*D-*digitoxopyranoside, and named cynataihoside Q.

Cynataihoside R (**12**) was afforded as white amorphous powder. Its molecular formula was determined to be C_34_H_50_O_11_ by HR-ESI-MS at *m*/*z* 657.3229 [M + Na]^+^ (calcd for 657.3251). From its ^13^C NMR data (Table 2), it was apparent that **12** consisted of the same aglycone as that of **9**. Two anomeric proton signals at *δ*_H_ 5.18 (1H, br d, *J* = 9.6 Hz, H-1′) and 5.34 (1H, br d, *J* = 9.6 Hz, H-1′′) and their corresponding anomeric carbon signals at *δ*_c_ 97.4 and 100.5, respectively, revealed that **12** had two sugars with *β*-linkages. Its NMR data of sugar moiety were a close resemblance to those of **11** except for a small increase of chemical shifts at C-1′ and C-2′ of *β*-*D-*cymaropyranosyl. Considering that the change in chemical shifts was probably caused by different aglycones linked to sugars, it was speculated that they had the same sugar units. Cymaroses and digitoxose were afforded in the acid hydrolysis experiment. In addition, the existence of *β*-*D-*cymaropyranosyl was further confirmed by comparison with the corresponding spectroscopic data of **9**. The linkage positions and sequence of the two sugars were ascertained by HMBC correlations from *δ*_H_ 5.34 (1H, br d, *J* = 9.6 Hz, H-1′′ of *β*-digitoxopyranose) to *δ*_c_ 82.7 (C-4′) and from *δ*_H_ 5.18 (1H, br d, *J* = 9.6 Hz, H-1′ of *β*-cymaropyranose) to *δ*_c_ 84.5 (C-3) (Figure 3). The NOE spectrum further suggested the relative stereochemistry of **12**. Thus, the structure of **12** was elucidated as 2*α*-hydroxyanhydrohirundigenin 3-*O*-*β*-*D-*cymaropyranosyl-(1→4)-*β*-*D-*digitoxopyranoside, and named cynataihoside R.

Cynataihoside S (**13**) was isolated as white amorphous powder. HR-ESI-MS of **13** showed a [M + Na]^+^ ion peak at *m/z* 975.46006 (calcd, 975.456001) that accounted for a molecular formula of C_48_H_72_O_19_. Inspection of the NMR data of **13** in Table 2 revealed the same aglycone as that of **10**. The large coupling constant between H-3 (*δ*_H_ 4.74 (1H, dd, *J* = 12.4, 7.0 Hz)) and H-4 (*δ*_H_ 2.93 (1H, dd, *J* = 14.2, 7.0 Hz, H-4a) and 2.62 (1H, m, H-4b)), as well as the NOE correlation for H-3/H-1b (*δ*_H_ 2.33) and H-4a (*α*-orientated), and for H-19/H-4b (*β*-orientated) confirmed the *α-*orientation of H-3. Thus, **13** was composed of cynataihogenin P and a sugar chain with linked C-3 group. The ^1^H NMR spectrum of **13** showed three secondary methyl and three methoxyl methyl signals of deoxysugars and four anomeric proton signals at *δ*_H_ 5.26 (1H, dd, *J* = 9.6, 1.6 Hz, H-1′), 5.05 (1H, br d, *J* = 9.4 Hz, H-1′′), 4.93 (1H, br d, *J* = 2.5 Hz, H-1′′′), and 4.99 (1H, d, *J* = 7.8 Hz), which revealed the existence of four sugars with three *β*- and one *α*- form. According to the comparison of its ^13^C NMR data to those in cynataihoside F [12], the sugar chain was proposed to be glucopyranosyl-(1→4)-*α*-*L-*cymaropyranosyl-(1→4)-*β*-*D-*cymaropyranosyl-(1→4)-*β*-*D-*cymaropyranosyl. This was further supported by hydrolysis experiment and 2D NMR (Figure 3). Acidic hydrolysis of **13** produced cymarose and glucose. The absolute configurations of sugars were determined by HPLC analysis and the comparison of their spectral data with those that possessed the same sugar chain unit reported in the literature [15]. The connection and the sequence of the sugar chain were further established by HMBC correlations between *δ*_H_ 4.99 (glc-H-1′′′′) and *δ*_C_ 78.7 (*α*-cym-C-4′′′), *δ*_H_ 4.93 (*α*-cym-H-1′′′) and *δ*_C_ 82.0 (*β*-cym-C-4′′), *δ*_H_ 5.05 (*β*-cym-H-1′′) and *δ*_C_ 82.6 (*β*-cym-C-4′), and *δ*_H_ 5.26 (*β*-cym-H-1′) and *δ*_C_ 77.9 (C-3). Therefore, the structure of **13** was identified as cynataihogenin P 3-*O*-*β*-*D-*glucopyranosyl-(1→4)-*α*-*L-*cymaropyranosyl-(1→4)-*β*-*D-*cymaropyranosyl-(1→4)-*β*-*D-*cymaropyranoside, and designated cynataihoside S.

Cynataihoside T (**14**) was obtained as white amorphous powder, and possessed the same molecular formula as **13** according to HR-ESI-MS data (*m/z* 975.45963 [M + Na]^+^, calculated for C_48_H_72_NaO_19_, 975.456001). The ^1^H and ^13^C NMR signals assignable to **14** unambiguously by HSQC and HMBC analyses were extremely similar to **13**. The data (Table 2) revealed that they had basically consistent sugar units, but differed a little bit in the aglycone moiety. The ^1^H NMR signal due to H-3 of **14** was observed at *δ*_H_ 4.50 (1H, t, *J* = 3.5 Hz) instead of *δ*_H_ 4.74 (1H, dd, *J* = 12.4, 7.0 Hz, H-3 of **13**), indicating the *β*-orientation of H-3 in **14**. The absence of the NOESY correlations of *δ*_H_ 4.50 (H-3) to *δ*_H_ 2.82 (H-4a) and of *δ*_H_ 2.82 (H-4a) to *δ*_H_ 0.80 (H-19) also supported the *β*-orientation of H-3. In addition, other signals by detailed 1D NMR and 2D NMR spectral analysis were also assigned to elucidate the structure (Figure 3). Therefore, the aglycone of **14** was determined as 3-*epi*-cynataihogenin P, also a new compound, named cynataihogenin T. Acid hydrolysis of **14** yielded cymarose and glucose. Hence, together with the 2D NMR (Figure 3) analyses, the structure of compound **14** was elucidated to be cynataihogenin T 3-*O*-*β*-*D-*glucopyranosyl-(1→4)-*α*-*L-*cymaropyranosyl-(1→4)-*β*-*D-*cymaropyranosyl-(1→4)-*β*-*D-*cymaropyranoside, and designated cynataihoside T.

The known compounds were identified as glaucoside A [14] (**5**) and atratcynoside F [6] (**6**) by comparing their ^1^H and ^13^C NMR data to those in the literature.

### 2.2. Cytotoxic Activities

The cytotoxic activities of compounds **1**–**14** against HL*-*60 (human leukemic promyelocytic cell), THP-1 (human acute monocytic leukemia cell line) and compounds **1**, **3**, **10**–**14** against PC-3 (prostate cancer cell line) were evaluated. The cytotoxicity data represented by IC_50_ values in μm are shown in Table 3, in which 5-Fluorouracil was used as a positive control and their IC_50_ values against HL*-*60, THP-1, and PC-3 were 9.93, 5.82, and 22.15 μm, respectively. As evident from results, the compounds exhibited varying degrees of cytotoxic activity. Compound **11** was more active than others, especially on THP-1 and PC-3, and showed significant cytotoxicity similar to the positive control. Compounds **3** and **14** moderately and selectively inhibited the proliferation of HL*-*60 and THP-1 with IC_50_ values of 17.78 and 16.02 μm, respectively.

## 3. Materials and Methods

### 3.1. General Experiment Procedure

Optical rotations were measured on a WZZ-2A (Shanghai base solid Instrument Co., Ltd., Shanghai, China). The IR spectra were obtained from a Bruker IFS-55 spectrophotometer (Karlsruhe, Germany) with KBr disks. HR-ESI-MS data were measured on a Micro-mass Autospec-UntimaE TOF mass spectrophotometer (Waters, Milford, MA, USA) and a Bruker Solarix 7.0T FT-ICR MS system (Bruker, Germany). NMR spectra were run on a Bruker AVANCE-400/-600 spectrometer (Karlsruhe, Germany). Analytical HPLC was performed on a Shimadzu LC-10AT (Kyoto, Japan) liquid chromatograph and preparative HPLC separation was carried out on a YMC-Pack ODS-A column (10 × 250 mm, 5 μm; YMC-Pack, Kyoto, Japan), equipped with a Shimadzu LC-8A pump (Kyoto, Japan) and a Shimadzu SP*D-*10A UV–V is detector (Kyoto, Japan). Sugars analytical HPLC was carried out on a Jasco PU-4180 pump (Kyoto, Japan) and an OR-4090 detector (Kyoto, Japan).

### 3.2. Plant Material

The fresh whole plants (4.7 kg) of *C. taihangense* were collected in August 2014 at Wangmang Ridge in Shanxi Province, China. A voucher specimen was identified by Prof. Jing-Ming Jia of Shenyang Pharmaceutical University and was deposited in the School of Traditional Chinese Materia Medica of Shenyang Pharmaceutical University (NO. SYPC201408316).

### 3.3. Cell Lines

The human leukemic promyelocytic cell (HL*-*60), human acute monocytic leukemia cell line (THP-1), and prostate cancer cell line (PC-3) were provided by America Type Culture Collection, ATCC (Rockville, MD, USA).

### 3.4. Extraction and Isolation

The fresh whole plants (4.7 kg) of *C. taihangense* were extracted with 95% EtOH, and water suspension of the extraction was then partitioned with petroleum ether, ethyl acetate, and *n*-butanol, successively [11].

The ethyl acetate extract (110 g) was further pre-fractioned by a gel column to give thirteen fractions (Fr. 1–13). Fr. 6 was separated by the same procedures as those in our previous report [11] to afford nine fractions (Fr. 6-5-4-4-1–Fr. 6-5-4-4-9). Fr. 6-5-4-4-4 was purified via Sephadex LH-20 eluting with MeOH, and then isolated by semi-preparative HPLC eluting with CH_3_OH/H_2_O (67:33 *v*/*v*, flowrate 2.5 mL/min) to yield compound **1** (20 mg). Fr. 8 was separated into five fractions (Fr. 8-1–Fr. 8-5) by a C18 column using gradients of MeOH/H_2_O (20:80) to (100:0) as the eluent. Fr. 8-4 was further submitted to a silica gel column with CH_2_Cl_2_/MeOH (100:0 to 0:100 *v*/*v*) to obtain sub-fractions. Fr. 8-4-5 and Fr. 8-4-6 were sent to a reversed-phase preparative HPLC in CH_3_CN/H_2_O (45:55 *v*/*v*, flowrate 4 mL/min), respectively. Compound **4** (7 mg) was obtained from Fr. 8-4-6. Compound **3** (18 mg) was obtained from Fr. 8-4-5. Fr. 8-4-5-4 was further purified by preparative TLC to give compound **2** (7 mg).

The *n*-butanol extract (150 g) was further fractionated by D101 macroporous adsorptive resins column chromatography eluting with MeOH/H2O (0:100–100:0, *v/v*) to obtain six fractions (Fr. 1–6). Fr. 6 was separated into fifteen fractions (Fr. 6-1–Fr. 6-15) by silica gel column using CH2Cl2/MeOH (100:0–0:100, *v/v*) as the eluent. Fr. 6-2, Fr. 6-3, Fr. 6-4, Fr. 6-7, and Fr. 6-8 were further subjected to semi-preparative HPLC with the elution of MeOH/H2O (57:43 *v/v*, flowrate 2.5 mL/min) and CH3CN/H2O (48:52 *v/v*, 48:52 *v/v*, 48:52 *v/v*, flowrate 4 mL/min, and 42:58 *v/v*, flowrate 3 mL/min), respectively. Compound **6** (9 mg, t_R_ 36 min) and compound **8** (10 mg, t_R_ 43 min) were separately obtained from Fr. 6-2 and Fr. 6-4. Compounds 5 (9 mg, t_R_ 29 min), 9 (11 mg, t_R_ 53 min), and **10** (45 mg, t_R_ 32 min) were recrystallized from Fr. 6-3. Compound 11 (5 mg, t_R_ 19 min) and **12** (9 mg, t_R_ 28 min) were obtained from Fr. 6-7. Compound 13 (60 mg, t_R_ 110 min) and 14 (24 mg, t_R_ 140 min) were afforded from Fr. 6-8.

Cynataihoside I (**1**): Light yellow amorphous gum (MeOH), [*α*${]}_{D}^{20}$ −33.18° (*c* 0.75, MeOH); IR (KBr) *ν*_max_ cm^−1^: 3440, 2970, 2934, 2170, 1737, 1653, 1632, 1450, 1384, 1311, 1273, 1165, 1083, 1023, 914, 864; for ^1^H NMR and ^13^C NMR (C_5_D_5_N) data see Table 1; HR-ESI-MS *m/z*: 993.4678 [M + Na]^+^ (calcd for C_48_H_74_NaO_20_, 993.4671).

Cynataihoside J (**2**): Light yellow amorphous gum (MeOH), [*α*${]}_{D}^{20}$ −245.00° (*c* 0.10, MeOH); IR (KBr) *ν*_max_ cm^−1^: 3429, 2933, 2170, 1736, 1631, 1384, 1311, 1272, 1165, 1081, 1021, 914, 865, 832; for ^1^H NMR and ^13^C NMR (C_5_D_5_N) data see Table 1; HR-ESI-MS *m/z*: 979.4509 [M + Na]^+^ (calcd for C_47_H_72_NaO_20_ 979.4515).

Cynataihoside K (**3**): Light yellow amorphous gum (MeOH), [*α*${]}_{D}^{20}$ −37.98° (*c* 1.47, MeOH); IR (KBr) *ν*_max_ cm^−1^: 3436, 2933, 2170, 1736, 1631, 1384, 1310, 1272, 1164, 1102, 1079, 1020, 903, 876, 833, 809; for ^1^H NMR and ^13^C NMR (C_5_D_5_N) data see Table 1 HR-ESI-MS *m/z*: 979.4515 [M + Na]^+^ (calcd for C_47_H_72_NaO_20_, 979.4515).

Cynataihoside L (**4**): Light yellow amorphous gum (MeOH), [*α*${]}_{D}^{20}$*−*9.00° (*c* 0.20, MeOH); IR (KBr) *ν*_max_ cm^−1^: 3423, 2933, 2170, 1736, 1653, 1631, 1406, 1384, 1310, 1272, 1164, 1080, 913, 872, 832; for ^1^H NMR and ^13^C NMR (C_5_D_5_N) data see Table 1; HR-ESI-MS *m/z*: 979.4507 [M + Na]^+^ (calcd for C_47_H_72_NaO_20_, 979.4515).

Cynataihoside M (**7**): White amorphous gum (MeOH), [*α*${]}_{D}^{20}$ −80.33° (*c* 0.10, MeOH); IR (KBr) *ν*_max_ cm^−1^: 3429, 2934, 2170, 1631, 1407, 1384, 1272, 1166, 1103, 1066, 987, 896, 833; for ^1^H NMR and ^13^C NMR (C_5_D_5_N) data see Table 1; HR-ESI-MS *m/z*: 527.2572 [M + Na]^+^ (calcd for C_28_H_40_NaO_8_, 527.2621).

Cynataihoside N (**8**): White amorphous gum (MeOH), [*α*${]}_{D}^{20}$ −105.50° (*c* 0.20, MeOH); IR (KBr) *ν*_max_ cm^−1^: 3439, 2934, 2659, 2170, 1631, 1406, 1384, 1272, 1163, 1062, 1007, 916, 833; for ^1^H NMR and ^13^C NMR (C_5_D_5_N) data see Table 1; HR-ESI-MS *m/z*: 671.3423 [M + Na]^+^ (calcd for C_35_H_52_NaO_11_, 671.3407).

Cynataihoside O (**9**): White amorphous powder, [*α*${]}_{D}^{20}$ −65.00° (*c* 0.20, MeOH); for ^1^H NMR and ^13^C NMR (C_5_D_5_N) data see Table 2; HR-ESI-MS *m/z*: 671.3374 [M + Na]^+^ (calcd for C_35_H_52_NaO_11_, 671.3407).

Cynataihoside P (**10**): White amorphous powder, [*α*${]}_{D}^{20}$ −51.13° (*c* 0.40, MeOH); IR (KBr) *ν*_max_ cm^−1^: 3420, 2967, 2933, 2170, 1735, 1631, 1407, 1384, 1273, 1192, 1163, 1146, 1089, 1066, 1004, 920, 900, 868, 832; for ^1^H NMR and ^13^C NMR (C_5_D_5_N) data see Table 2; HR-ESI-MS *m/z*: 669.3246 [M + Na]^+^ (calcd for C_35_H_50_Na O_11_, 669.3251).

Cynataihoside Q (**11**): White amorphous powder, [*α*${]}_{D}^{20}$ −251.00° (*c* 0.10, MeOH); IR (KBr) *ν*_max_ cm^−1^: 3430, 2932, 2170, 1726, 1632, 1406, 1384, 1388, 1271, 1165, 1064, 1009, 867, 833; for ^1^H NMR and ^13^C NMR (C_5_D_5_N) data see Table 2; HR-ESI-MS *m/z*: 655.3096 [M + Na]^+^ (calcd for C_34_H_48_NaO_11_, 655.3094).

Cynataihoside R (**12**): White amorphous powder, [*α*${]}_{D}^{20}$ −120.17° (*c* 0.20, MeOH); IR (KBr) *ν*_max_ cm^−1^: 3430, 2933, 2659, 2170, 1631, 1405, 1384, 1319, 1271, 1165, 1161, 1010, 916, 867, 833; for ^1^H NMR and ^13^C NMR (C_5_D_5_N) data see Table 2; HR-ESI-MS *m/z*: 657.3229 [M + Na]^+^ (calcd for C_34_H_50_NaO_11_, 657.3251).

Cynataihoside S (**13**): White amorphous powder, [*α*${]}_{D}^{20}$ −193.53° (*c* 0.17, MeOH); IR (KBr) *ν*_max_ cm^−1^: 3444, 2971, 2934, 1728, 1632, 1451, 1383, 1319, 1195, 1061, 1004, 896, 867, 835; for ^1^H NMR and ^13^C NMR (C_5_D_5_N) data see Table 2; HR-ESI-MS *m/z*: 975.46006 [M + Na]^+^ (calcd for C_48_H_72_NaO_19_, 975.456001).

Cynataihoside T (**14**): White amorphous powder, [*α*${]}_{D}^{20}$ −57.92° (*c* 0.53, MeOH); IR (KBr) *ν*_max_ cm^−1^: 3439, 2970, 2933, 2170, 1722, 1632, 1384, 1319, 1273, 1195, 1165, 1061, 1006, 896, 868, 834; for ^1^H NMR and ^13^C NMR (C_5_D_5_N) data see Table 2; HR-ESI-MS *m/z*: 975.45963 [M + Na]^+^ (calcd for C_48_H_72_NaO_19_, 975.456001).

### 3.5. Acid Hydrolysis of Compounds ***1***–***4*, *7***–***8***, and ***9***–***14***

Acid hydrolysis was prepared by following the methods described in our previous report [11,12,13]. Each solution of **7** (15 mg), **8**, **9**, **11**, **12** (2 mg), and **10** (30 mg), in MeOH (1 mL for **8**, **9**, **11**, and **12**, and 3 mL for **7** and **10**) were heated separately with 0.1 N H_2_SO_4_ (1 mL for **8**, **9**, **11**, and **12**, and 3 mL for **7** and **10**) at 50 °C for 30 min. Then the mixture was diluted with water (2 mL for **8**, **9**, **11**, and **12**, and 6 mL for **7** and **10**) and concentrated to (2 mL for **8**, **9**, **11**, and **12**, and 6 mL for **7** and **10**). After that, the solution was kept at 60 °C for a further 30 min, followed by neutralizing with aqueous saturated Ba(OH)_2_, and the precipitates were filtered off.

Each solution of **1** (5 mg), **2** (2 mg), **3** (5 mg), **4** (2 mg), **13** (18 mg), and **14** (18 mg) in 50% 1,4-dioxane (4 mL, 2 mL, 4 mL, 2 mL, 10 mL, and 10 mL) was heated separately with 0.5 N H_2_SO_4_ (4 mL, 2 mL, 4 mL, 2 mL, 10 mL, and 10 mL) at 95 °C for 3 h. Reaction mixture was cooled, neutralized with aqueous saturated Ba(OH)_2_, and the precipitates were filtered off [17].

The filtrate was partitioned between CH_2_Cl_2_ and H_2_O. The aqueous layer was concentrated and further analyzed by TLC. The known steroidal glycosides were hydrolyzed to give deoxysugars to make a comparison. Three solvent systems, CHCl_3_/CH_3_OH (9:1 *v*/*v*), CH_2_Cl_2_/C_2_H_5_OH (9:1 *v*/*v*), and PE/acetone (3:2 *v*/*v*), were performed to reference the R_f_ values of oleandrose, digitoxose, cymarose, and diginose according to the literature [8]. In the hydrolysate of **1**–**3** and **7**–**14**, the corresponding deoxysugars R_f_ values were basically identical to the corresponding ones mentioned in the reports.

### 3.6. Determination of Absolute Configuration of Sugars

The configurations of the monosaccharides were identified by the comparison of their spectral data with those in the literature and HPLC analysis.

The concentrated aqueous layer of **1**–**4**, **8**, and **9**–**14** (24 h after dissolution) were subjected to HPLC analysis under the following conditions: column, Shodex Asahipak NH2P-50 4E column (4.6 mm × 250 mm, 5 μm); flowrate, 0.8 mL/min; solvent, MeCN/H_2_O (3:1 *v*/*v*); detection, OR (Jasco OR-4090) detector. *D-*glucose (t_R_ 10.8 min, positive polarity) in compounds **1**–**4**, **13,** and **14**, *D-*digitoxose in compounds **1**–**3**, **11,** and **12**, *L-*diginose in compounds **1**–**3**, and *D-*cymarose in **1**–**2** and **8**–**14** were identified.

Since the impurities produced during the hydrolysis process interfered with the detection, the absolute configuration of oleandrose in compounds **3**, **4**, **7,** and **8** was determined to be *D-*form by the spectroscopic data. Those oleandroses were all directly linked to their respective aglycones so that the absolute configurations could be suggested by the NMR data.

Unfortunately, owing to the failure of hydrolysis of compound **4**, as well as the deficiency of authentic samples of thevetose, the monosaccharides of **4** were not detected completely. The configurations of the deoxysugars were further identified by the comparison of those spectral data with those in the literature.

### 3.7. Cytotoxicity Assay

An MTT assay was used to determine the cytotoxicity effect of the compounds on three cultured human cancer cell lines, including HL*-*60 (human leukemic promyelocytic cell), THP-1 (human acute monocytic leukemia cell line), and PC-3 (prostate cancer cell line). Cell growth inhibition assay was performed as reported previously [19]. 5-Fluorouracil was used as a positive control.

## 4. Conclusions

Fourteen seco-pregnane steroidal glycosides, including twelve new ones (**1**–**4** and **7**–**14**), were isolated from the ethanolic extract of *C. taihangense* by multiple separation methods. All compounds were reported for the first time from the plant. Among them, compounds **10**, **11**, **13,** and **14** contained two new seco-pregnane-type aglycones. In addition, the cytotoxicity of the glycosides against HL*-*60, THP-1, and PC-3 cell lines were evaluated. Compound **11** displayed significant cytotoxicity against THP-1 and PC-3 cell line. Compounds **3** and **14** exhibited moderate and selective cytotoxic activity on HL*-*60 and THP-1.

## Figures and Tables

**Table 1:** ^1^H and ^13^C NMR data in C_5_D_5_N for compounds **1**–**5**, **7**–**8**.

NO.	1		2		3		4		7		8	
	*δ*_H_ ^b^	*δ*_C_ ^d^	*δ*_H_ ^b^	*δ*_C_ ^e^	*δ*_H_ ^b^	*δ*_C_ ^d^	*δ*_H_ ^b^	*δ*_C_ ^d^	*δ*_H_ ^c^	*δ*_C_ ^e^	*δ*_H_ ^c^	*δ*_C_ ^e^
1	2.46 ^a^, 1.23 (m)	44.5	2.46 ^a^,1.23 ^a^	44.5	2.46 ^a^, 1.25 ^a^	44.5	1.81 (m), 0.94 (m)	36.2	2.22 ^a^, 1.42 ^a^	44.6	2.20 ^a^, 1.41 ^a^	44.6
2	4.00 ^a^	69.7	4.00 (m)	69.8	4.00 ^a^	69.6	2.04 ^a^, 1.42 ^a^	29.8	3.99 ^a^	69.9	3.98 (m)	69.8
3	3.58 ^a^	85.2	3.57 ^a^	85.2	3.63 (m)	84.7	3.74 ^a^	77.3	3.70 ^a^	84.3	3.67 (m)	84.1
4	2.48 ^a^, 2.42 ^a^	37.3	2.47 ^a^, 2.41 ^a^	37.3	2.55 ^a^, 2.44 ^a^	37.1	2.55 ^a^, 2.29 (m)	38.8	2.64 (dd, 14.6, 5.0), 2.50 ^a^	37.2	2.61 (dd, 14.2, 5.0), 2.45 ^a^	37.2
5	−	139.5	−	139.6	−	139.5	−	140.4	−	140.1	−	140.1
6	5.41 (m)	120.5	5.40 (m)	120.5	5.44 (m)	120.6	5.39 (m)	120.2	5.45 (m)	120.2	5.42 (m)	120.3
7	2.64 (m), 2.14 ^a^	28.2	2.65 (m), 2.14 (m)	28.2	2.65 (m), 2.14 ^a^	28.2	2.64 ^a^, 2.15 (m)	28.2	3.17 (dd, 21.0, 3.8) 2.74 (d, 21.0)	25.6	3.17 (dt, 21.1, 3.9), 2.74 (d, 21.0)	25.6
8	2.47 ^a^	40.0	2.47 ^a^	40.0	2.49 ^a^	40.0	2.50 ^a^	40.4	−	103.4	−	103.5
9	1.33 ^a^	52.8	1.32 ^a^	52.8	1.32 ^a^	52.8	1.22 (t, 10.3)	53.0	2.20 ^a^	44.8	2.20 ^a^	44.9
10	−	39.2	−	39.4	−	39.3	−	38.4	−	38.5	−	38.5
11	2.10 ^a^, 1.40 ^a^	29.8	2.09 ^a^, 1.39 ^a^	29.8	2.14 ^a^, 1.41 ^a^	29.8	2.12 ^a^, 1.71 (m)	29.7	1.64 ^a^, 1.29 ^a^	20.0	1.63 ^a^ 1.27 ^a^	20.0
12	2.53 (m), 1.33 ^a^	23.6	2.54 ^a^, 1.33 ^a^	23.6	2.55 ^a^, 1.35 ^a^	23.6	2.60 ^a^, 1.38 ^a^	23.7	1.92 (m), 1.41 ^a^	31.6	1.92 (m), 1.41 ^a^	31.6
13	−	114.1	−	114.1	−	114.1	−	114.1	−	53.5	−	53.5
14	−	175.1	−	175.1	−	175.1	−	175.3	−	152.8	−	152.9
15	4.25 ^a^, 3.94 ^a^	67.5	4.23 ^a^, 3.93 (t)	67.6	4.24 (m), 3.93 (m)	67.5	4.24 ^a^, 3.95 ^a^	67.5	4.25 (br d, 10.5), 3.78 (dd, 10.5, 4.3)	72.1	4.25 (br d, 10.8), 3.79 (dd, 10.8, 4.3)	72.1
16	5.43 (m)	75.3	5.43 (m)	75.3	5.43 (m)	75.3	5.44 (m)	75.3	4.76 (dd, 8.0, 4.3)	84.1	4.76 (m)	84.2
17	3.52 ^a^	55.9	3.53 ^a^	55.9	3.53 ^a^	55.9	3.55 ^a^	55.9	2.80 (d, 8.0)	63.3	2.80 (d, 7.9)	63.4
18	6.47 (1H, s)	143.6	6.46 (1H, s)	143.6	6.47 (s)	143.6	6.47 (s)	143.6	4.07 (d, 8.7), 4.01 (d, 8.7)	76.4	4.07 ^a^, 4.01 (d, 8.7)	76.4
19	0.89 (3H, s)	18.7	0.88 (3H, s)	18.7	0.90 (3H, s)	18.7	0.83 (3H, s)	17.6	0.84 (3H, s)	19.4	0.83 (3H, s)	19.4
20	−	118.3	−	118.3	−	118.3	−	118.3	−	118.0	−	118.0
21	1.53 (3H, s)	24.5	1.53 (s)	24.6	1.53 (3H, s)	24.5	1.54 (3H, s)	24.5	1.56 (3H, s)	22.3	1.56 (3H, s)	22.4
	*β*-*D*-Cym		*β*-*D*-Cym		*β*-*D*-Ole		*β*-*D*-Ole		*β*-*D*-Ole		*β*-*D*-Ole	
1′	5.18 (br d, 9.5)	97.6	5.19 (br d, 9.7)	97.6	4.78 (br d, 9.7)	98.7	4.79 (br d, 9.6)	97.9	4.83 (dd, 9.7, 1.5)	98.9	4.78 (br d, 8.6)	98.6
2′	2.35 ^a^,1.89 (m)	36.7	2.35 ^a^,1.90 ^a^	36.8	2.44 ^a^, 1.77 (m)	37.5	2.42 ^a^, 1.81 (m)	37.8	2.50 ^a^, 1.75 (m)	37.0	2.44 ^a^, 1.76 ^a^	37.4
3′	4.04 (m)	77.6	4.10 (m)	77.7	3.56 ^a^	78.7	3.55 ^a^	78.9	3.48 ^a^	81.2	3.55 ^a^	78.7
4′	3.47 ^a^	82.7	3.47 ^a^	82.7	3.54 ^a^	82.4	3.54 ^a^	82.9	3.48 ^a^	75.8	3.51 ^a^	82.4
5′	4.23 ^a^	69.1	4.21 ^a^	69.2	3.56 ^a^	71.7	3.53 ^a^	71.5	3.63 ^a^	72.8	3.59 ^a^	71.7
6′	1.33 (3H, d, 6.2)	17.9	1.29 (3H, d, 6.3)	17.9	1.38 (3H, d, 5.8)	18.2	1.45 (3H, d, 5.3)	18.6	1.53 (3H, d, 6.0)	18.2	1.42 (3H, d, 6.0)	18.3
3′-OMe	3.59 (3H, s)	58.7	5.19 (br d, 9.7)	58.8	3.54 (3H, s)	57.3	3.53 (3H, s)	57.2	3.45 (3H, s)	56.8	3.52 (3H, s)	57.1
	*β*-*D*-Cym		*β*-*D*-Dgt		*β*-*D*-Dgt		*β*-*D*-Dgt				*β*-*D*-Cym	
1′′	5.05 (br d, 9.6)	99.9	5.32 (brd, 9.6)	100.3	5.50 (br d, 9.6)	98.2	5.51 (br d, 9.6)	98.3	−	−	5.23 (br d, 8.7)	98.3
2′′	2.38 ^a^, 1.71 ^a^	34.8	2.40 ^a^, 1.94 ^a^	39.3	2.39 ^a^, 1.94 (m)	39.5	2.42 ^a^, 2.00 ^a^	39.0	−	−	2.36 (m), 1.75(m)	35.6
3′′	3.88 (m)	77.2	4.54 ^a^	67.5	4.56 ^a^	67.6	4.66 ^a^	67.7	−	−	3.75 (m)	78.7
4′′	3.44 ^a^	82.0	3.50 ^a^	82.0	3.51 ^a^	82.0	3.53 ^a^	83.3	−	−	3.52 (m)	73.9
5′′	4.19 ^a^	69.0	4.34 (m)	68.5	4.36 ^a^	68.7	4.34 (m)	68.8	−	−	4.12 (m)	71.0
6′′	1.33 (3H, d, 6.2)	18.3	1.39 (3H, d, 6.3)	18.3	1.41 (3H, d, 6.2)	18.3	1.59 (3H, d, 6.2)	18.3	−	−	1.53 (3H, d, 6.2)	18.8
3′′-OMe	3.51 (3H, s)	57.0	−	−	−	−	−	−	−	−	3.44 (3H, s)	57.7
	*α*-*L*-Dgn		*α*-*L*-Dgn		*α*-*L*-Dgn		*β*-*D*-The					
1′′′	5.20 (br s)	100.8	5.27 (brd, 3.0)	100.6	5.27 (br d, 3.1)	100.5	4.74 (d, 7.8)	105.4	−	−	−	−
2′′′	2.36 ^a^, 2.05 ^a^	31.9	2.37 ^a^, 2.08 ^a^	32.0	2.34 ^a^, 2.06 ^a^	31.9	3.77 ^a^	74.2	−	−	−	−
3′′′	3.88 ^a^	74.9	3.81 (m)	74.8	3.79 (m)	74.7	3.67 (m)	85.6	−	−	−	−
4′′′	4.27 ^a^	74.1	4.19 ^a^	74.4	4.18 ^a^	74.3	3.71 ^a^	82.6	−	−	−	−
5′′′	4.24 ^a^,	67.8	4.38 (m)	67.8	4.38 ^a^	67.8	3.74 ^a^	71.6	−	−	−	−
6′′′	1.68 (3H, d, 6.6)	17.6	1.63 (3H, d, 6.6)	17.5	1.64 (3H, d, 6.6)	17.5	1.68 (3H, d, 6.0)	18.4	−	−	−	−
3′′′-OMe	3.51 (3H, s)	55.5	3.42 (3H, s)	55.3	3.41 (3H, s)	55.3	3.91 (3H, s)	60.3	−	−	−	−
	*β*-*D*-Glc		*β*-*D*-Glc		*β*-*D*-Glc		*β*-*D*-Glc					
1′′′′	4.96 (d, 7.8)	105.1	4.94 (d, 7.8)	105.2	4.93 (br d, 8.0)	105.2	5.12 (d, 7.9)	104.5	−	−	−	−
2′′′′	4.00 ^a^	75.0	4.00 (m)	75.1	3.99 ^a^	75.0	4.01 (dd, 8.2, 7.8)	75.6	−	−	−	−
3′′′′	4.23 ^a^	78.3	4.20 ^a^	78.3	4.19 ^a^	78.3	4.21 ^a^	78.4	−	−	−	−
4′′′′	4.19 ^a^	71.6	4.17 ^a^	71.6	4.18 ^a^	71.5	4.19 ^a^	71.6	−	−	−	−
5′′′′	3.94 ^a^	78.0	3.89 (m)	78.0	3.88 ^a^	77.9	3.95 (m)	78.0	−	−	−	−
6′′′′	4.52 (dd, 11.4, 2.5), 4.34 (dd, 11.4, 5.5)	62.8	4.48 (dd, 11.4, 2.4), 4.33 ^a^	62.8	4.48 (dd, 11.5, 2.4), 4.32 (dd, 11.5, 5.5)	62.7	4.54 (dd, 11.5, 2.5), 4.34 ^a^	62.8	−	−	−	−

^a^ Overlapped with other signals. ^b^ 600 MHz, ^c^ 400 MHz, ^d^ 150 MHz, ^e^ 100 MHz. *J* values in Hz. Cym = cymaropyranose, Dgt = digitoxopyranose, Dgn = diginopyranose, The = thevetopyranose, Ole = oleandropyranose, Glc = glucopyranose.

**Table 2:** ^1^H and ^13^C NMR data in C_5_D_5_N for compounds **9**–**14**.

NO.	9		10		11		12		13		14	
	*δ*_H_ ^b^	*δ*_C_ ^d^	*δ*_H_ ^b^	*δ*_C_ ^d^	*δ*_H_ ^b^	*δ*_C_ ^d^	*δ*_H_ ^b^	*δ*_C_ ^d^	*δ*_H_ ^b^	*δ*_C_ ^e^	*δ*_H_ ^b^	*δ*_C_ ^e^
1	2.18 ^a^, 1.38 ^a^	44.5	2.37 ^a^, 2.33 ^a^	51.5	2.37 ^a^, 2.33 ^a^	51.5	2.18 ^a^, 1.37 ^a^	44.6	2.36 ^a^, 2.33 ^a^	51.1	2.89 (d, 12.7), 2.25 ^a^	47.8
2	3.94 (m)	69.9	−	205.7	−	205.6	3.95 (m)	69.9	−	205.7	−	210.6
3	3.60 ^a^	84.4	4.75 (dd, 12.7, 7.0)	78.0	4.75 ^a^	77.9	3.60 ^a^	84.5	4.74 (dd, 12.4, 7.0)	77.9	4.50 (t, 3.5)	78.7
4	2.52 (dd, 14.0, 4.7), 2.43 (d, 14.0)	37.2	2.95 (dd, 14.2, 7.0), 2.63 (m)	39.4	2.95 (dd, 13.9, 7.0), 2.62 (m)	39.4	2.52 (dd, 14.0, 5.0), 2.41 ^a^	37.3	2.93 (dd, 14.2, 7.0), 2.62 (m)	39.4	2.82 (m), 2.78 ^a^	39.3
5	−	139.9	−	137.6	−	137.6	−	140.0	−	137.6	−	136.9
6	5.37 (m)	120.0	5.44 (m)	122.0	5.45 (m)	122.0	5.36 (m)	120.1	5.45 (m)	122.0	5.41 (m)	122.0
7	3.14 (dt 21.2, 3.9), 2.72 (d, 21.2)	25.5	3.15 (dt, 21.3, 3.8), 2.75 (br d, 21.3)	25.6	3.14 (dt, 21.0, 3.8), 2.74 (br d, 21.0)	25.6	3.14 (br d, 21.0), 2.72 (br d, 21.0)	25.6	3.14 (dt, 21.0, 4.0), 2.75 (br d, 21.0)	25.6	3.10 (dt, 21.0, 3.4) 2.70 (br d, 21.0)	25.5
8	−	103.4	−	102.5	−	102.5	−	103.5	−	102.5	5.28 ^a^	103.0
9	2.17 ^a^	44.7	2.34 ^a^	44.5	2.34 ^a^	44.5	2.17 ^a^	44.8	2.34 ^a^	44.5	2.44 (dd, 8.8, 8.1)	44.4
10	−	38.3	−	43.2	−	43.2	−	38.4	−	43.2	−	42.9
11	1.64 (m), 1.31 ^a^	19.9	1.51 ^a^, 1.22 ^a^	19.8	1.53 ^a^, 1.20 ^a^	19.8	1.64 ^a^, 1.27 ^a^	20.0	1.52 (m), 1.22 (m)	19.8	1.52 (m), 1.22 (m)	19.9
12	1.91 ^a^, 1.40 ^a^	31.5	1.92 ^a^, 1.42 (m)	31.4	1.92 ^a^, 1.42 (m)	31.4	1.90 ^a^, 1.39 ^a^	31.6	1.92 ^a^, 1.42 ^a^	31.4	1.93 ^a^, 1.42 ^a^	31.4
13	−	53.4	−	53.6	−	53.5	−	53.5	−	53.5	−	53.6
14	−	152.7	−	153.4	−	153.3	−	152.8	−	153.3	−	153.2
15	4.24 ^a^, 3.79 (dd, 10.9, 4.4)	72.0	4.25 (d, 11.0), 3.78 (dd, 11.0, 4.3)	72.1	4.25 ^a^, 3.79 (dd, 10.9, 4.3)	72.1	4.24 ^a^, 3.78 (dd, 10.7, 3.9)	72.1	4.24 ^a^, 3.80 (dd, 10.9, 4.3)	72.1	4.23 ^a^, 3.78 (dd, 10.8, 4.3)	72.1
16	4.76 (m)	84.0	4.78 ^a^	84.3	4.76 ^a^	84.2	4.75 (m)	84.1	4.78 (m)	84.3	4.72 (m)	84.3
17	2.81 (d, 8.0)	63.2	2.82 (d,7.8)	63.3	2.82 (d, 7.8)	63.2	2.79 (d, 7.7)	63.3	2.82 (d, 8.0)	63.2	2.78 (d, 7.9)	63.2
18	4.07 ^a^, 4.00 (d, 8.8)	76.3	4.04 (br d, 8.7), 3.98 (d, 8.7)	76.4	4.04 (br d, 8.8), 3.98 (d, 8.8)	76.3	4.07 (d, 8.8), 4.01 (d, 8.8)	76.4	4.03 ^a^, 4.00 ^a^	76.3	4.04 ^a^, 3.98 ^a^	76.4
19	0.81 (3H, s)	19.3	0.77 (3H, s)	19.3	0.77 (3H, s)	19.3	0.81 (3H, s)	19.4	0.76 (3H, s)	19.3	0.80 (3H, s)	19.5
20	−	117.9	−	118.0	−	118.0	−	118.0	−	118.0	−	118.0
21	1.56 (3H, s)	22.2	1.56 (3H, s)	22.3	1.58 (3H, s)	22.3	1.57 (3H, s)	22.3	1.56 (3H, s)	22.3	1.56 (3H, s)	22.3
	*β*-*D*-Cym		*β*-*D*-Cym		*β*-*D*-Cym		*β*-*D*-Cym		*β*-*D*-Cym		*β*-*D*-Cym	
1′	5.18 (br d, 9.5)	97.3	5.28 (1H, dd, 9.4, 1.7)	95.6	5.29 (br d, 9.6)	95.5	5.18 (br d, 9.6)	97.4	5.26 (dd, 9.6, 1.6)	95.5	5.18 (dd, 9.6, 1.7)	97.2
2′	2.32 ^a^, 1.87 ^a^	36.5	2.38 ^a^, 1.95 ^a^	36.2	2.37 ^a^, 1.96 ^a^	36.2	2.33 (m), 1.87 ^a^	36.7	2.35 ^a^, 1.93 ^a^	36.2	2.36 ^a^, 1.88 ^a^	36.5
3′	4.06 ^a^	77.5	4.07 ^a^	77.5	4.10 (m)	77.5	4.09 ^a^	77.6	4.02 ^a^	77.5	4.02 ^a^	77.4
4′	3.46 ^a^	82.6	3.51 ^a^	82.7	3.53 ^a^	82.7	3.50 (m)	82.7	3.45 ^a^	82.6	3.44 ^a^	82.8
5′	4.22 ^a^	69.1	4.21 (m)	69.1	4.23 ^a^	69.1	4.23 ^a^	69.2	4.16 ^a^	69.0	4.12 ^a^	69.2
6′	1.34 (3H, d, 6.1)	17.9	1.35 (3H, d, 6.2)	18.2	1.34 (3H, d, 6.3)	18.2	1.32 (3H, d, 6.4)	18.0	1.31 (3H, d, 6.4)	18.1	1.31 (3H, d, 6.2)	18.1
3′-OMe	3.57 (3H, s)	58.4	3.56 (3H, s)	58.4	3.58 (3H, s)	58.4	3.60 (3H, s)	58.6	3.56 (3H, s)	58.5	3.54 (3H, s)	58.7
	*β*-*D*-Cym		*β*-*D*-Cym		*β*-*D*-Dgt		*β*-*D*-Dgt		*β*-*D*-Cym		*β*-*D*-Cym	
1′′	5.05 (br d, 9.6)	100.1	5.06 (1H, dd, 9.7, 1.6)	100.2	5.35 (br d, 9.6)	100.4	5.34 (br d, 9.6)	100.5	5.05 (br d, 9.4)	100.0	5.03 (br d, 9.4)	100.1
2′′	2.34 ^a^, 1.76 (m)	35.5	2.36 ^a^, 1.77 (m)	35.6	2.42 ^a^, 1.98 ^a^	39.4	2.41 ^a^, 1.98 (m)	39.3	2.27 ^a^, 1.73 (m)	36.7	2.25 ^a^, 1.72 (m)	36.7
3′′	3.73 (m)	78.4	3.73 (d, 2.8)	78.6	4.41 (m)	68.3	4.42 (m)	68.3	3.85 (m)	77.4	3.84 (m)	77.4
4′′	3.51 ^a^	73.7	3.51 ^a^	73.9	3.57 ^a^	73.7	3.57 ^a^	73.7	3.40 ^a^	82.0	3.38 ^a^	81.9
5′′	4.09 ^a^	70.6	4.09 ^a^	70.7	4.26 ^a^	70.1	4.27 ^a^	70.1	4.14 ^a^	69.0	4.11 ^a^	68.9
6′′	1.52 (3H, d, 6.1)	18.6	1.52 (3H, d, 6.3)	18.7	1.57 (3H, d, 6.4)	18.7	1.58 (3H, d, 6.4)	18.7	1.32 (3H, d, 6.4)	18.2	1.28 (3H, d, 6.3)	18.2
3′′-OMe	3.45 (3H, s)	57.7	3.44 (3H, s)	57.9	−	−	−	−	3.56 (3H, s)	58.3	3.55 (3H, s)	58.2
									*α*-*L*-Cym		*α*-*L*-Cym	
1′′′	−	−	−	−	−	−	−	−	4.93 (br d, 2.5)	98.6	4.92 (br d, 2.6)	98.6
2′′′	−	−	−	−	−	−	−	−	2.36 ^a^, 1.79 (m)	32.0	2.35 ^a^, 1.78 (m)	32.0
3′′′	−	−	−	−	−	−	−	−	3.92 ^a^	73.1	3.93 ^a^	73.1
4′′′	−	−	−	−	−	−	−	−	3.95 ^a^	78.7	3.96 ^a^	78.7
5′′′	−	−	−	−	−	−	−	−	4.68 (m)	64.9	4.67 (m)	64.9
6′′′	−	−	−	−	−	−	−	−	1.46 (3H, d, 6.4)	18.2	1.46 (3H, d, 6.4)	18.3
3′′′-OMe	−	−	−	−	−	−	−	−	3.43 (3H, s)	56.5	3.42 (3H, s)	56.6
									*β*-*D*-Glc		*β*-*D*-Glc	
1′′′′	−	−	−	−	−	−	−	−	4.99 (d, 7.8)	102.0	4.99 (d, 7.8)	102.0
2′′′′	−	−	−	−	−	−	−	−	3.96 ^a^	75.0	3.97 ^a^	75.0
3′′′′	−	−	−	−	−	−	−	−	4.22 ^a^	78.2	4.22 ^a^	78.2
4′′′′	−	−	−	−	−	−	−	−	4.19 ^a^	71.5	4.20 ^a^	71.6
5′′′′	−	−	−	−	−	−	−	−	3.97 ^a^	78.4	3.97 ^a^	78.4
6′′′′	−	−	−	−	−	−	−	−	4.56 (dd, 11.0, 2.0), 4.37 (dd, 11.0, 5.5)	62.7	4.56 (dd, 11.0, 2.0), 4.37 (dd, 11.0, 5.5)	62.7

^a^ Overlapped with other signals. ^b^ 600 MHz, ^d^ 150 MHz, ^e^ 100 MHz. *J* values in Hz. Cym = cymaropyranose, Dgt = digitoxopyranose, Glc = glucopyranose.

**Table 3:** Cytotoxicity data of compounds **1**–**14** ^a^.

Compound	HL*-*60	THP1	PC-3
**1**	74.08	57.41	50.85
**2**	54.14	>80	—
**3**	17.78	43.16	52.16
**4**	>80	61.61	—
**5**	54.03	22.95	—
**6**	60.66	>80	—
**7**	>80	64.43	—
**8**	52.31	58.03	—
**9**	59.70	>80	—
**10**	>80	50.98	>80
**11**	24.24	5.08	22.75
**12**	27.98	36.90	>80
**13**	56.72	32.74	75.28
**14**	50.49	16.02	>80
5-Fluorouracil	9.93	5.82	22.15

^a^ Data expressed as IC_50_ values (μm). HL*-*60, human leukemic promyelocytic cell; THP-1, human acute monocytic leukemia cell line; PC-3, prostate cancer cell line.

## Data Availability

The data presented in this study are available in this article.

## Supplementary Materials

The following supporting information can be downloaded at: https://www.mdpi.com/article/10.3390/molecules27175500/s1. **Figures S1–S84**: IR, HR-ESI-MS, 1D, and 2D NMR spectra of compounds **1**–**4**, **7**–**14**; **Figures S85–S88**: 1D NMR spectra of compounds **5** and **6**.

## Sample Availability

Samples of the compounds are not available from the authors.
