# Supplementary material for: Twelve New Seco-Pregnane Glycosides from Cynanchum taihangense
Source: Molecules. 2022 Aug 26;27(17):5500. doi: 10.3390/molecules27175500 (PMC9457764; doi:10.3390/molecules27175500)

Twelve New Seco-Pregnane Glycosides from *Cynanchum taihangense*

Yu-Bo Wang [1,2,*,†], Dan Zhao [3], Shan-Shan Su [4], Gang Chen [2], Hai-Feng Wang [2] and Yue-Hu Pei [2,*]

[1] School of Pharmacy, Jinzhou Medical University, Jinzhou 121001, China
[2] School of Traditional Chinese Materia Medica, Shenyang Pharmaceutical University, Shenyang 110016, China
[3] Research & Development Center, Zhejiang Xianju Pharmaceutical Co., Ltd., Taizhou 317300, China
[4] Key Laboratory of Food Safety Research in Qinghai Province, Xining Customs District, Xining 810003, China
* Correspondence: wangyb@jzmu.edu.cn (Y.-B.W.); peiyueh@vip.163.com (Y.-H.P.)
† This author is the first corresponding author and Jinzhou Medical University is the first corresponding affiliation.

Contents

1. The spectra of compound 1 ······ 7

Figure S1. The IR spectrum of compound **1** ······ 7

Figure S2. The HR-ESI-MS spectrum of compound **1** ······ 7

Figure S3. The ^{1}H-NMR spectrum of compound **1** ······ 8

Figure S4. The ^{13}C-NMR spectrum of compound **1** ······ 8

Figure S5. The HSQC spectrum of compound **1** ······ 9

Figure S6. The HMBC spectrum of compound **1** ······ 9

Figure S7. The partial amplified HMBC spectrum of compound **1** ······ 10

Figure S8. The NOESY spectrum of compound **1** ······ 10

2. The spectra of compound 2 ······ 11

Figure S9. The IR spectrum of compound **2** ······ 11

Figure S10. The HR-ESI-MS spectrum of compound **2** ······ 11

Figure S11. The ^{1}H-NMR spectrum of compound **2** ······ 12

Figure S12. The ^{13}C-NMR spectrum of compound **2** ······ 12

Figure S13. The HSQC spectrum of compound **2** ······ 13

Figure S14. The HMBC spectrum of compound **2** ······ 13

Figure S15. The NOESY spectrum of compound **2** ······ 14

3. The spectra of compound 3 ······ 14

Figure S16. The IR spectrum of compound **3** ······ 14

Figure S17. The HR-ESI-MS spectrum of compound **3** ······ 15

Figure S18. The ^{1}H-NMR spectrum of compound **3** ······ 15

Figure S19. The ^{13}C-NMR spectrum of compound **3** ······ 16

Figure S20. The HSQC spectrum of compound **3** ······ 16

Figure S21. The HMBC spectrum of compound **3** ······ 17

Figure S22. The NOESY spectrum of compound **3** ········ 17

4. The spectra of compound 4 ········ 18

Figure S23. The IR spectrum of compound **4** ········ 18

Figure S24. The HR-ESI-MS spectrum of compound **4** ········ 18

Figure S25. The ^{1}H-NMR spectrum of compound **4** ········ 19

Figure S26. The ^{13}C-NMR spectrum of compound **4** ········ 19

Figure S27. The HSQC spectrum of compound **4** ········ 20

Figure S28. The HMBC spectrum of compound **4** ········ 20

Figure S29. The NOESY spectrum of compound **4** ········ 21

5. The spectra of compound 7 ········ 21

Figure S30. The IR spectrum of compound **7** ········ 21

Figure S31. The HR-ESI-MS spectrum of compound **7** ········ 22

Figure S32. The ^{1}H-NMR spectrum of compound **7** ········ 22

Figure S33. The ^{13}C-NMR spectrum of compound **7** ········ 23

Figure S34. The HSQC spectrum of compound **7** ········ 23

Figure S35. The HMBC spectrum of compound **7** ········ 24

Figure S36. The NOESY spectrum of compound **7** ········ 24

6. The spectra of compound 8 ········ 25

Figure S37. The IR spectrum of compound **8** ········ 25

Figure S38. The HR-ESI-MS spectrum of compound **8** ········ 25

Figure S39. The ^{1}H-NMR spectrum of compound **8** ········ 26

Figure S40. The ^{13}C-NMR spectrum of compound **8** ········ 26

Figure S41. The HSQC spectrum of compound **8** ········ 27

Figure S42. The HMBC spectrum of compound **8** ········ 27

Figure S43. The NOESY spectrum of compound **8** ········ 28

7. The spectra of compound 9 ········ 28

Figure S44. The HR-ESI-MS spectrum of compound **9** ········ 28

Figure S45. The ^{1}H-NMR spectrum of compound **9** ········ 29

Figure S46. The ^{13}C-NMR spectrum of compound **9** ········ 29

Figure S47. The HSQC spectrum of compound **9** ········ 30

Figure S48. The HMBC spectrum of compound **9** ········ 30

Figure S49. The NOESY spectrum of compound **9** ········ 31

8. The spectra of compound 10 ········ 31

Figure S50. The IR spectrum of compound **10** ········ 31

Figure S51. The HR-ESI-MS spectrum of compound **10** ········ 32

Figure S52. The ^{1}H-NMR spectrum of compound **10** ········ 32

Figure S53. The ^{13}C-NMR spectrum of compound **10** ········ 33

Figure S54. The HSQC spectrum of compound **10** ········ 33

Figure S55. The HMBC spectrum of compound **10** ········ 34

Figure S56. The NOESY spectrum of compound **10** ········ 34

9. The spectra of compound 11 ········ 35

Figure S57. The IR spectrum of compound **11** ········ 35

Figure S58. The HR-ESI-MS spectrum of compound **11** ········ 35

Figure S59. The ^{1}H-NMR spectrum of compound **11** ········ 36

Figure S60. The ^{13}C-NMR spectrum of compound **11** ········ 36

Figure S61. The HSQC spectrum of compound **11** ········ 37

Figure S62. The HMBC spectrum of compound **11** ········ 37

Figure S63. The NOESY spectrum of compound **11** ········ 38

10. The spectra of compound 12 ········ 38

Figure S64. The IR spectrum of compound **12** ········ 38

Figure S65. The HR-ESI-MS spectrum of compound **12** ······························ 39

Figure S66. The ^{1}H-NMR spectrum of compound **12** ································· 39

Figure S67. The ^{13}C-NMR spectrum of compound **12** ································ 40

Figure S68. The HSQC spectrum of compound **12** ···································· 40

Figure S69. The HMBC spectrum of compound **12** ··································· 41

Figure S70. The NOESY spectrum of compound **12** ································· 41

11. The spectra of compound 13 ·· 42

Figure S71. The IR spectrum of compound **13** ·· 42

Figure S72. The HR-ESI-MS spectrum of compound **13** ······························ 42

Figure S73. The ^{1}H-NMR spectrum of compound **13** ································· 43

Figure S74. The ^{13}C-NMR spectrum of compound **13** ································ 43

Figure S75. The HSQC spectrum of compound **13** ···································· 44

Figure S76. The HMBC spectrum of compound **13** ··································· 44

Figure S77. The NOESY spectrum of compound **13** ································· 45

12. The spectra of compound 14 ·· 45

Figure S78. The IR spectrum of compound **14** ·· 45

Figure S79. The HR-ESI-MS spectrum of compound **14** ······························ 46

Figure S80. The ^{1}H-NMR spectrum of compound **14** ································· 46

Figure S81. The ^{13}C-NMR spectrum of compound **14** ································ 47

Figure S82. The HSQC spectrum of compound **14** ···································· 47

Figure S83. The HMBC spectrum of compound **14** ··································· 48

Figure S84. The NOESY spectrum of compound **14** ································· 48

13. The spectra of compound 5 ··· 49

Figure S85. The ^{1}H-NMR spectrum of compound **5**···································· 49

Figure S86. The ^{13}C-NMR spectrum of compound **5**··································· 49

14. The spectra of compound 6 ········ 50

Figure S87. The ^{1}H-NMR spectrum of compound **6** ········ 50

Figure S88. The ^{13}C-NMR spectrum of compound **6** ········ 50

1. The spectra of compound 1

Figure S1. The IR spectrum of compound **1**

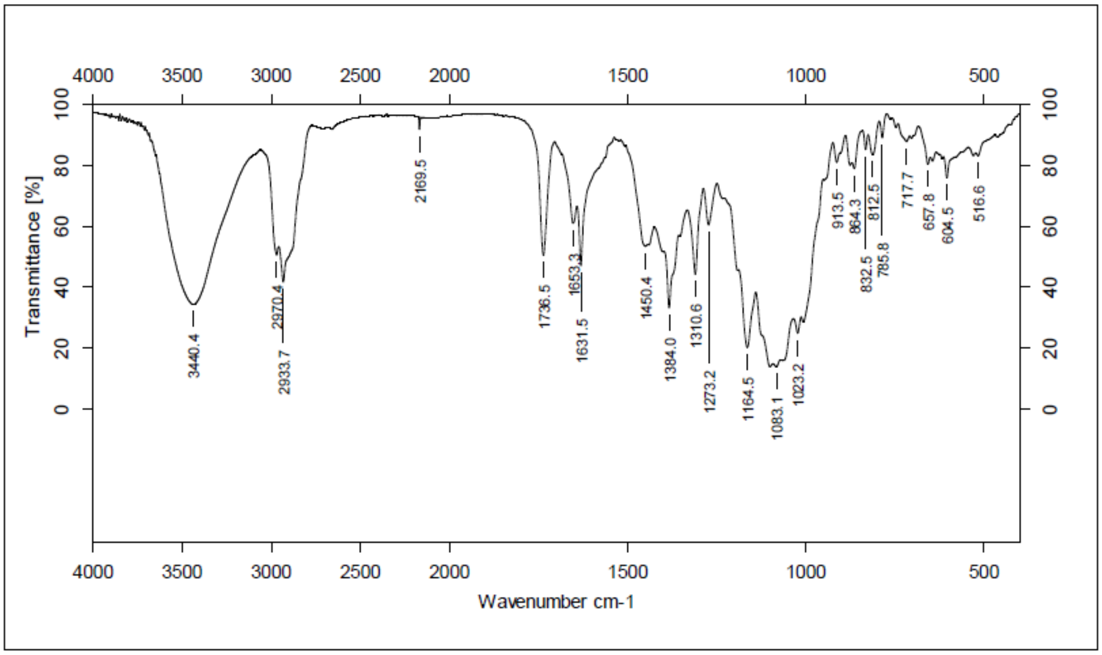

Figure S2. The HR-ESI-MS spectrum of compound **1**

Acquisition Parameter

Source Type	ESI	Ion Polarity	Positive	Set Nebulizer	1.2 Bar
Focus	Active	Set Capillary	4500 V	Set Dry Heater	180 °C
Scan Begin	50 m/z	Set End Plate Offset	-500 V	Set Dry Gas	8.0 l/min
Scan End	1500 m/z	Set Collision Cell RF	400.0 Vpp	Set Divert Valve	Source

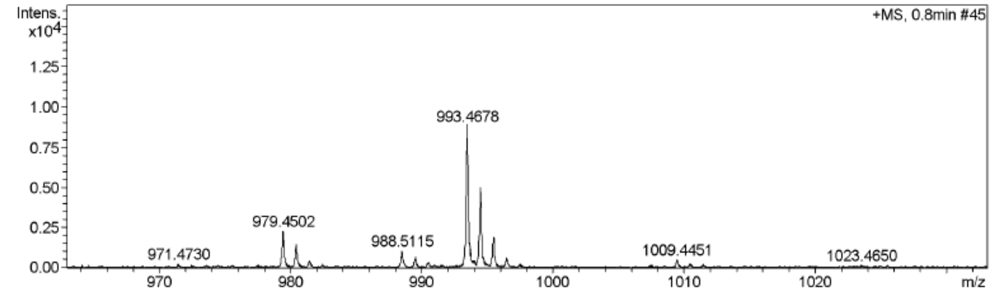

Meas. m/z	#	Formula	m/z	err [ppm]	Mean err [ppm]	rdb	N-Rule	e⁻ Conf	mSigma	Std I	Std Mean m/z	Std I VarNorm	Std m/z Diff	Std Comb Dev
993.4678														
	1	C 48 H 74 Na O 20	993.4671	-0.7	1.6	11.5	ok	even	29.85	0.0443	0.0044	0.0146	0.0078	0.8427

Figure S3. The ^{1}H-NMR spectrum of compound **1**

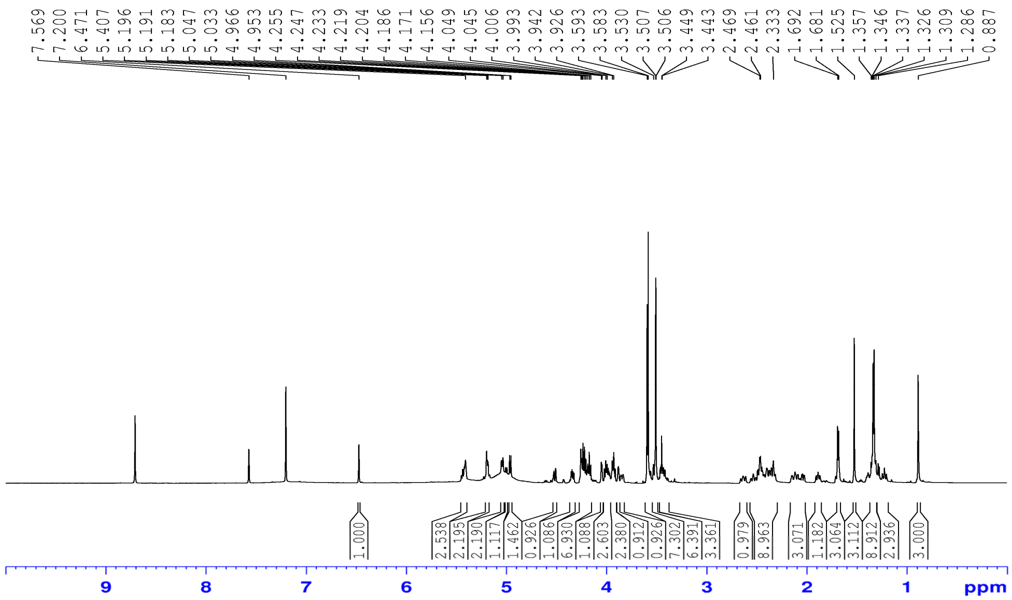

Figure S4. The ^{13}C-NMR spectrum of compound **1**

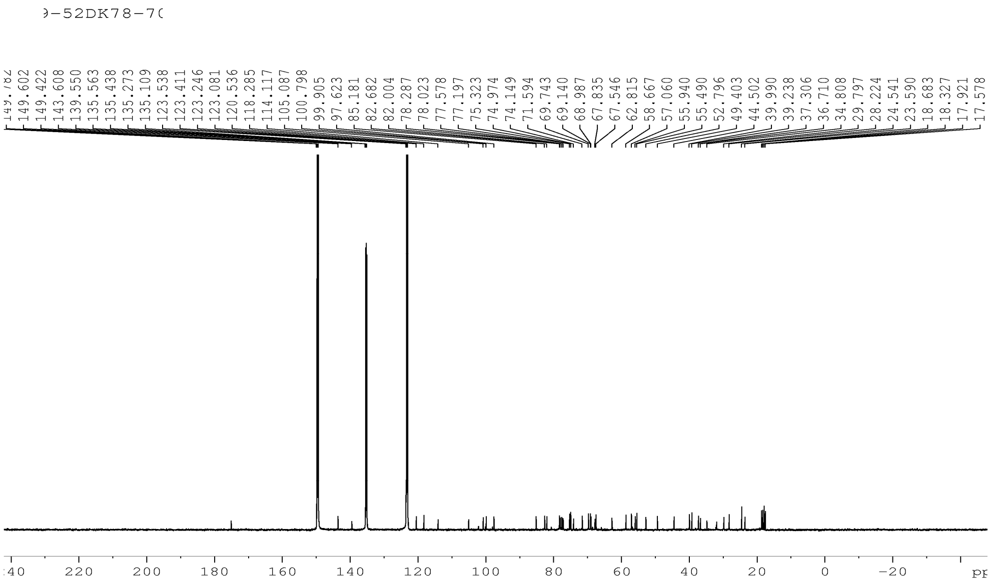

Figure S5. The HSQC spectrum of compound **1**

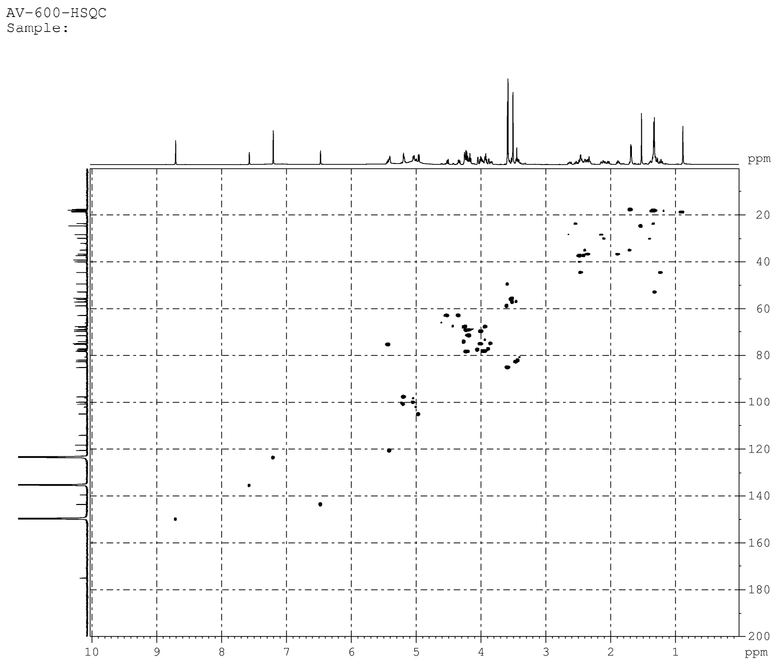

Figure S6. The HMBC spectrum of compound **1**

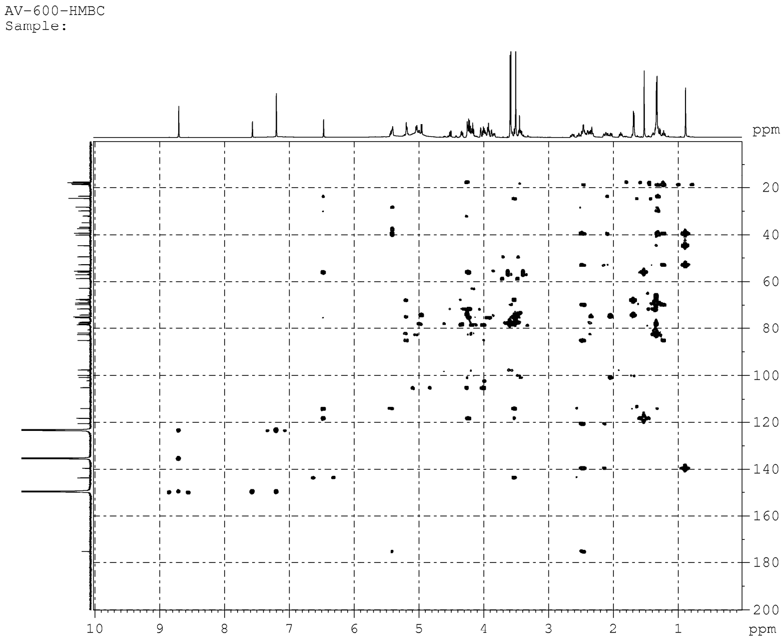

Figure S7. The partial amplified HMBC spectrum of compound **1**

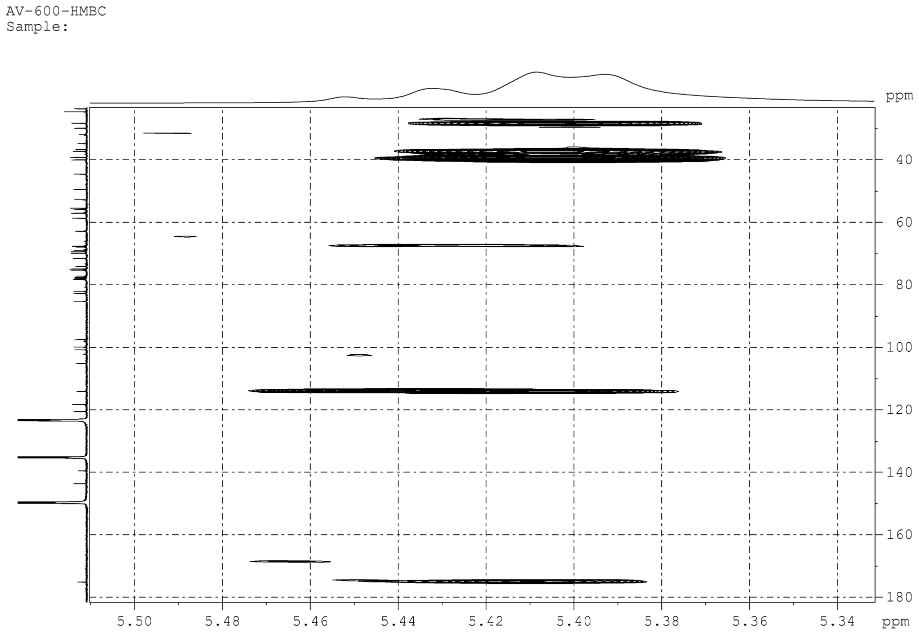

Figure S8. The NOESY spectrum of compound **1**

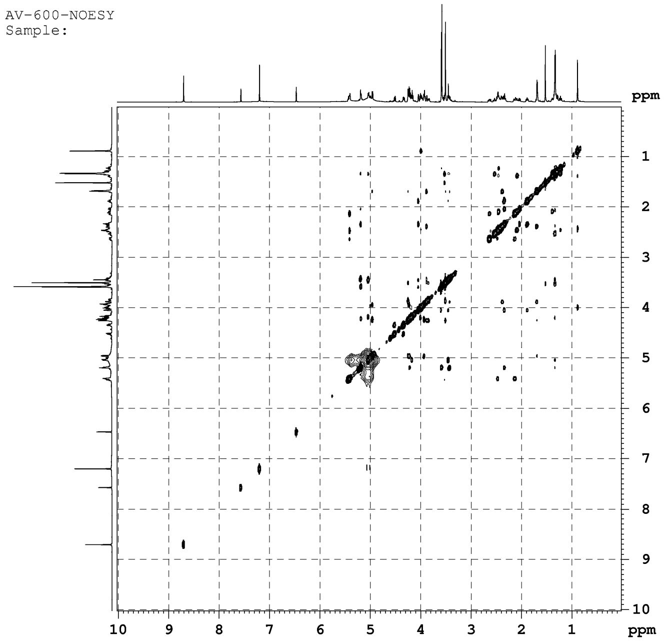

2. The spectra of compound 2

Figure S9. The IR spectrum of compound **2**

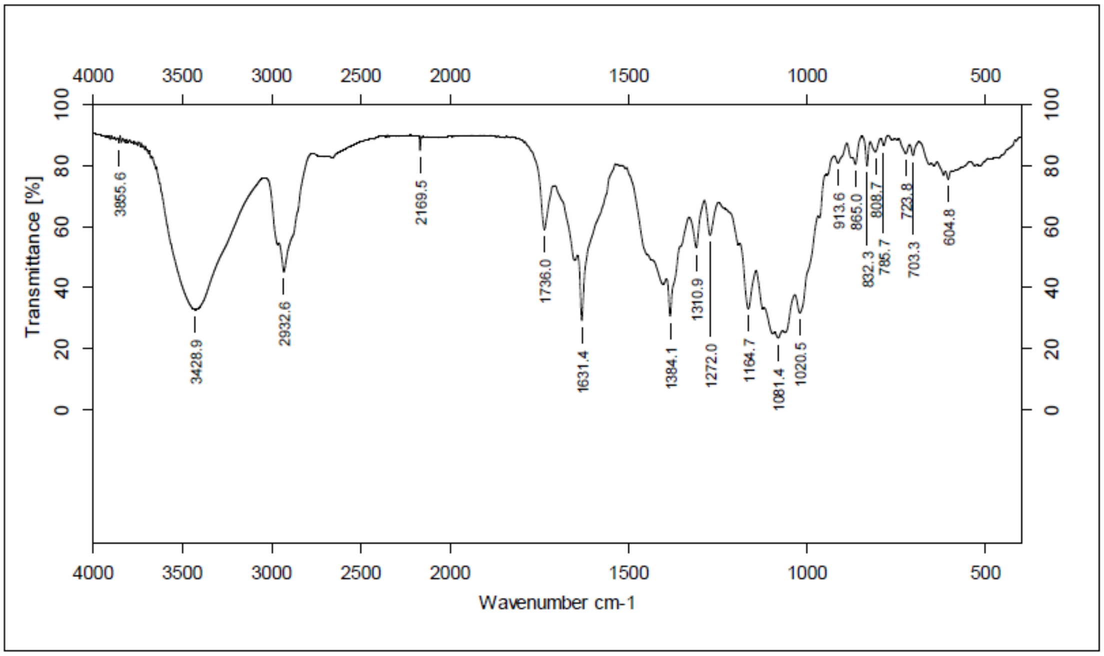

Figure S10. The HR-ESI-MS spectrum of compound **2**

Acquisition Parameter

Source Type	ESI	Ion Polarity	Positive	Set Nebulizer	1.2 Bar
Focus	Active	Set Capillary	4500 V	Set Dry Heater	180 °C
Scan Begin	50 m/z	Set End Plate Offset	-500 V	Set Dry Gas	8.0 l/min
Scan End	1500 m/z	Set Collision Cell RF	400.0 Vpp	Set Divert Valve	Source

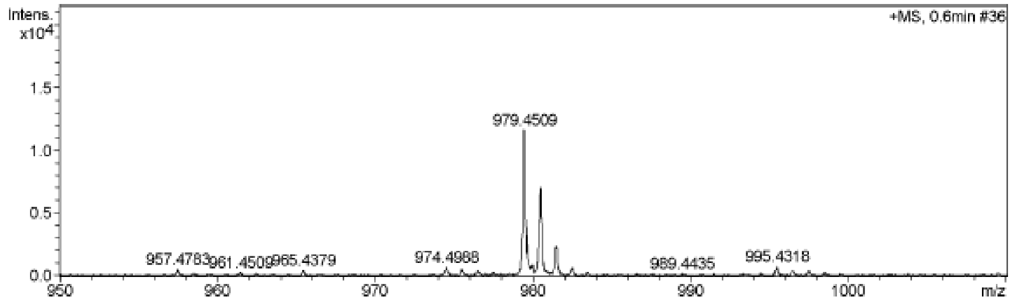

Meas. m/z	#	Formula	m/z	err [ppm]	Mean err [ppm]	rdb	N-Rule	e¯ Conf	mSigma	Std I	Std Mean m/z	Std I VarNorm	Std m/z Diff	Std Comb Dev
979.4509														
	1	C 47 H 72 Na O 20	979.4515	0.6	0.5	11.5	ok	even	39.32	0.0490	0.0017	0.0142	0.0011	0.8427

Figure S11. The ^{1}H-NMR spectrum of compound **2**

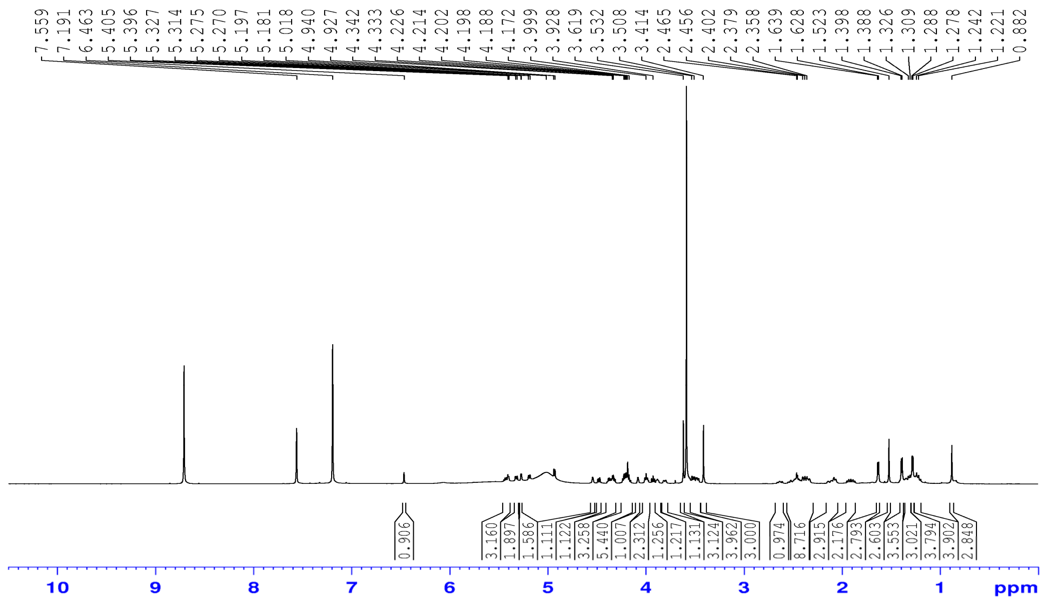

Figure S12. The ^{13}C-NMR spectrum of compound **2**

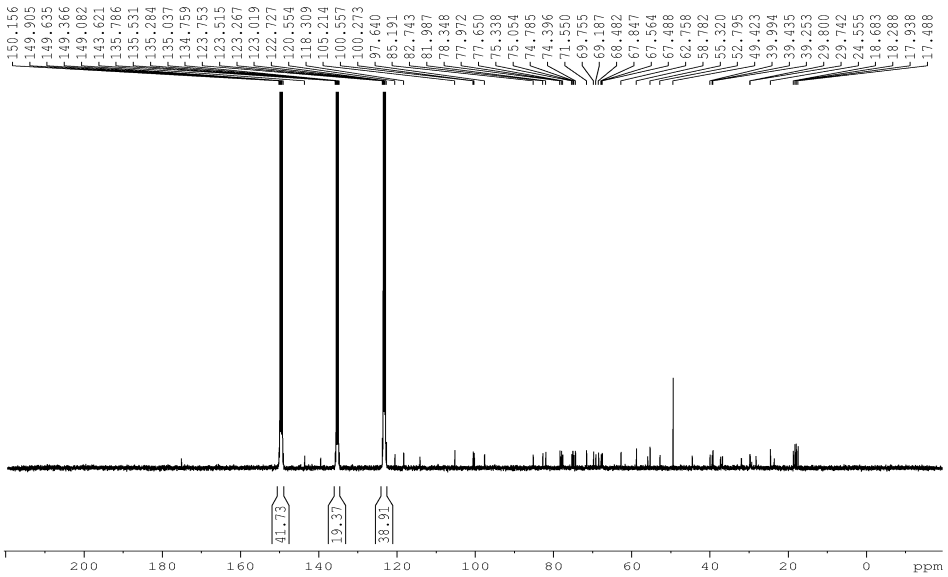

Figure S13. The HSQC spectrum of compound **2**

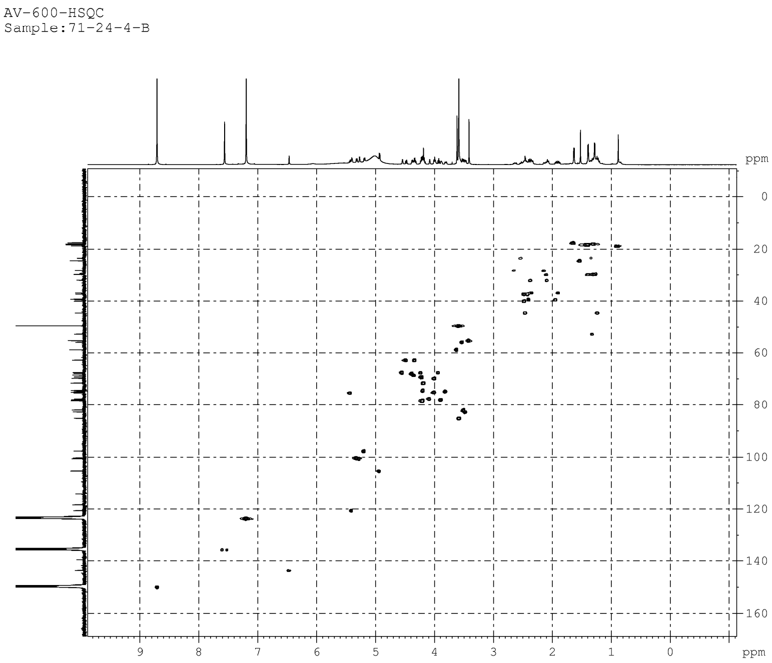

Figure S14. The HMBC spectrum of compound **2**

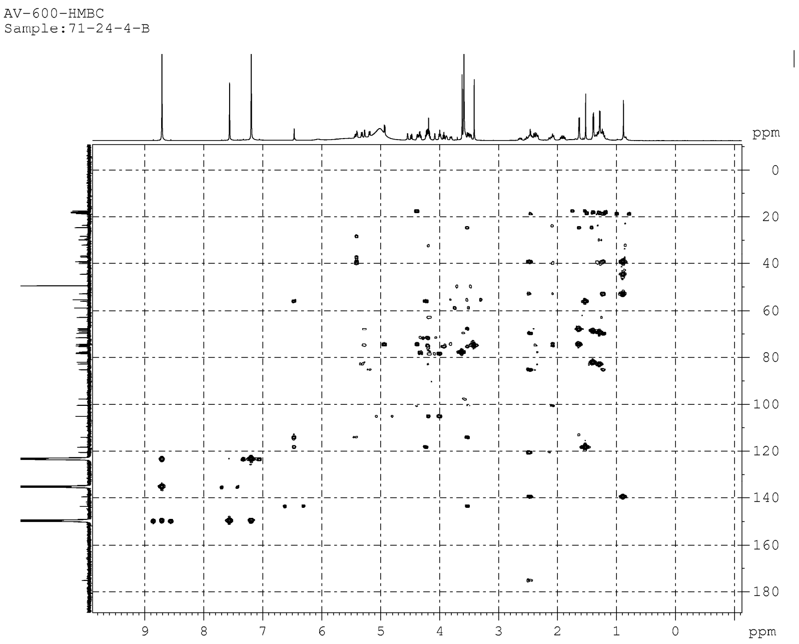

Figure S15. The NOESY spectrum of compound **2**

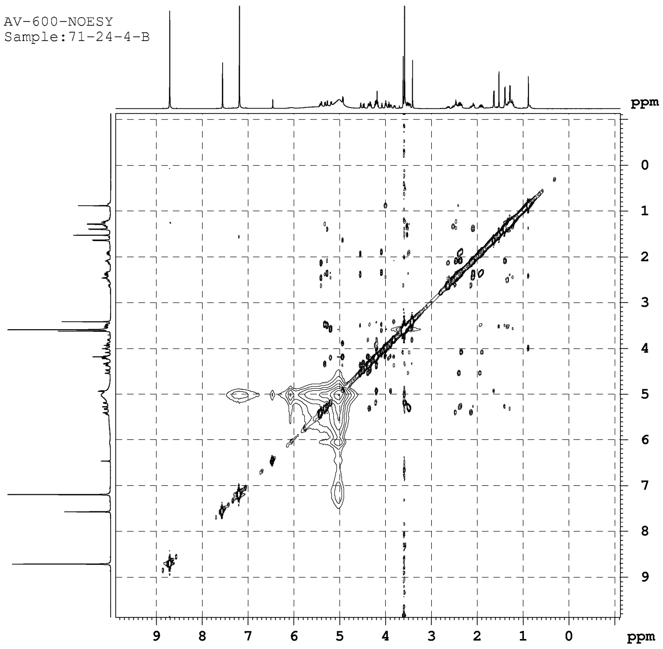

3. The spectra of compound 3

Figure S16. The IR spectrum of compound **3**

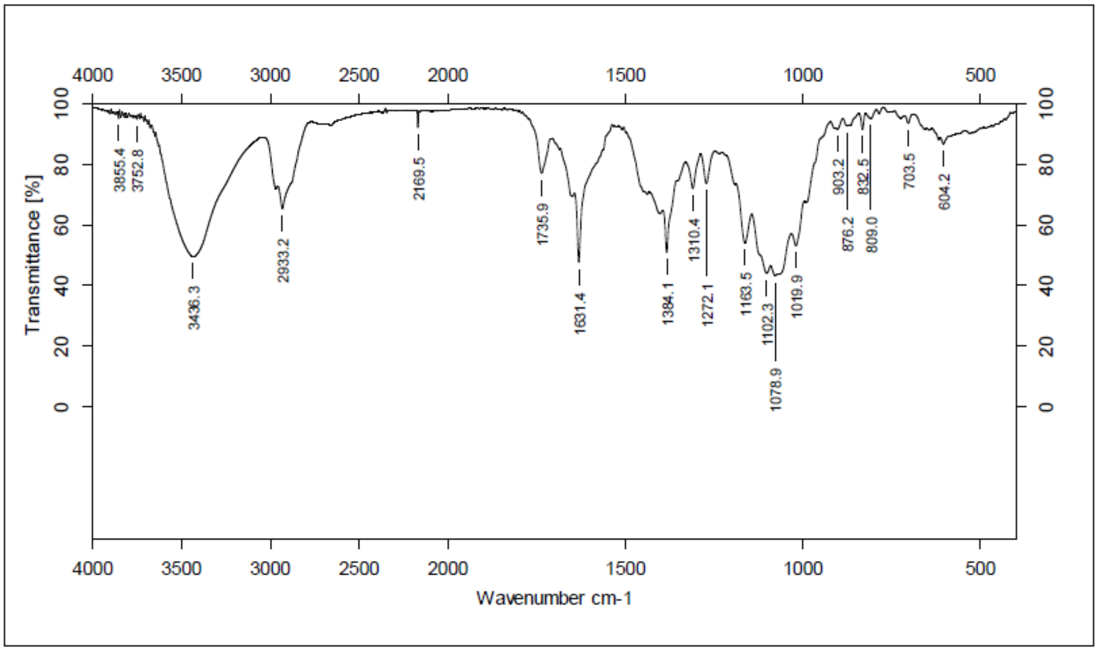

Figure S17. The HR-ESI-MS spectrum of compound **3**

Acquisition Parameter

Source Type	ESI	Ion Polarity	Positive	Set Nebulizer	1.2 Bar
Focus	Active	Set Capillary	4500 V	Set Dry Heater	180 °C
Scan Begin	50 m/z	Set End Plate Offset	-500 V	Set Dry Gas	8.0 l/min
Scan End	1500 m/z	Set Collision Cell RF	400.0 Vpp	Set Divert Valve	Source

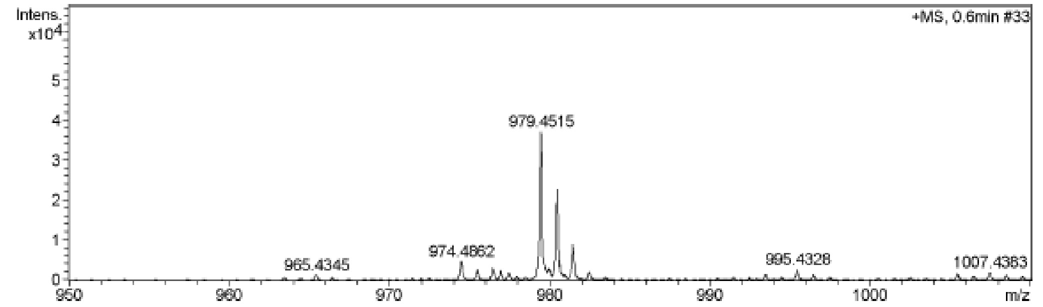

Meas. m/z	#	Formula	m/z	err [ppm]	Mean err [ppm]	rdb	N-Rule	e⁻ Conf	mSigma	Std I	Std Mean m/z	Std I VarNorm	Std m/z Diff	Std Comb Dev
979.4515														
	1	C 47 H 72 Na O 20	979.4515	-0.1	1.5	11.5	ok	even	50.99	0.0646	0.0026	0.0186	0.0041	0.8427

Figure S18. The ^{1}H-NMR spectrum of compound **3**

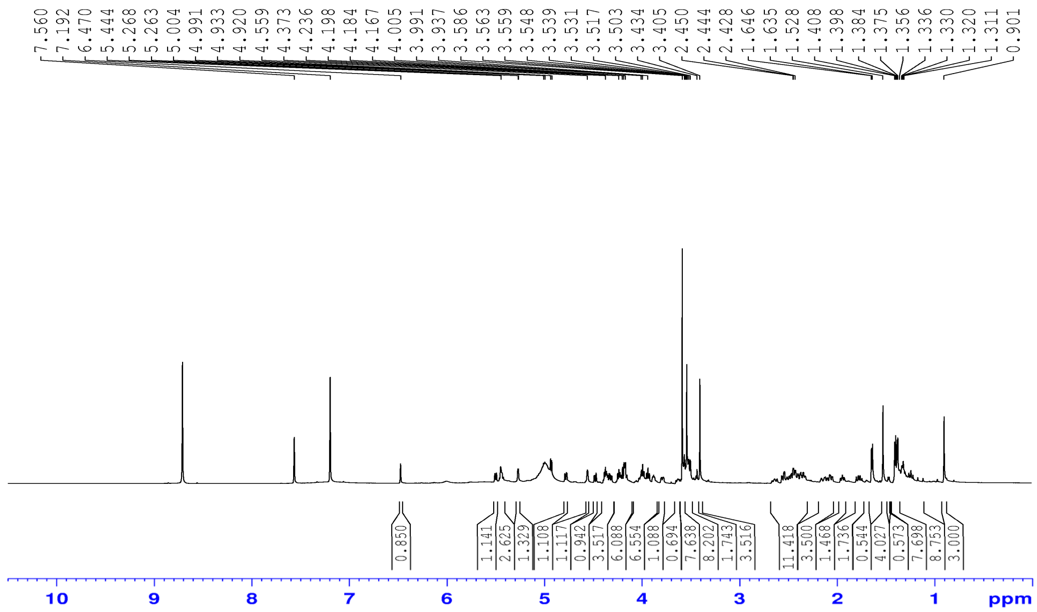

Figure S19. The ^{13}C-NMR spectrum of compound **3**

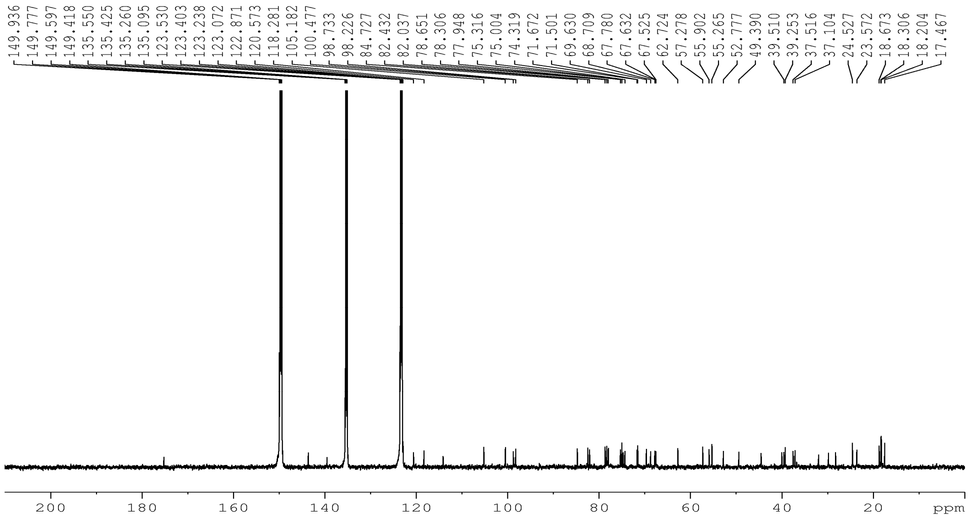

Figure S20. The HSQC spectrum of compound **3**

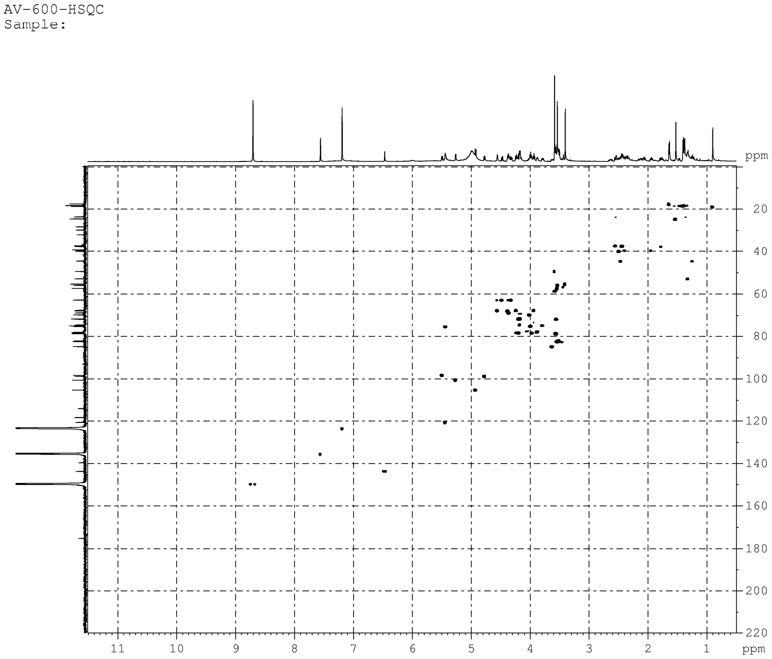

Figure S21. The HMBC spectrum of compound **3**

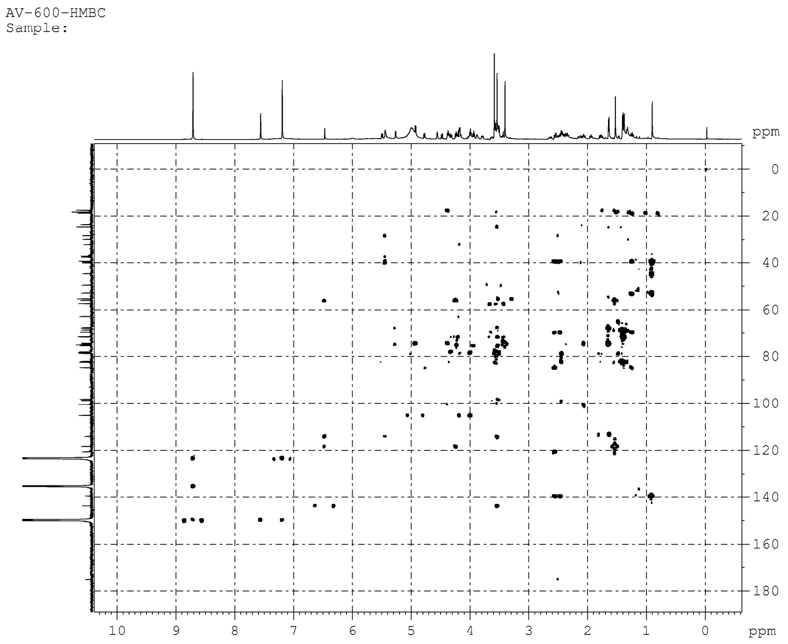

Figure S22. The NOESY spectrum of compound **3**

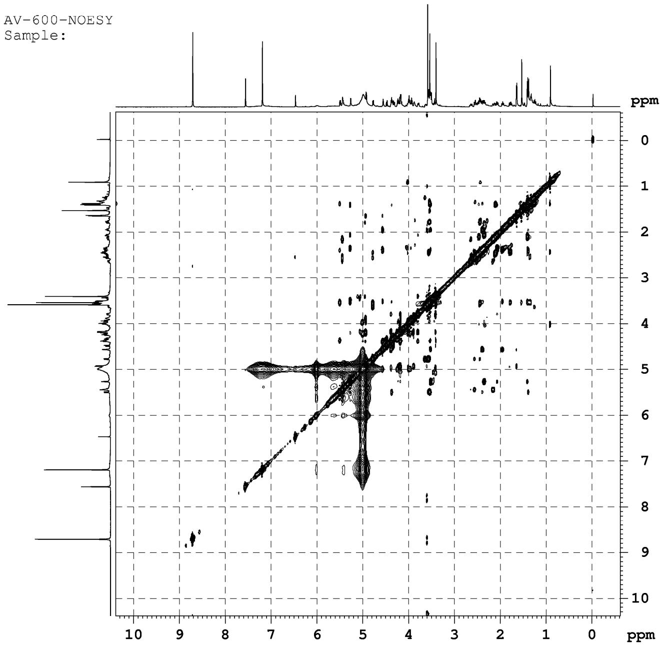

4. The spectra of compound 4

Figure S23. The IR spectrum of compound **4**

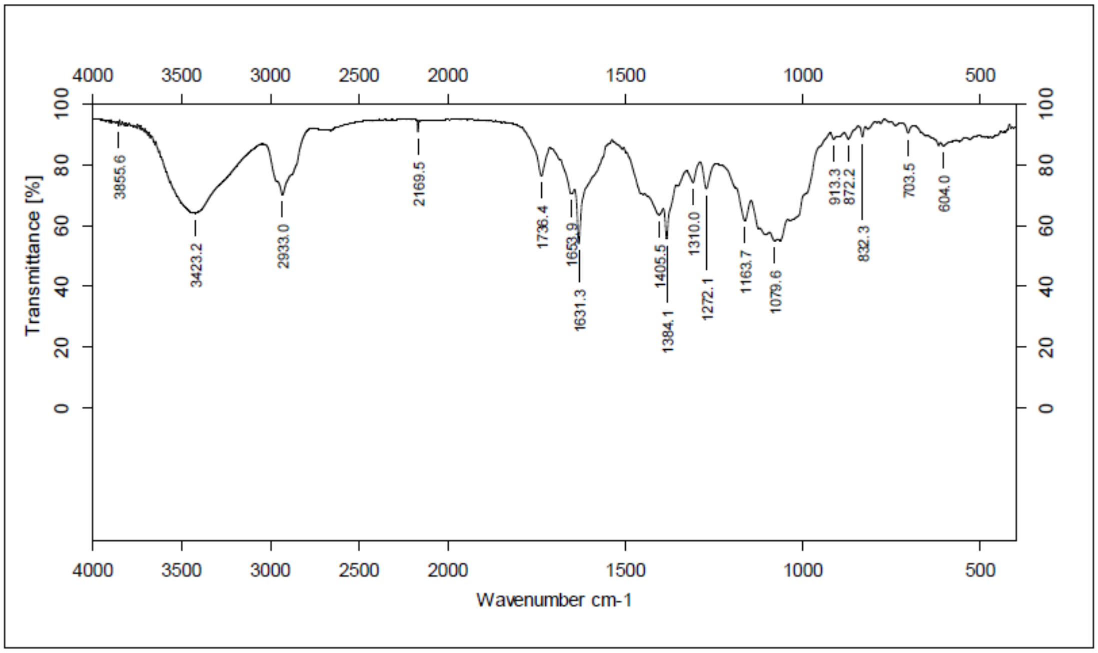

Figure S24. The HR-ESI-MS spectrum of compound **4**

Acquisition Parameter

Source Type	ESI	Ion Polarity	Positive	Set Nebulizer	1.2 Bar
Focus	Active	Set Capillary	4500 V	Set Dry Heater	180 °C
Scan Begin	50 m/z	Set End Plate Offset	-500 V	Set Dry Gas	8.0 l/min
Scan End	1500 m/z	Set Collision Cell RF	400.0 Vpp	Set Divert Valve	Source

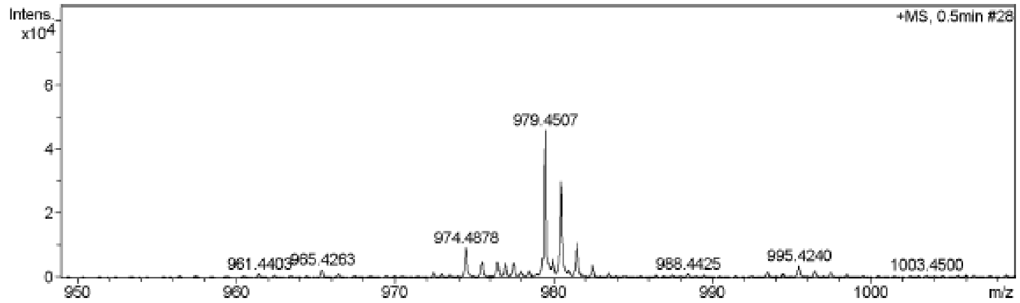

Meas. m/z	#	Formula	m/z	err [ppm]	Mean err [ppm]	rdb	N-Rule	e⁻ Conf	mSigma	Std I	Std Mean m/z	Std I VarNorm	Std m/z Diff	Std Comb Dev
979.4507														
	1	C 47 H 72 Na O 20	979.4515	0.8	2.9	11.5	ok	even	66.63	0.0823	0.0047	0.0242	0.0058	0.8427

Figure S25. The ^{1}H-NMR spectrum of compound **4**

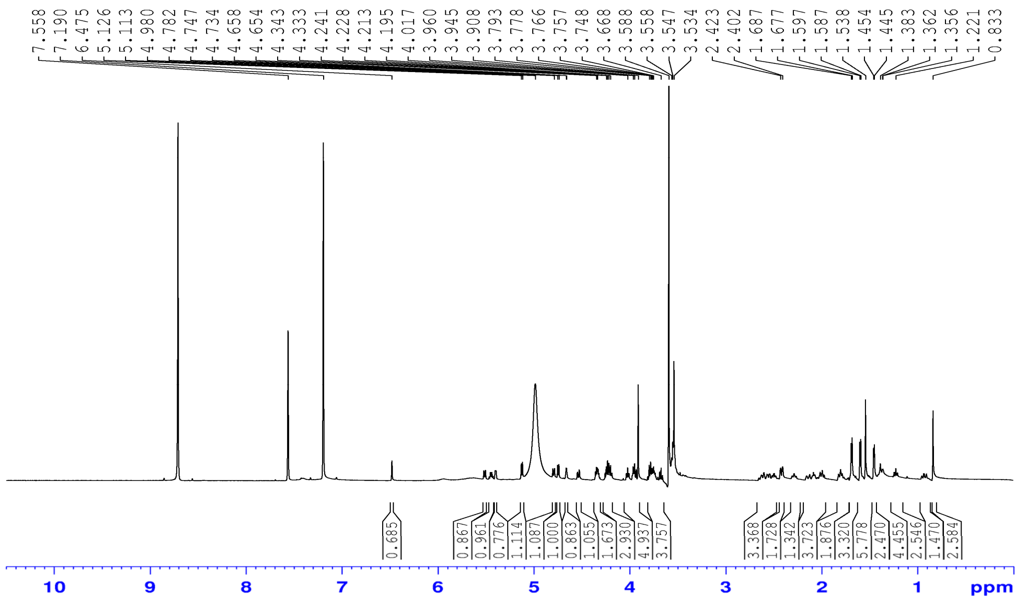

Figure S26. The ^{13}C-NMR spectrum of compound **4**

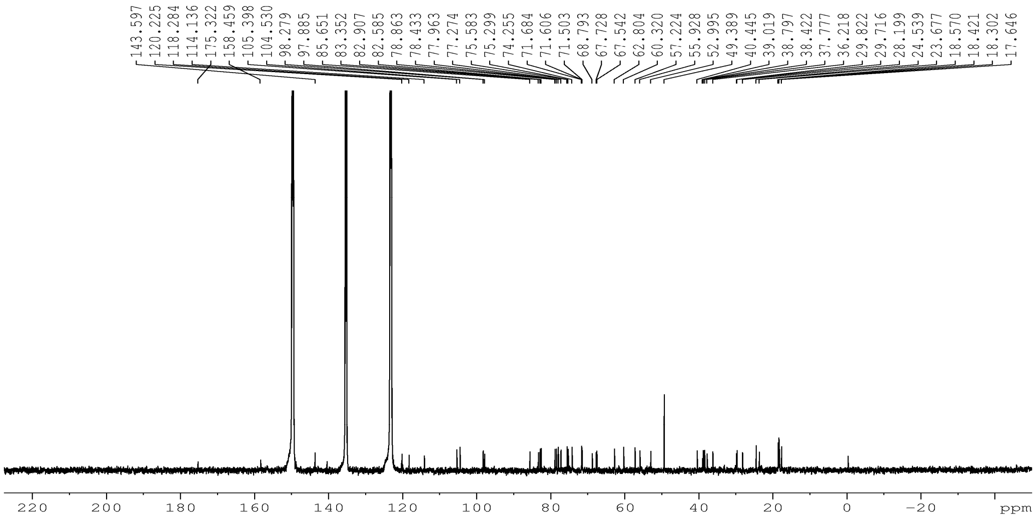

Figure S27. The HSQC spectrum of compound **4**

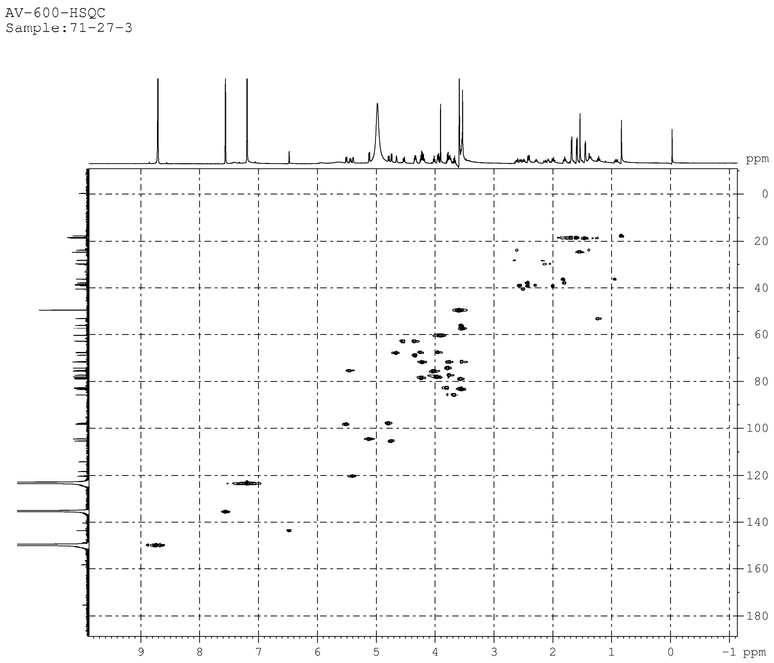

Figure S28. The HMBC spectrum of compound **4**

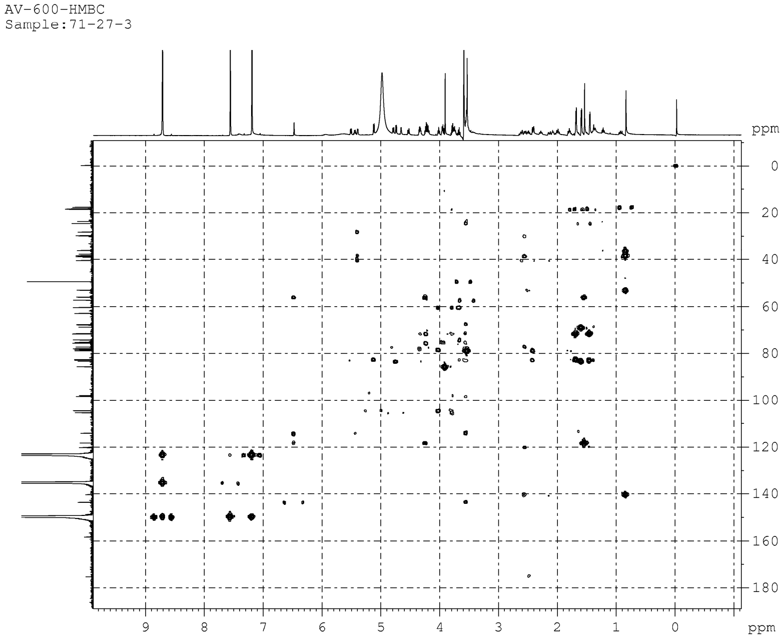

Figure S29. The NOESY spectrum of compound **4**

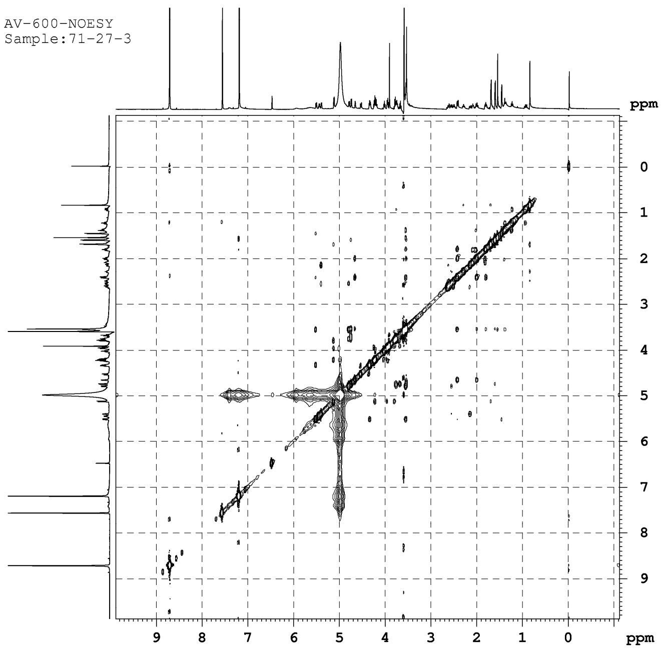

5. The spectra of compound 7

Figure S30. The IR spectrum of compound **7**

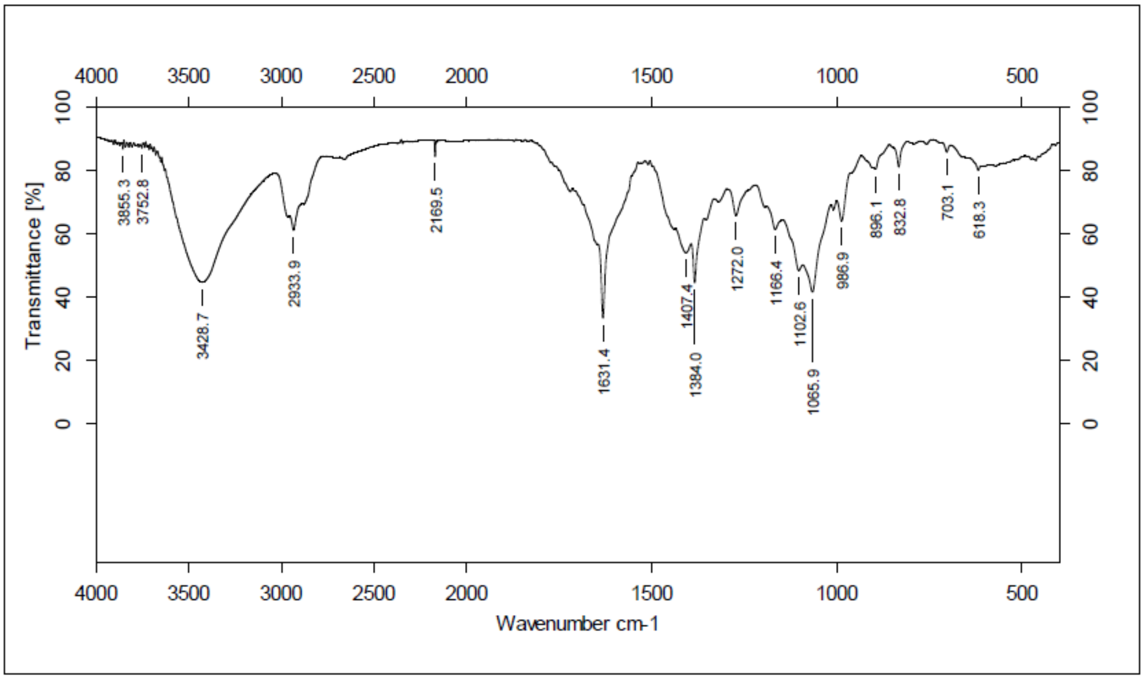

Figure S31. The HR-ESI-MS spectrum of compound **7**

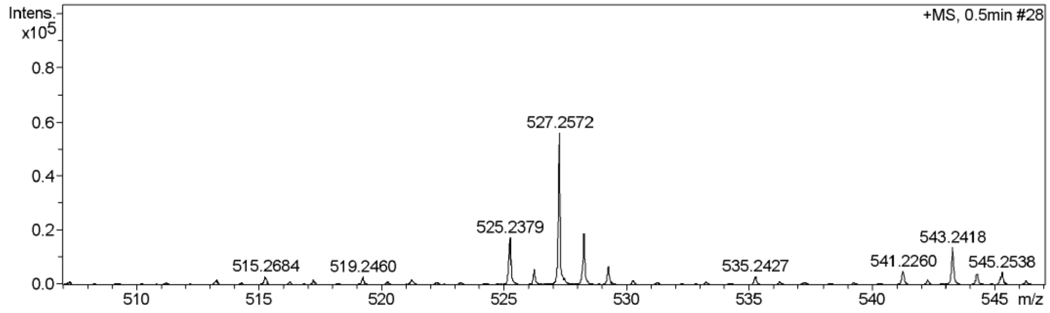

Meas. m/z	#	Formula	m/z	err [ppm]	Mean err [ppm]	rdb	N-Rule	e⁻ Conf	mSigma	Std I	Std Mean m/z	Std I VarNorm	Std m/z Diff	Std Comb Dev
527.2572														
	1	C 28 H 40 Na O 8	527.2621	9.2	11.7	8.5	ok	even	34.29	0.0647	0.0064	0.0258	0.0037	0.8427

Figure S32. The ^{1}H-NMR spectrum of compound **7**

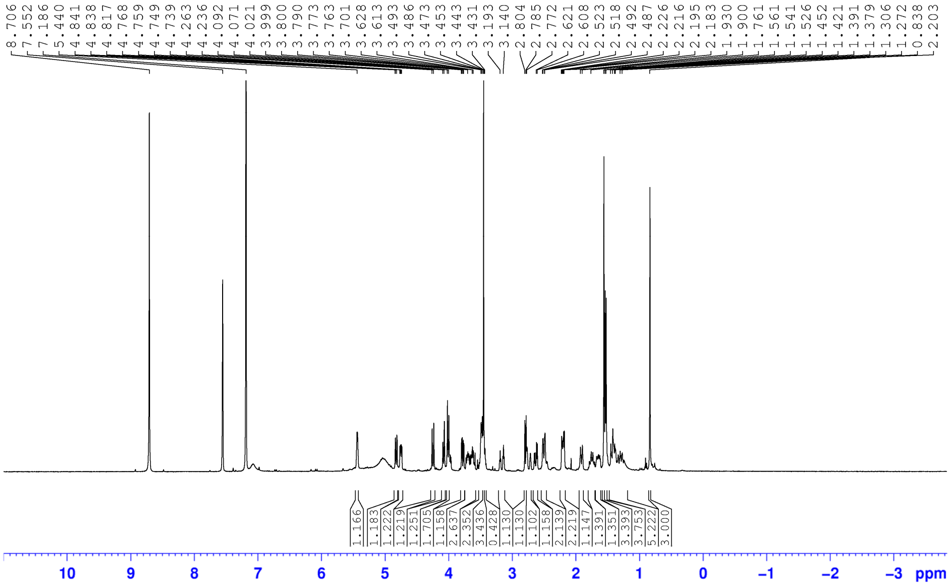

Figure S33. The ^{13}C-NMR spectrum of compound **7**

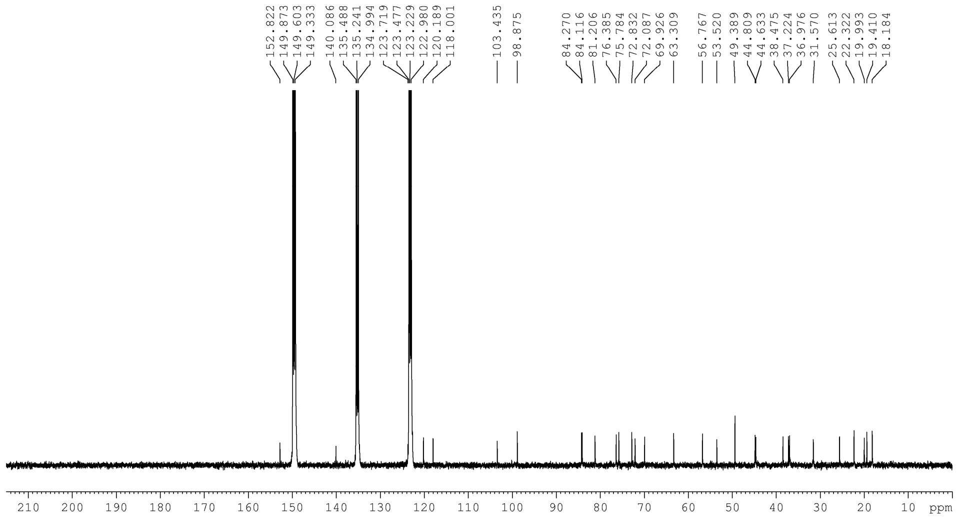

Figure S34. The HSQC spectrum of compound **7**

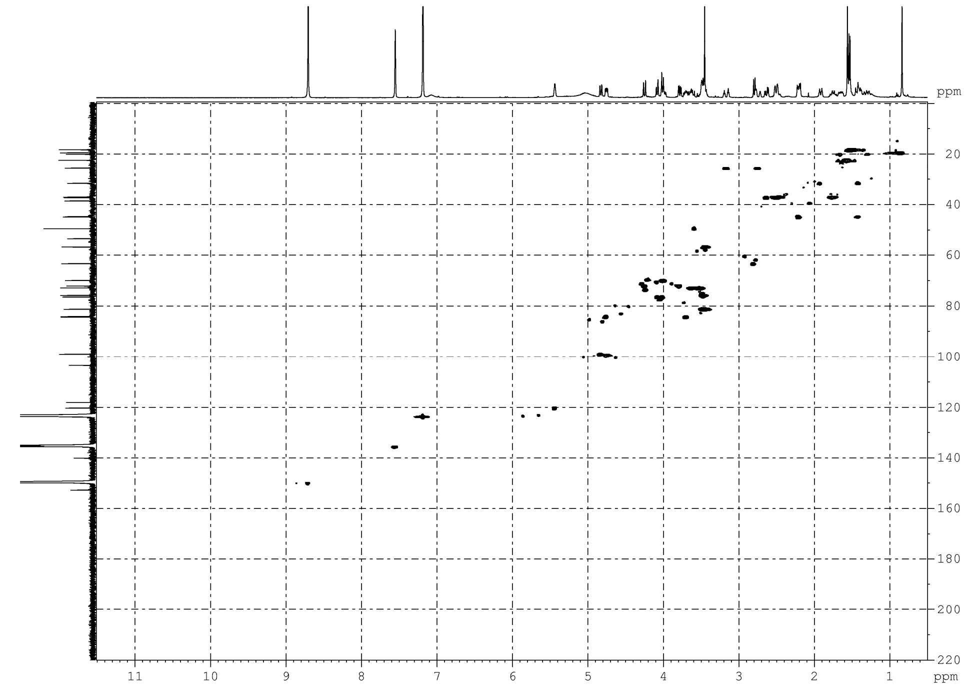

Figure S35. The HMBC spectrum of compound **7**

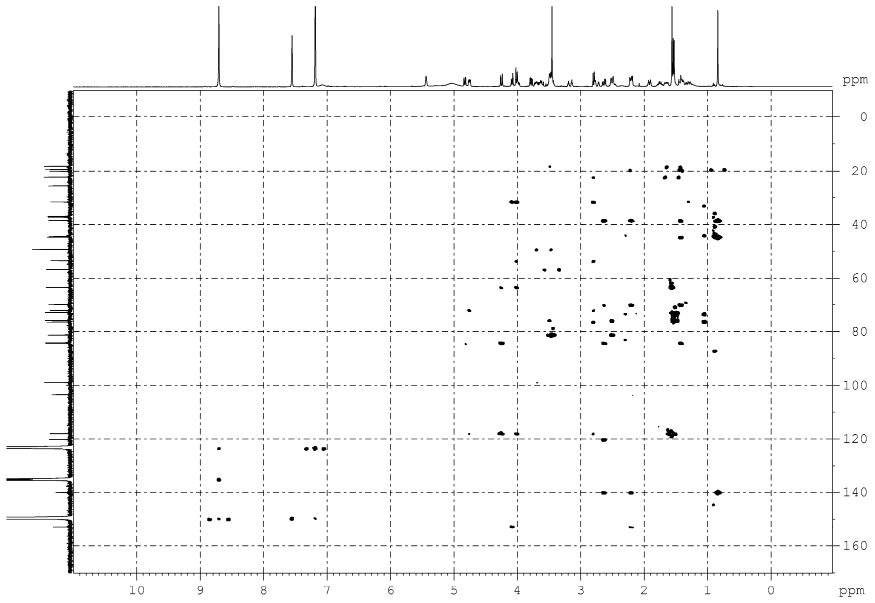

Figure S36. The NOESY spectrum of compound **7**

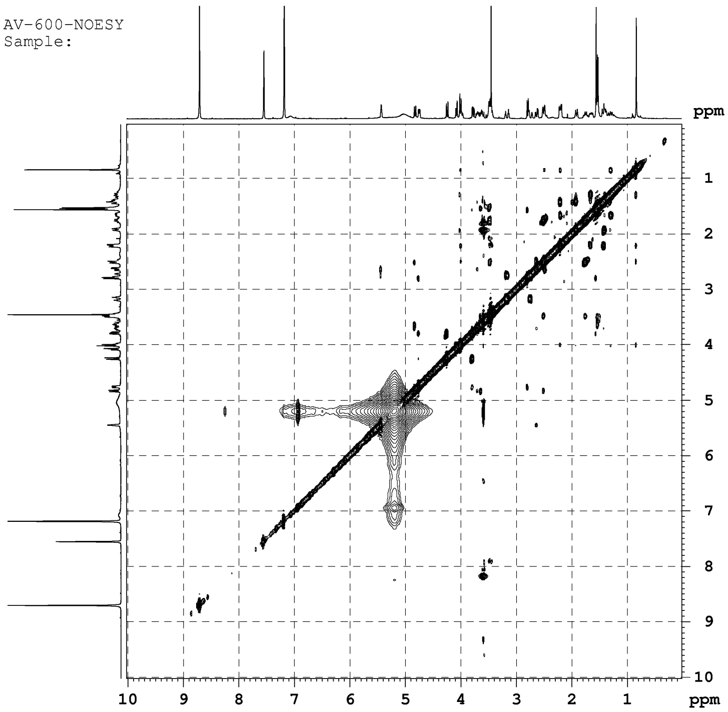

6. The spectra of compound 8

Figure S37. The IR spectrum of compound **8**

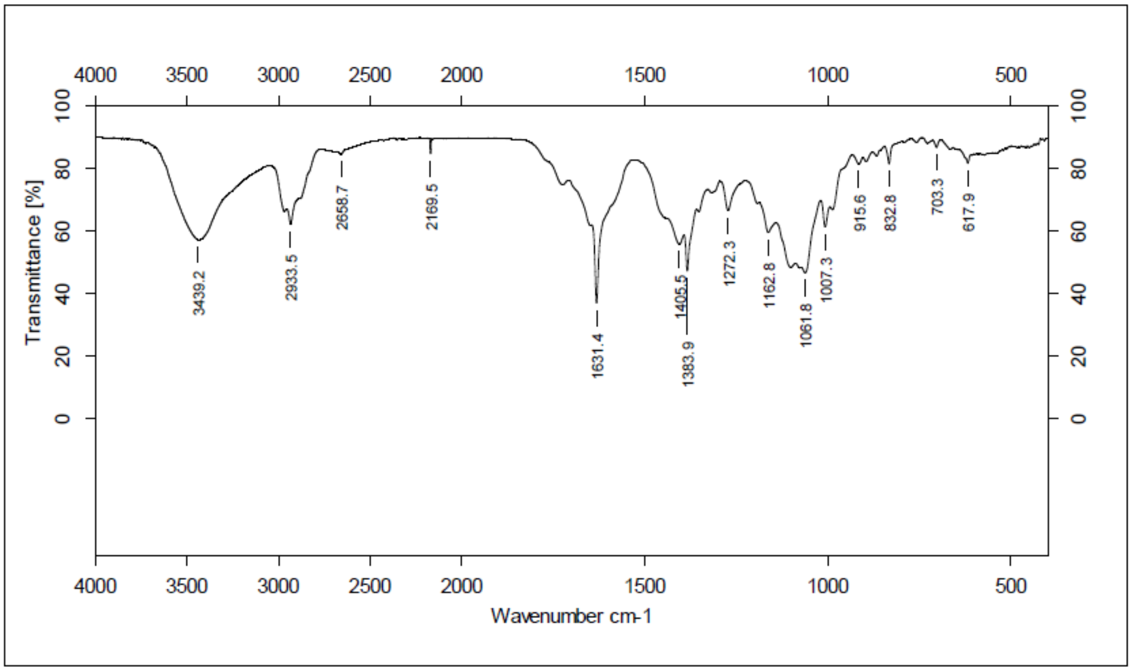

Figure S38. The HR-ESI-MS spectrum of compound **8**

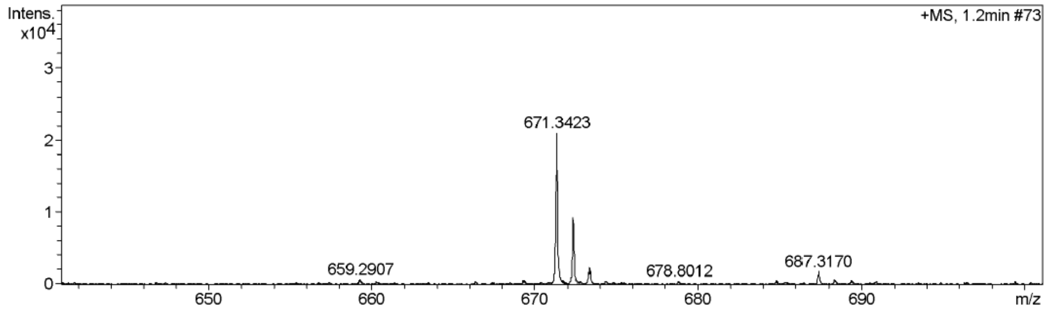

Meas. m/z	#	Formula	m/z	err [ppm]	Mean err [ppm]	rdb	N-Rule	e⁻ Conf	mSigma	Std I	Std Mean m/z	Std I VarNorm	Std m/z Diff	Std Comb Dev
671.3423														
	1	C 35 H 52 Na O 11	671.3407	-2.3	-1.4	9.5	ok	even	27.00	0.0383	0.0013	0.0122	0.0017	0.8427

Figure S39. The ^{1}H-NMR spectrum of compound **8**

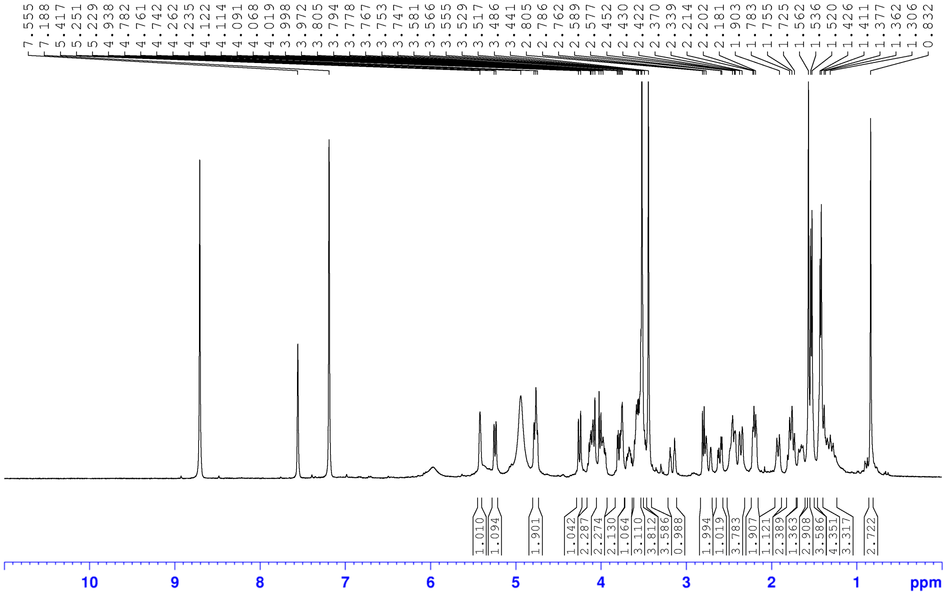

Figure S40. The ^{13}C-NMR spectrum of compound **8**

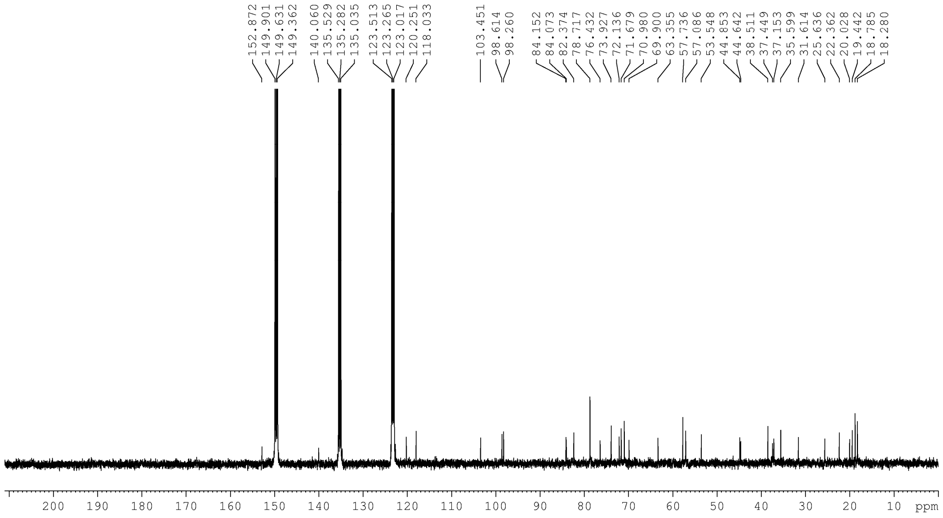

Figure S41. The HSQC spectrum of compound **8**

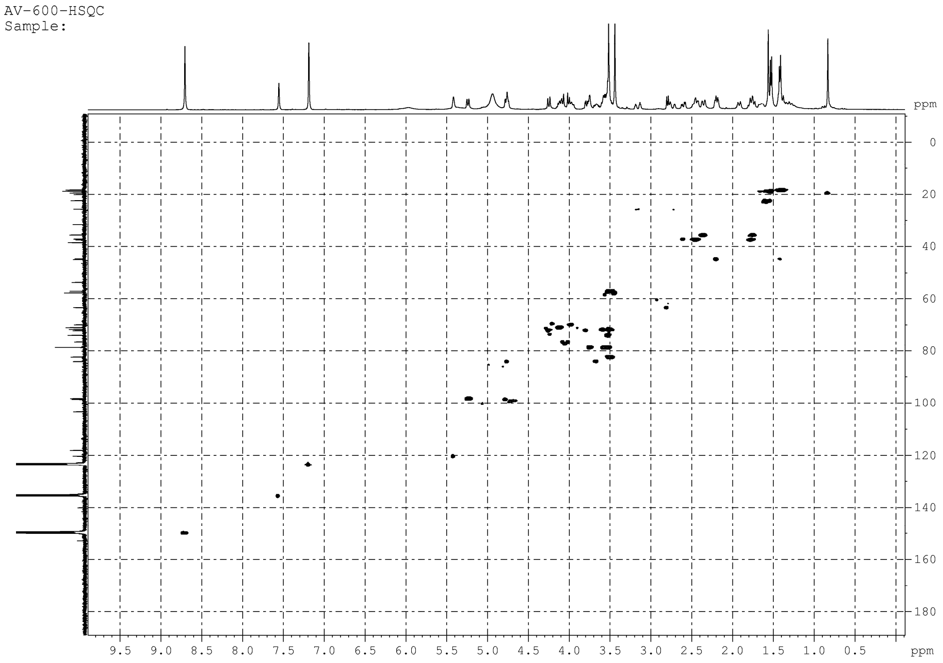

Figure S42. The HMBC spectrum of compound **8**

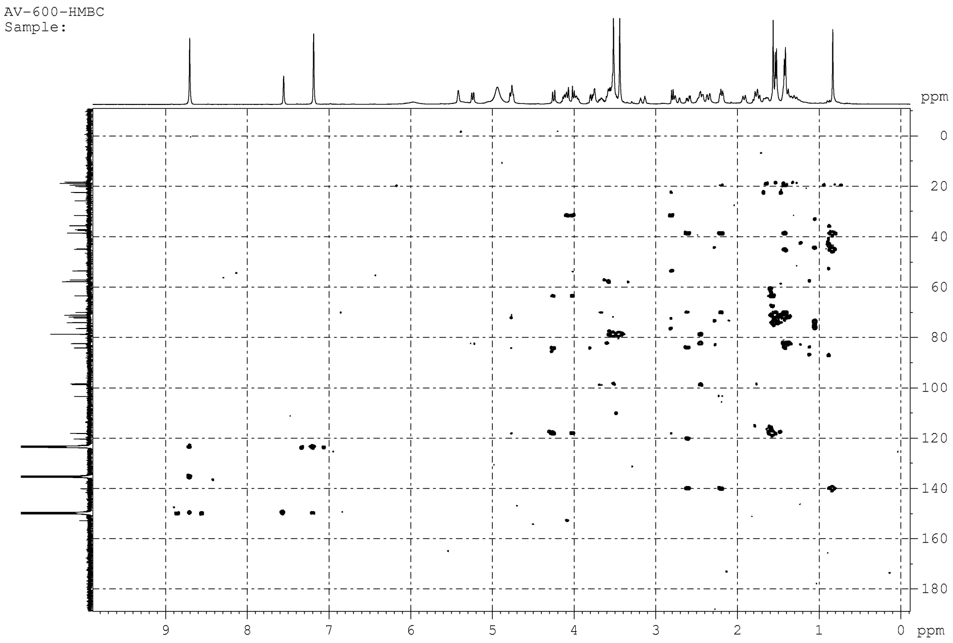

Figure S43. The NOESY spectrum of compound **8**

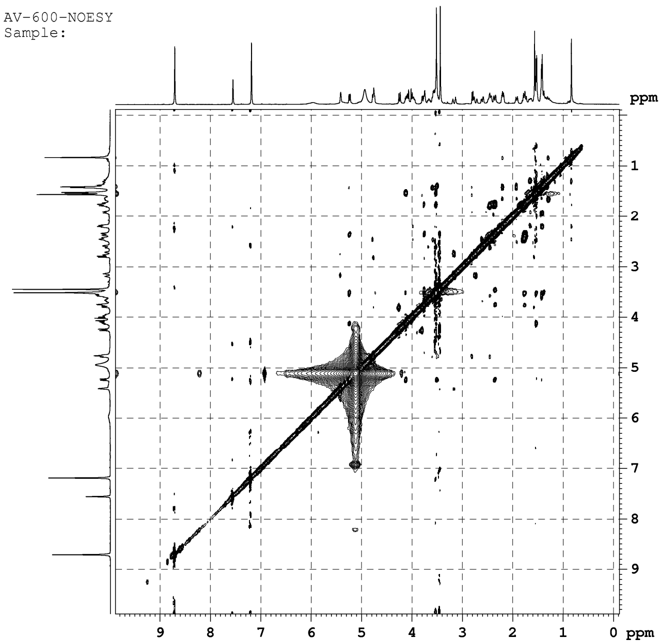

7. The spectra of compound 9

Figure S44. The HR-ESI-MS spectrum of compound **9**

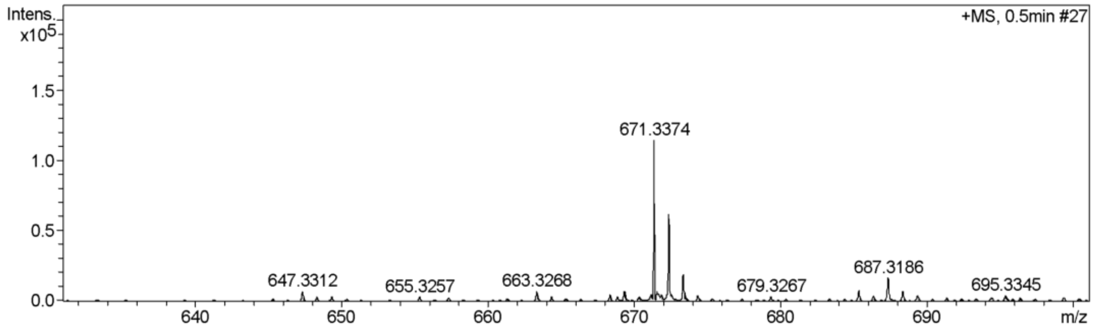

Meas. m/z	#	Formula	m/z	err [ppm]	Mean err [ppm]	rdb	N-Rule	e⁻ Conf	mSigma	Std I	Std Mean m/z	Std I VarNorm	Std m/z Diff	Std Comb Dev
671.3374														
	1	C 35 H 52 Na O 11	671.3407	4.9	6.0	9.5	ok	even	84.86	0.1113	0.0045	0.0361	0.0039	0.8427

Figure S45. The ^{1}H-NMR spectrum of compound **9**

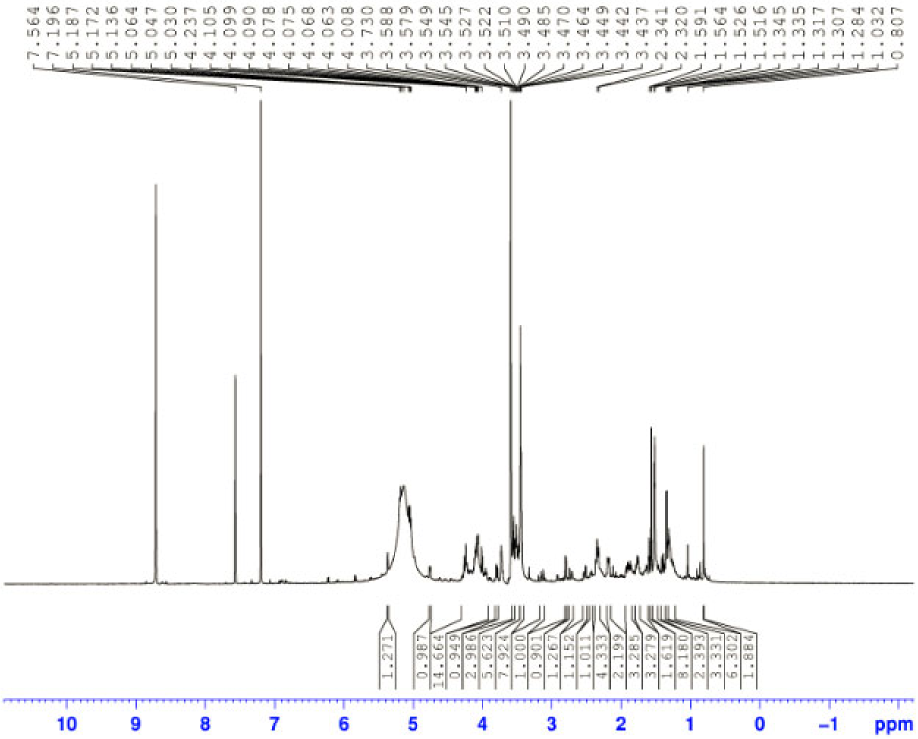

Figure S46. The ^{13}C-NMR spectrum of compound **9**

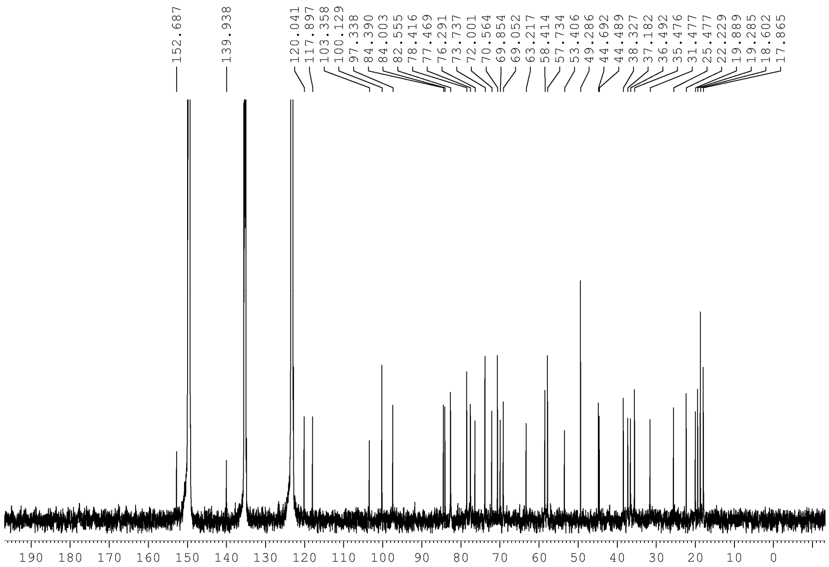

Figure S47. The HSQC spectrum of compound **9**

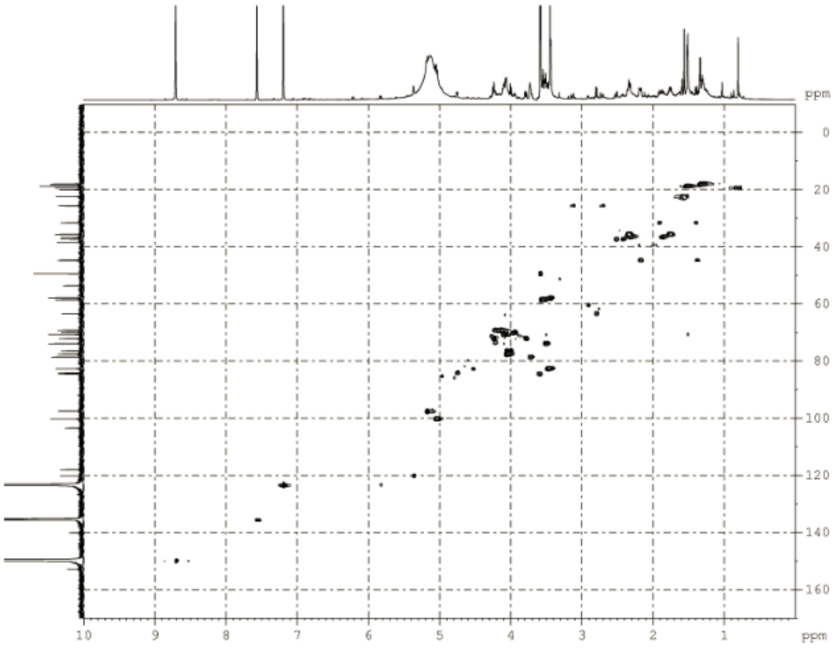

Figure S48. The HMBC spectrum of compound **9**

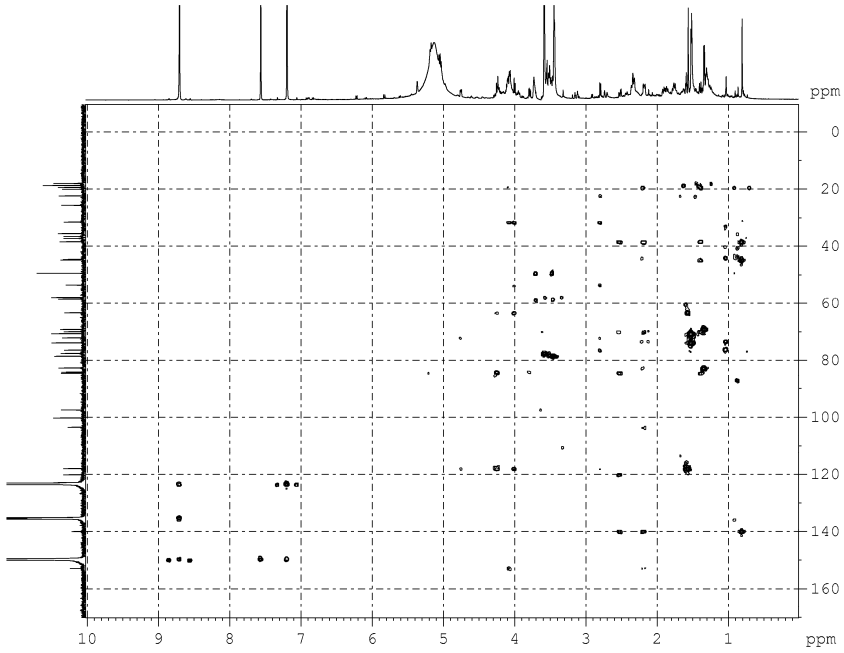

Figure S49. The NOESY spectrum of compound **9**

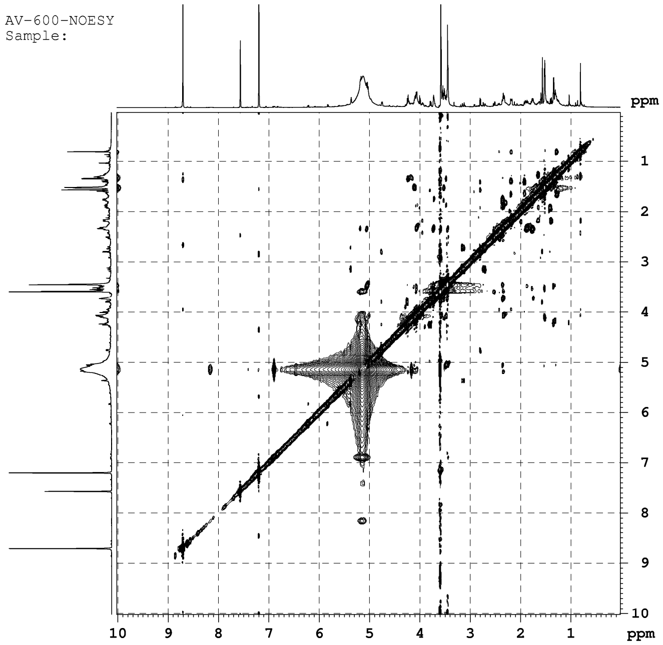

8. The spectra of compound 10

Figure S50. The IR spectrum of compound **10**

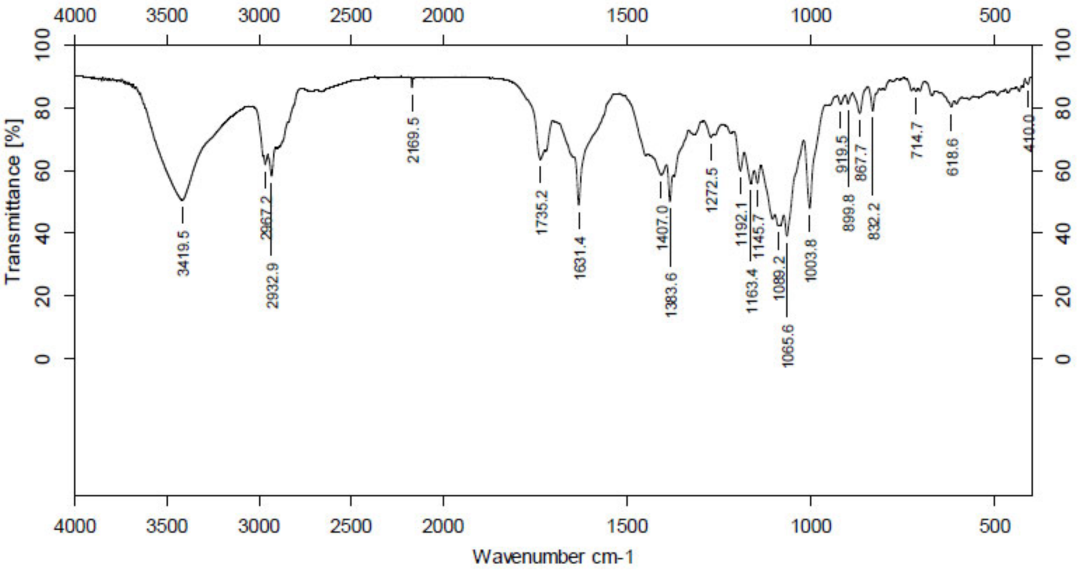

Figure S51. The HR-ESI-MS spectrum of compound **10**

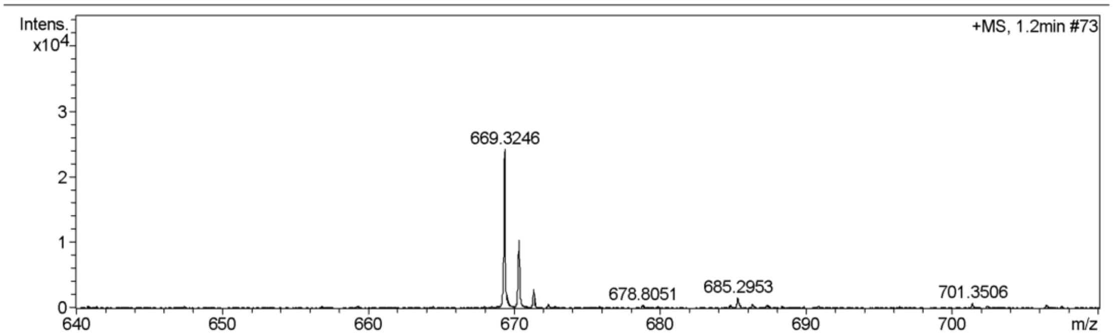

Meas. m/z	#	Formula	m/z	err [ppm]	Mean err [ppm]	rdb	N-Rule	e⁻ Conf	mSigma	Std I	Std Mean m/z	Std I VarNorm	Std m/z Diff	Std Comb Dev
669.3246														
	1	C 35 H 50 Na O 11	669.3251	0.7	2.8	10.5	ok	even	25.08	0.0378	0.0026	0.0127	0.0034	0.8427

Figure S52. The ^{1}H-NMR spectrum of compound **10**

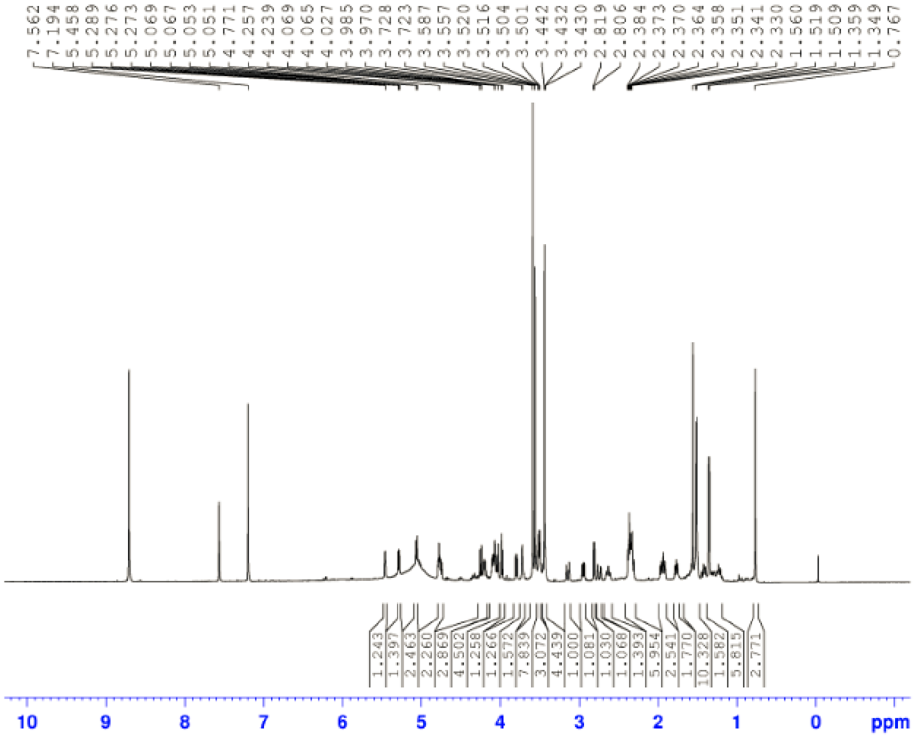

Figure S53. The ^{13}C-NMR spectrum of compound **10**

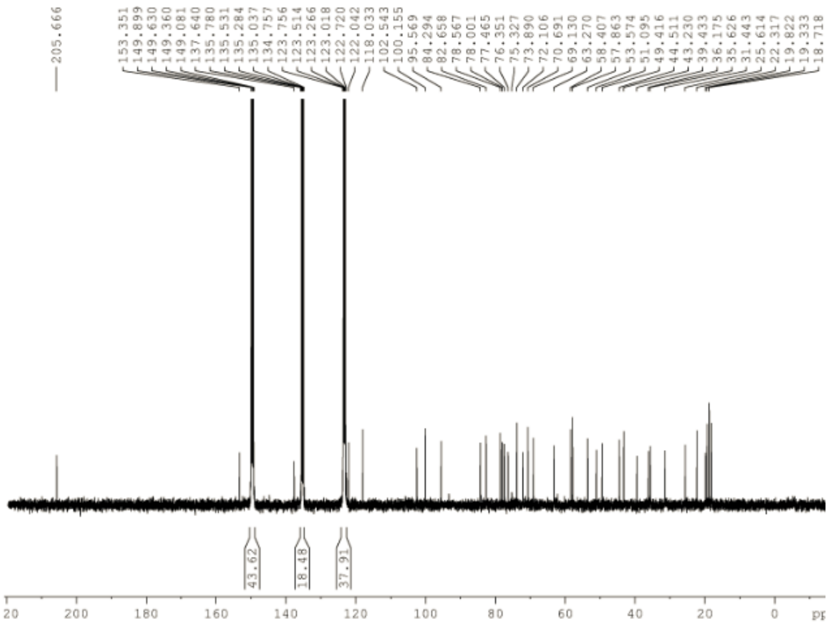

Figure S54. The HSQC spectrum of compound **10**

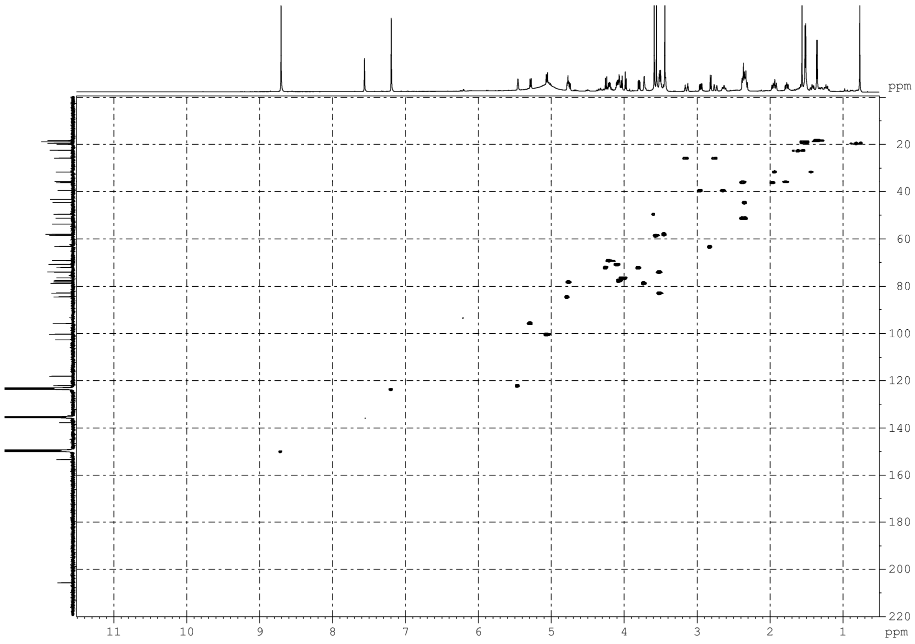

Figure S55. The HMBC spectrum of compound **10**

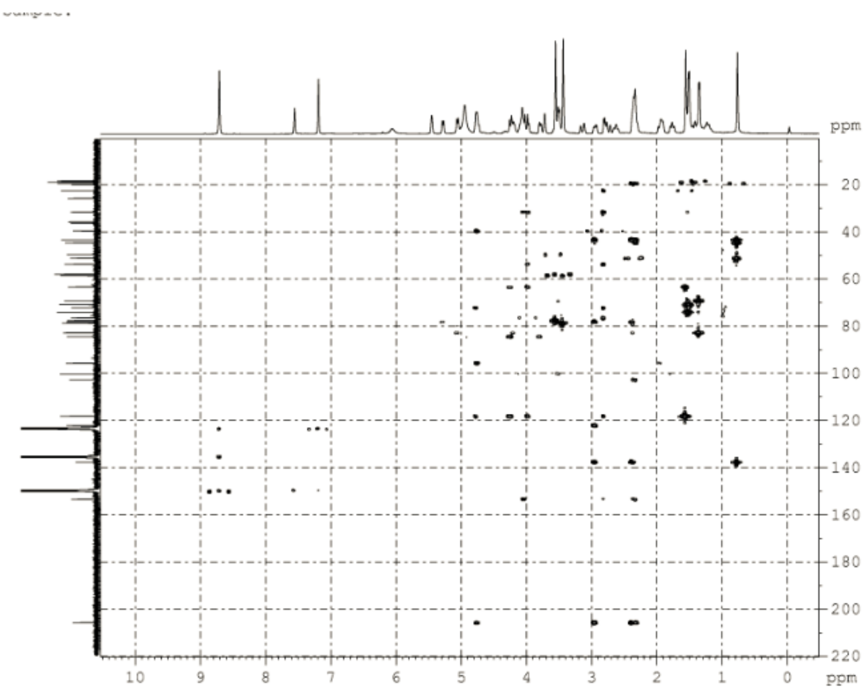

Figure S56. The NOESY spectrum of compound **10**

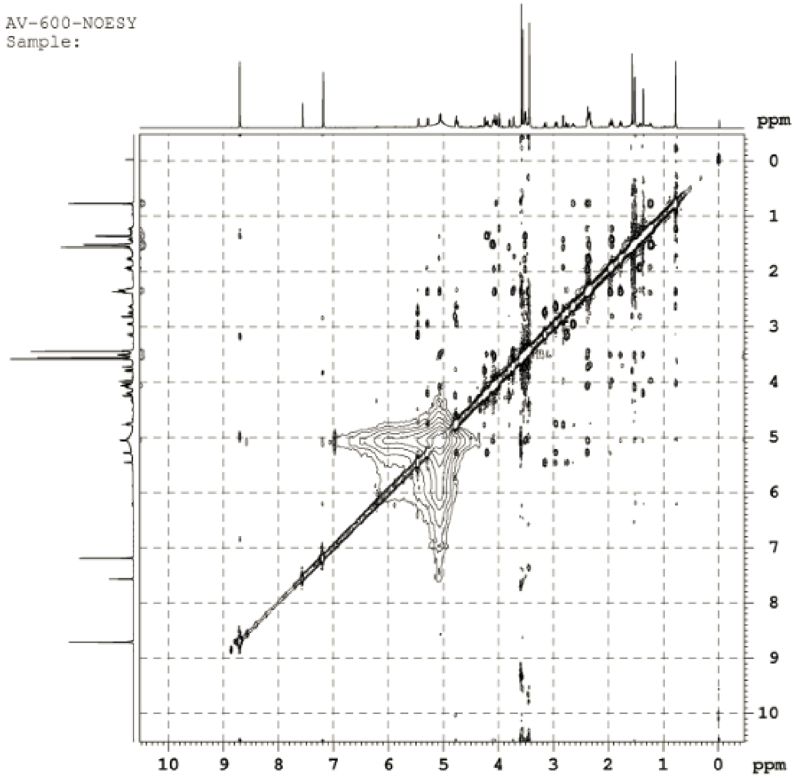

9. The spectra of compound 11

Figure S57. The IR spectrum of compound **11**

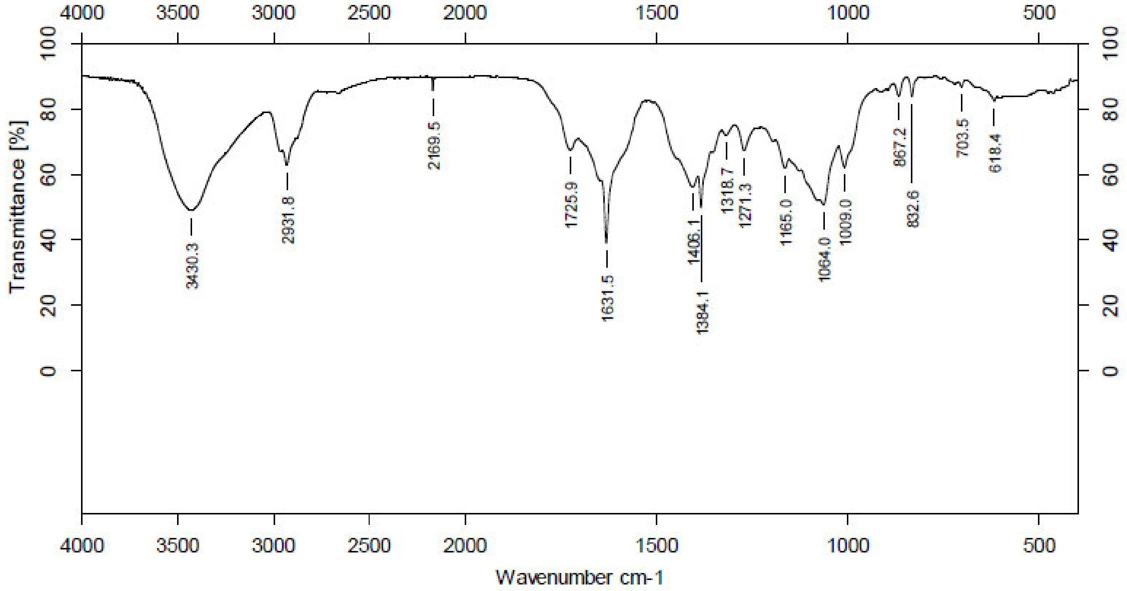

Figure S58. The HR-ESI-MS spectrum of compound **11**

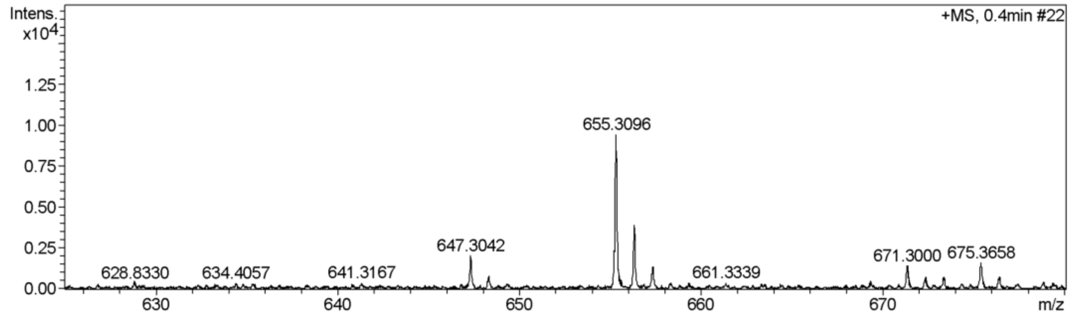

Meas. m/z	#	Formula	m/z	err [ppm]	Mean err [ppm]	rdb	N-Rule	e⁻ Conf	mSigma	Std I	Std Mean m/z	Std I VarNorm	Std m/z Diff	Std Comb Dev
655.3096														
	1	C 34 H 48 Na O 11	655.3094	-0.2	1.6	10.5	ok	even	36.12	0.0598	0.0020	0.0229	0.0025	0.8427

Figure S59. The ^{1}H-NMR spectrum of compound **11**

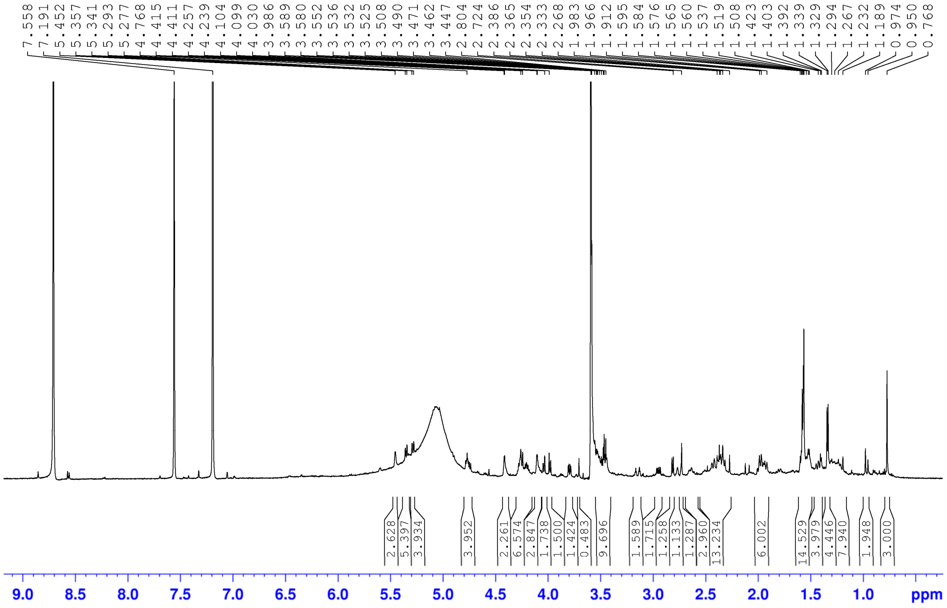

Figure S60. The ^{13}C-NMR spectrum of compound **11**

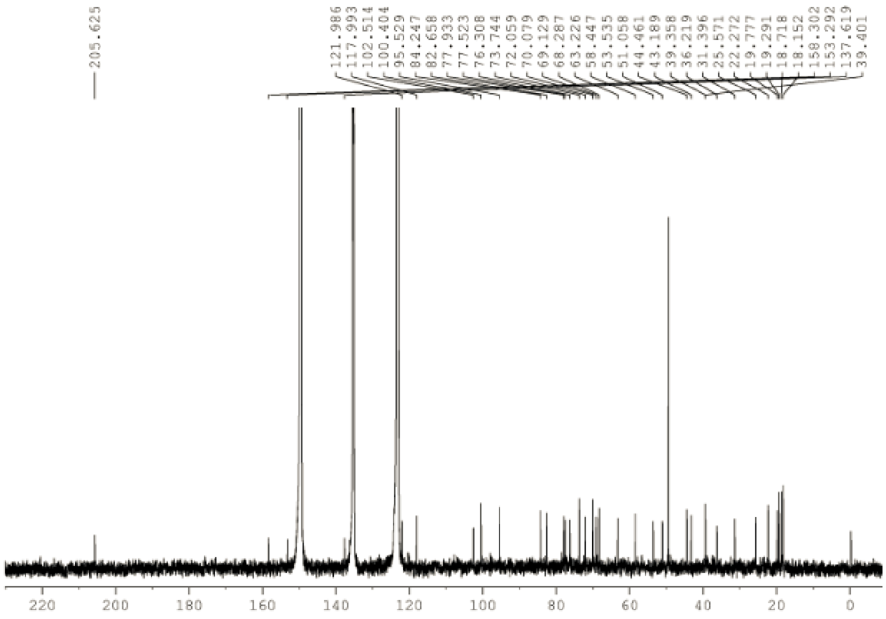

Figure S61. The HSQC spectrum of compound **11**

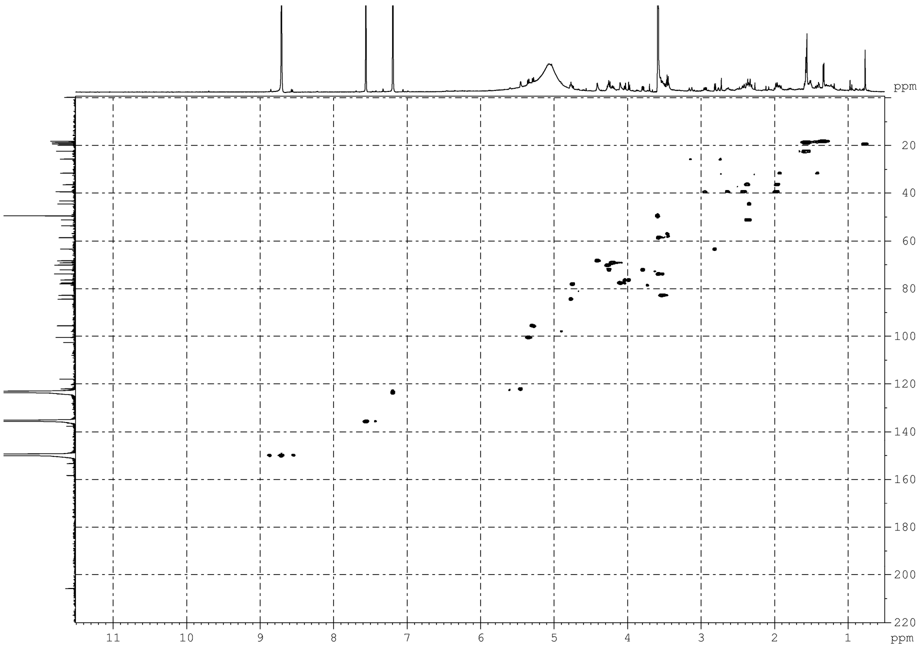

Figure S62. The HMBC spectrum of compound **11**

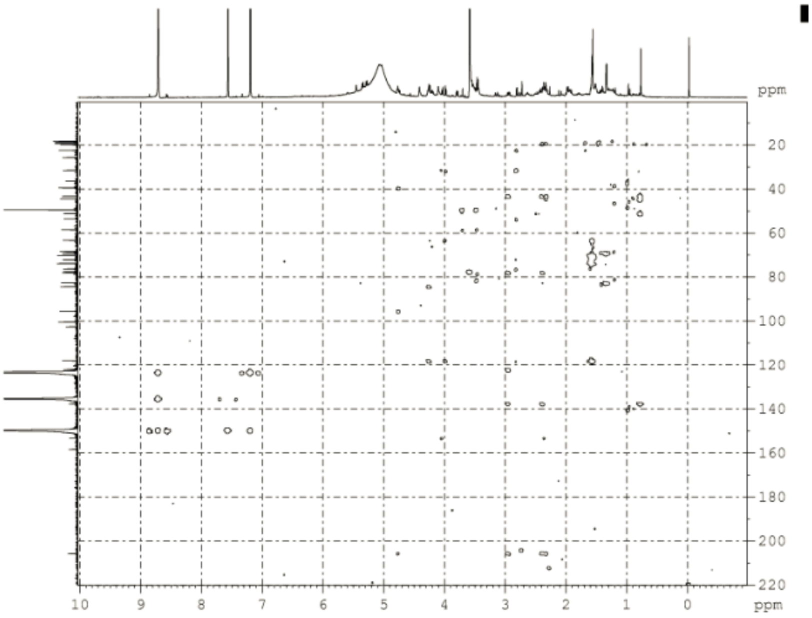

Figure S63. The NOESY spectrum of compound **11**

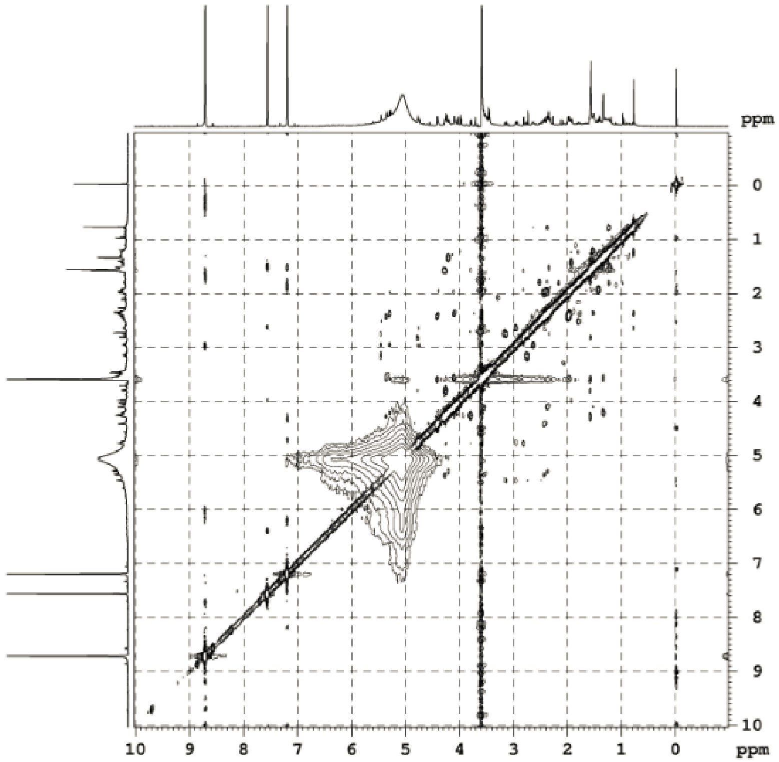

10. The spectra of compound 12

Figure S64. The IR spectrum of compound **12**

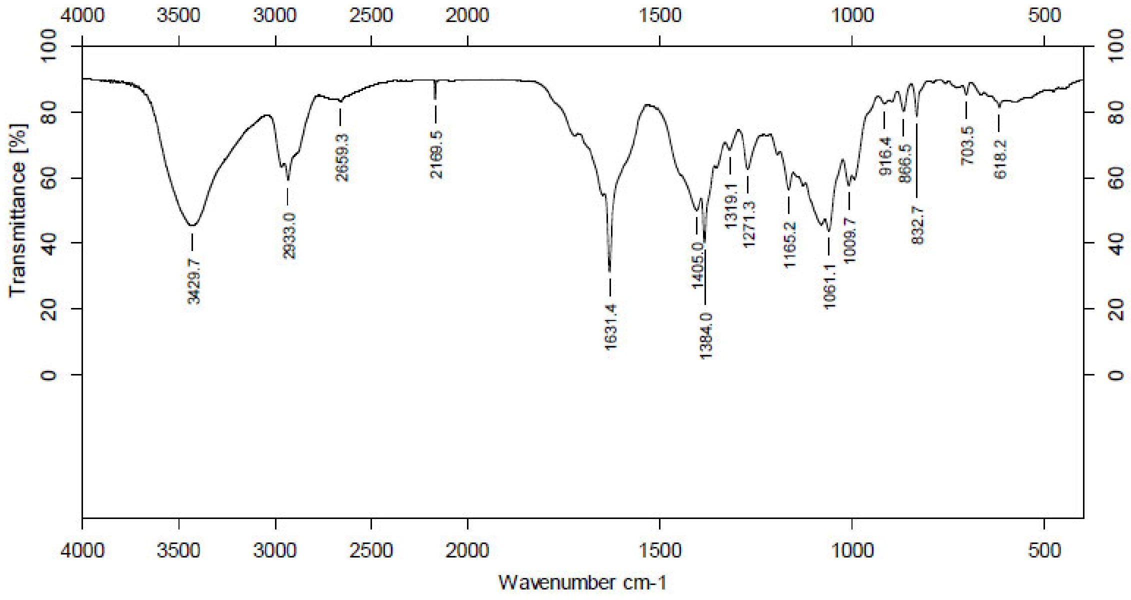

Figure S65. The HR-ESI-MS spectrum of compound **12**

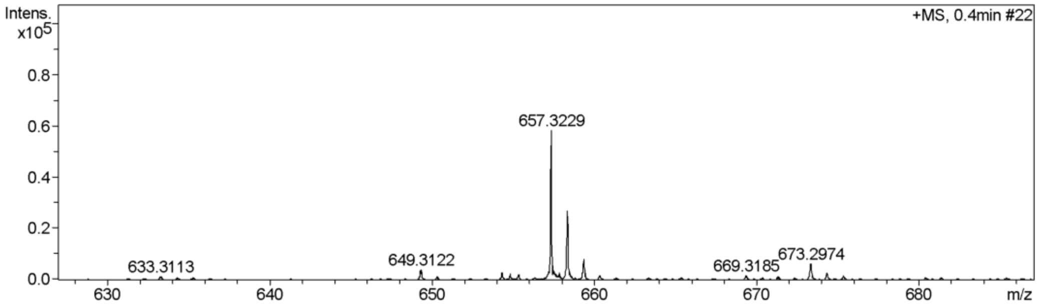

Meas. m/z	#	Formula	m/z	err [ppm]	Mean err [ppm]	rdb	N-Rule	e⁻ Conf	mSigma	Std I	Std Mean m/z	Std I VarNorm	Std m/z Diff	Std Comb Dev
657.3229														
	1	C 34 H 50 Na O 11	657.3251	3.4	5.5	9.5	ok	even	49.28	0.0719	0.0043	0.0239	0.0045	0.8427

Figure S66. The ^{1}H-NMR spectrum of compound **12**

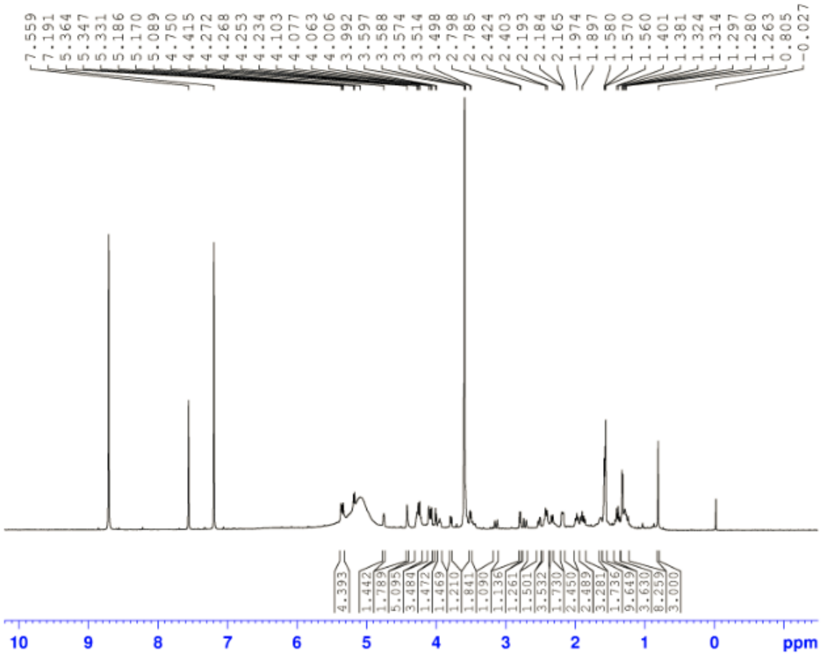

Figure S67. The ^{13}C-NMR spectrum of compound **12**

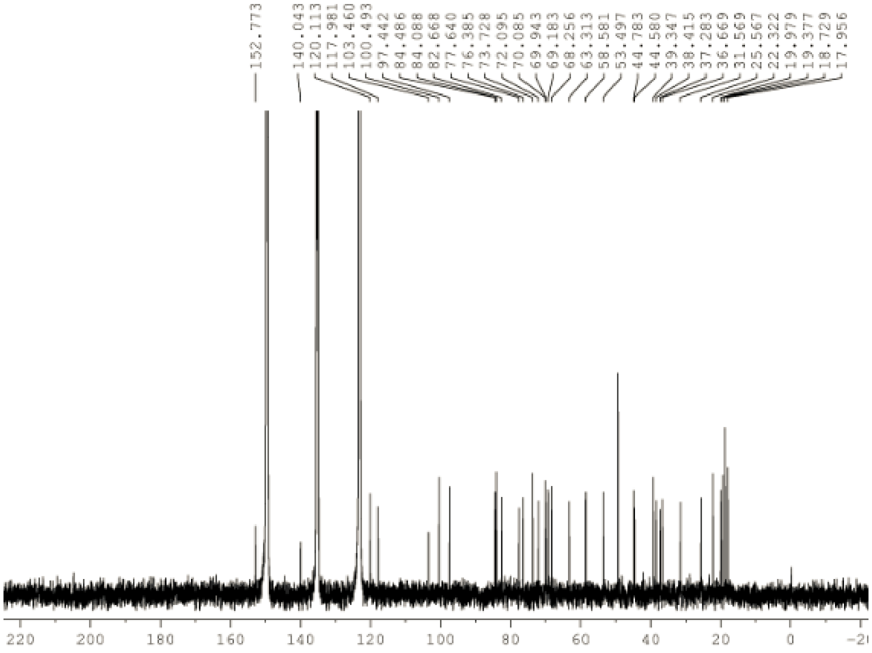

Figure S68. The HSQC spectrum of compound **12**

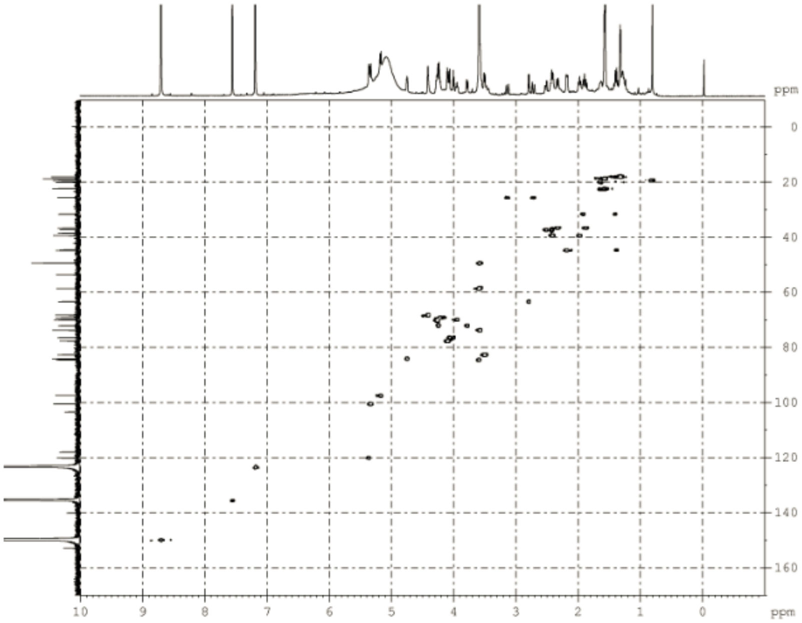

Figure S69. The HMBC spectrum of compound **12**

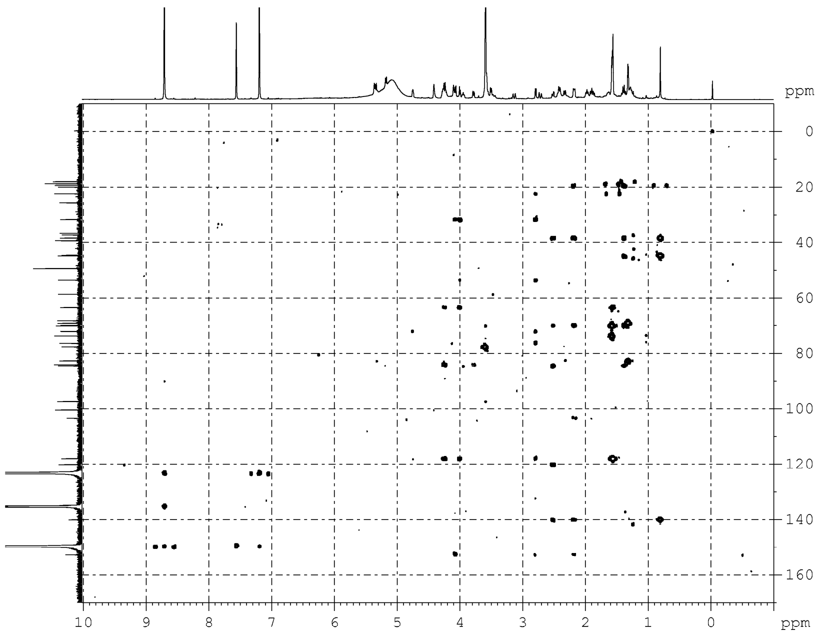

Figure S70. The NOESY spectrum of compound **12**

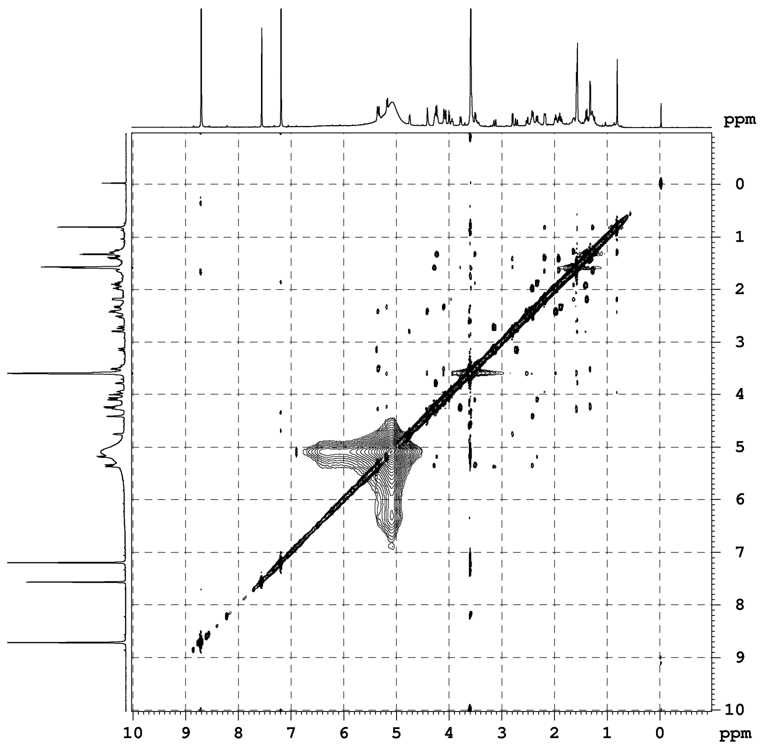

11. The spectra of compound 13

Figure S71. The IR spectrum of compound **13**

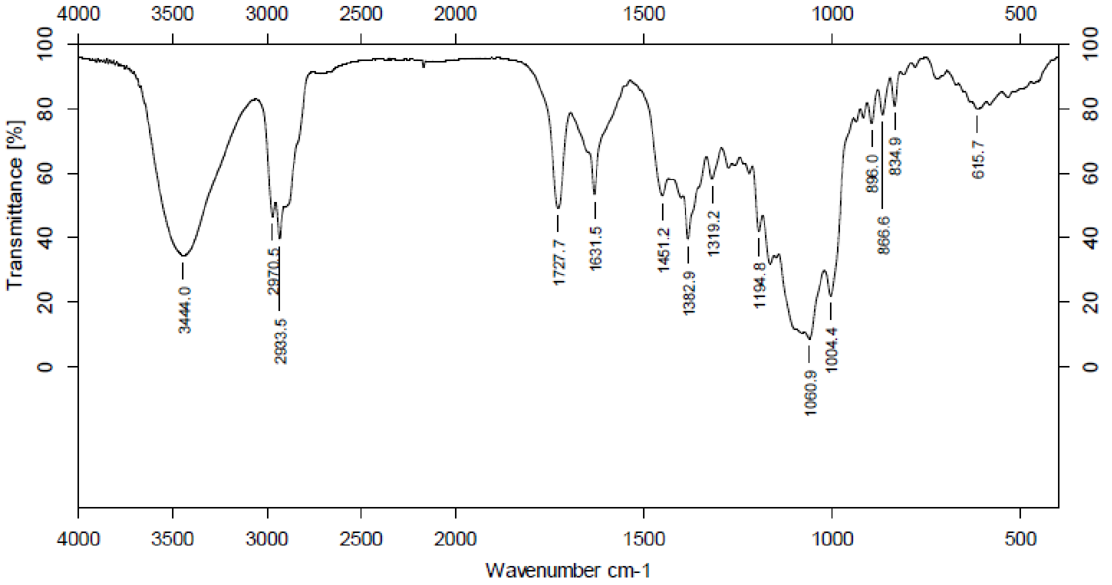

Figure S72. The HR-ESI-MS spectrum of compound **13**

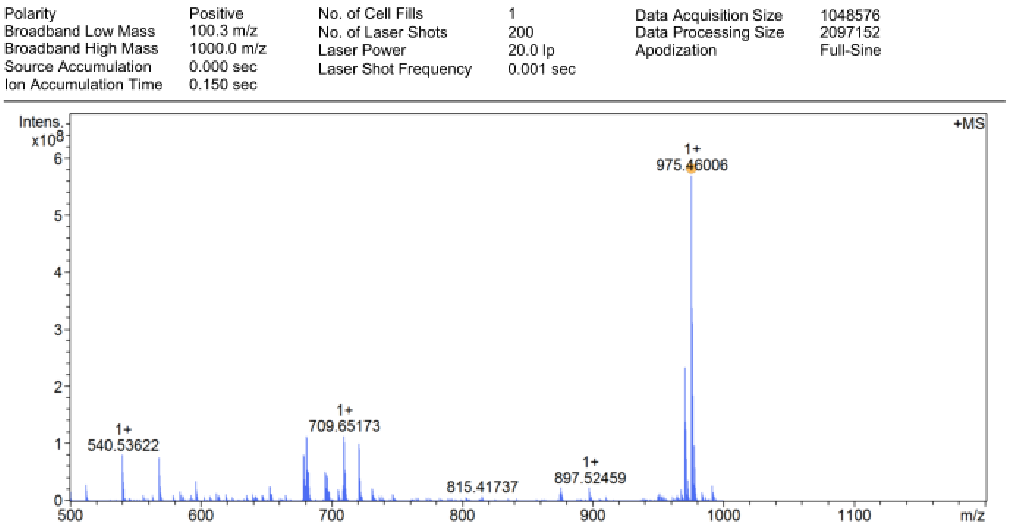

Meas. m/z	#	Ion Formula	Score	m/z	err [ppm]
975.460060	1	C48H72NaO19	16.89	975.456001	-4.2

Figure S73. The ^{1}H-NMR spectrum of compound **13**

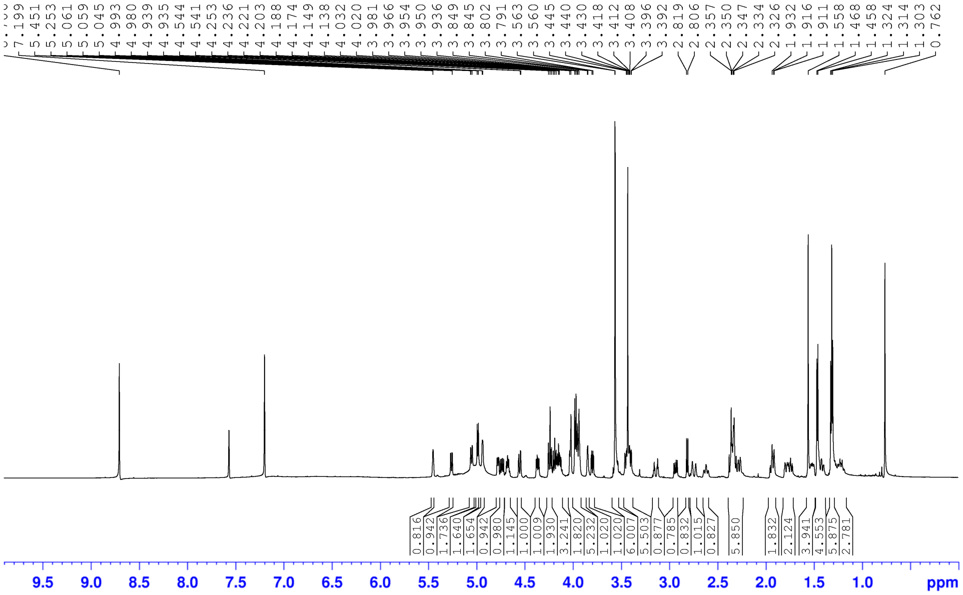

Figure S74. The ^{13}C-NMR spectrum of compound **13**

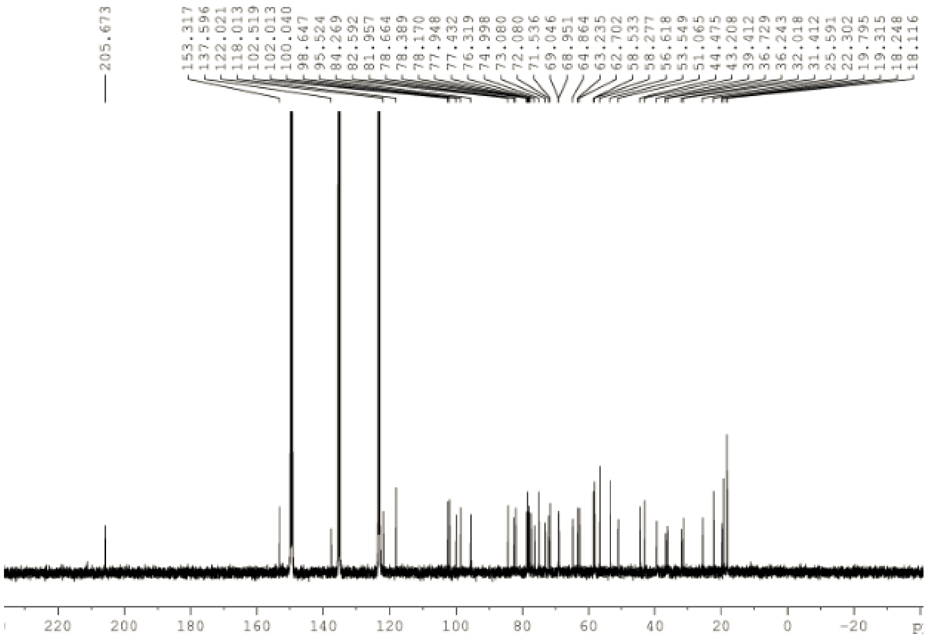

Figure S75. The HSQC spectrum of compound **13**

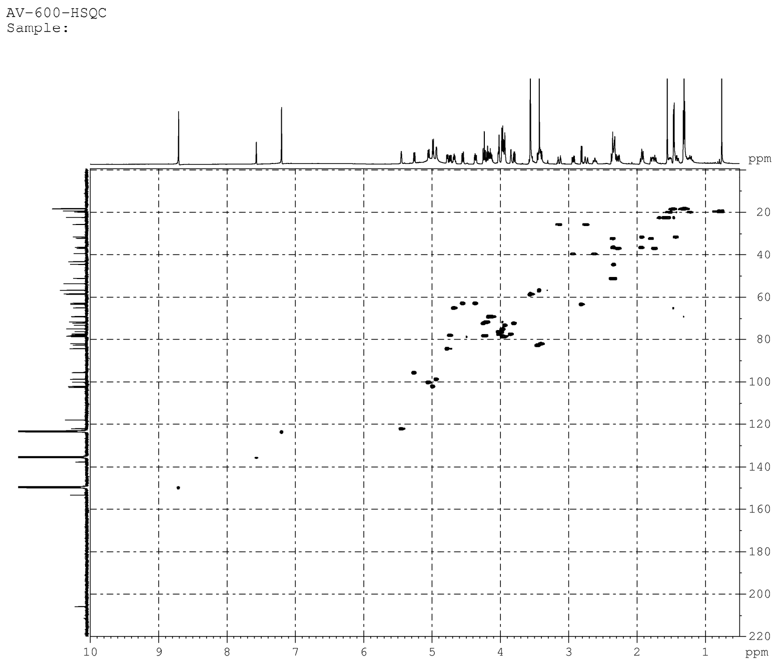

Figure S76. The HMBC spectrum of compound **13**

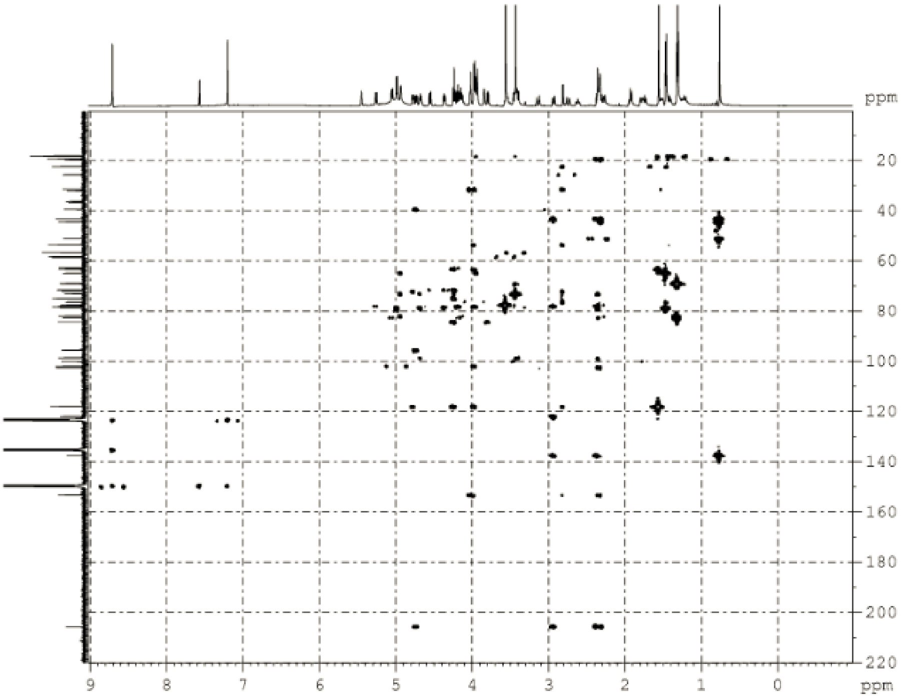

Figure S77. The NOESY spectrum of compound **13**

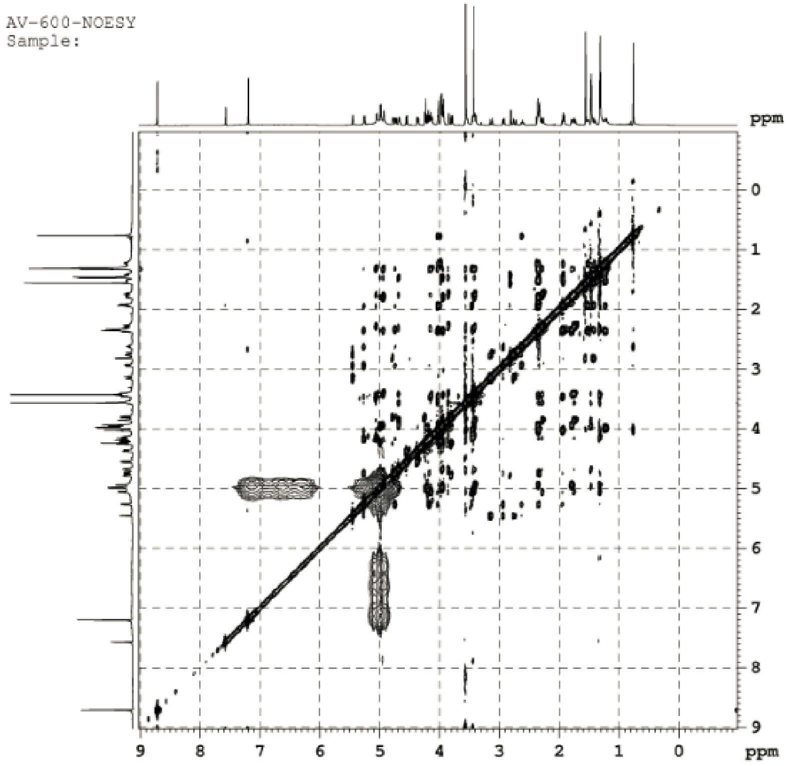

12. The spectra of compound 14

Figure S78. The IR spectrum of compound **14**

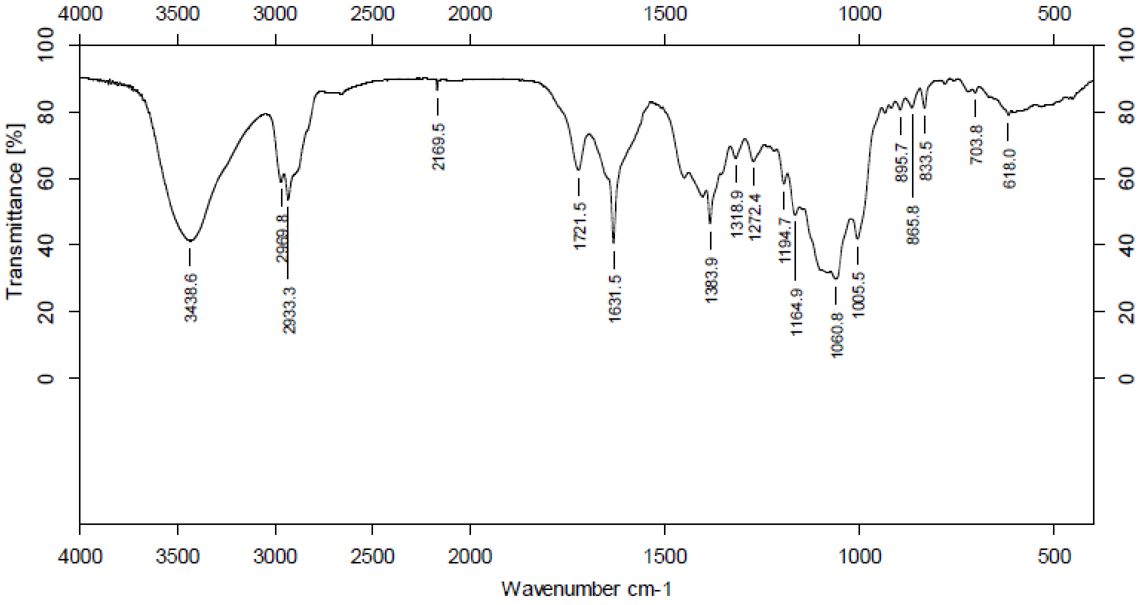

Figure S79. The HR-ESI-MS spectrum of compound **14**

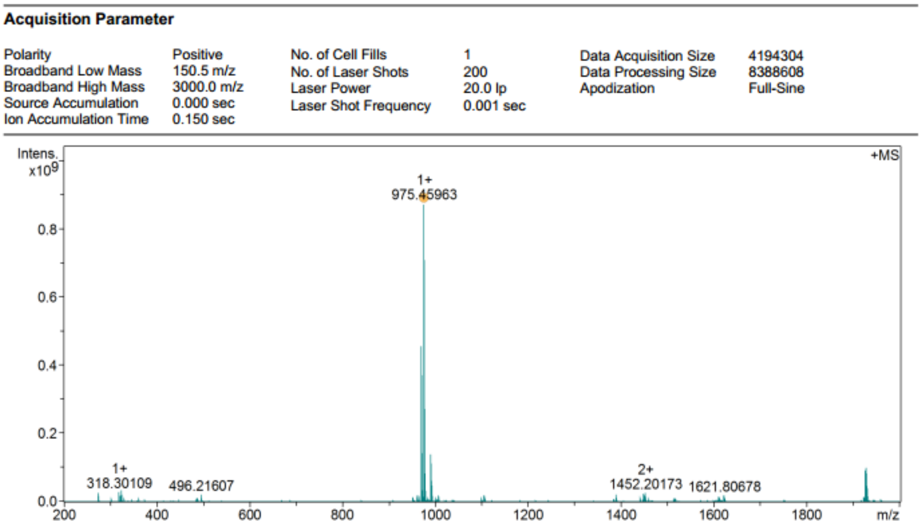

Meas. m/z	#	Ion Formula	Score	m/z	err [ppm]
975.459633	1	C48H72NaO19	24.55	975.456001	-3.7

Figure S80. The ^{1}H-NMR spectrum of compound **14**

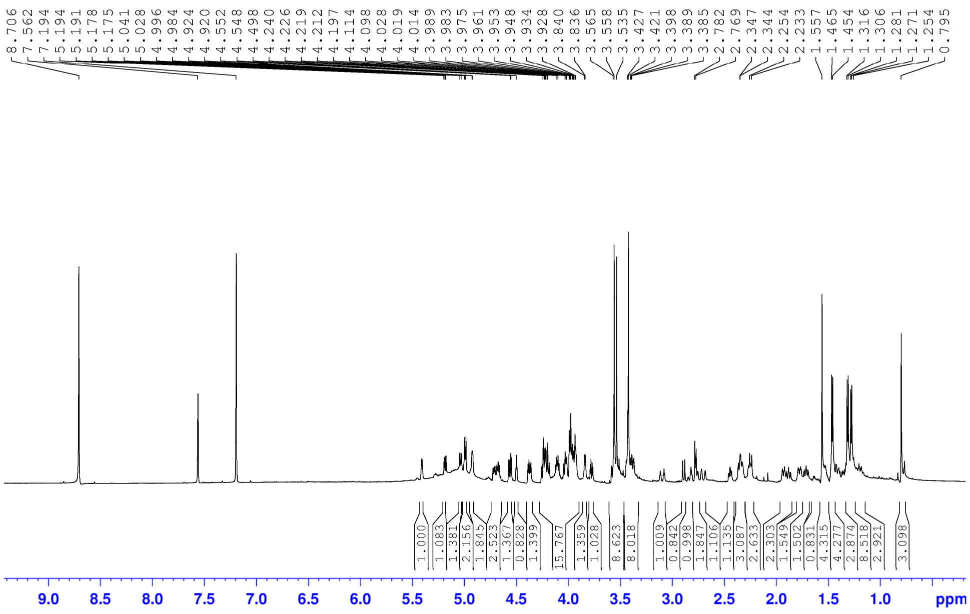

Figure S81. The ^{13}C-NMR spectrum of compound **14**

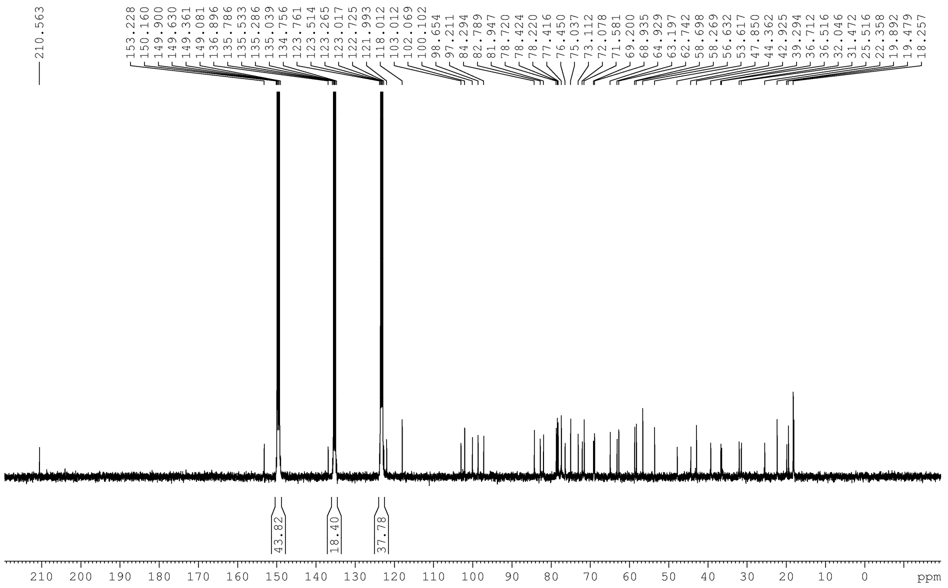

Figure S82. The HSQC spectrum of compound **14**

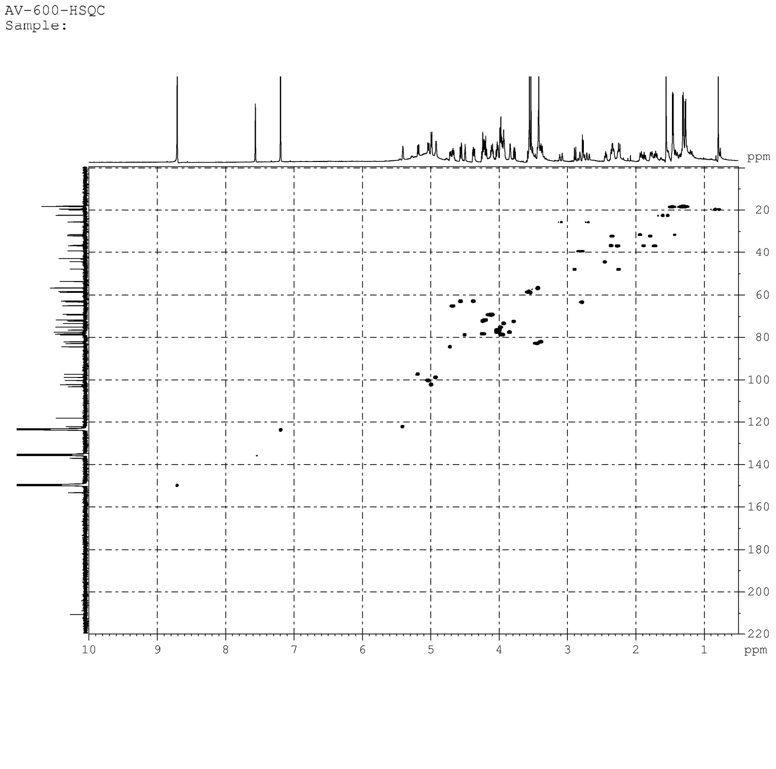

Figure S83. The HMBC spectrum of compound **14**

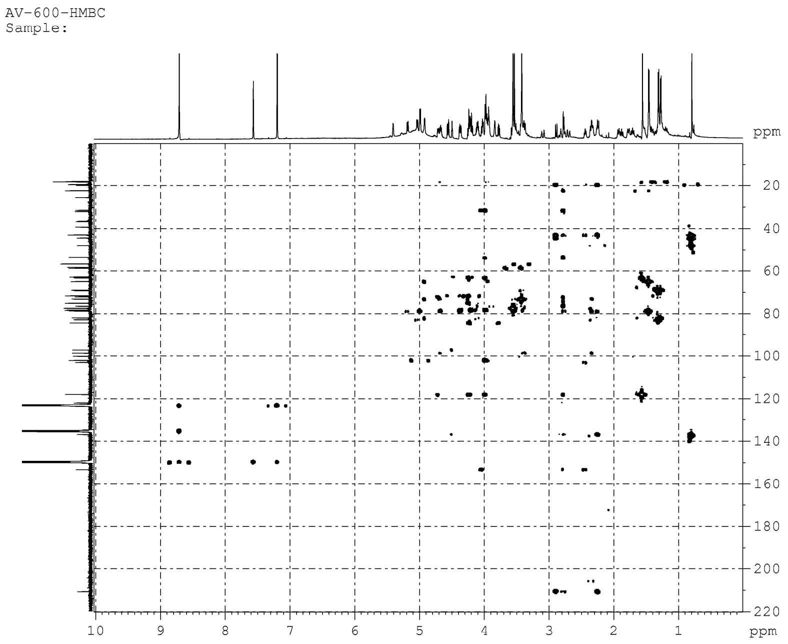

Figure S84. The NOESY spectrum of compound **14**

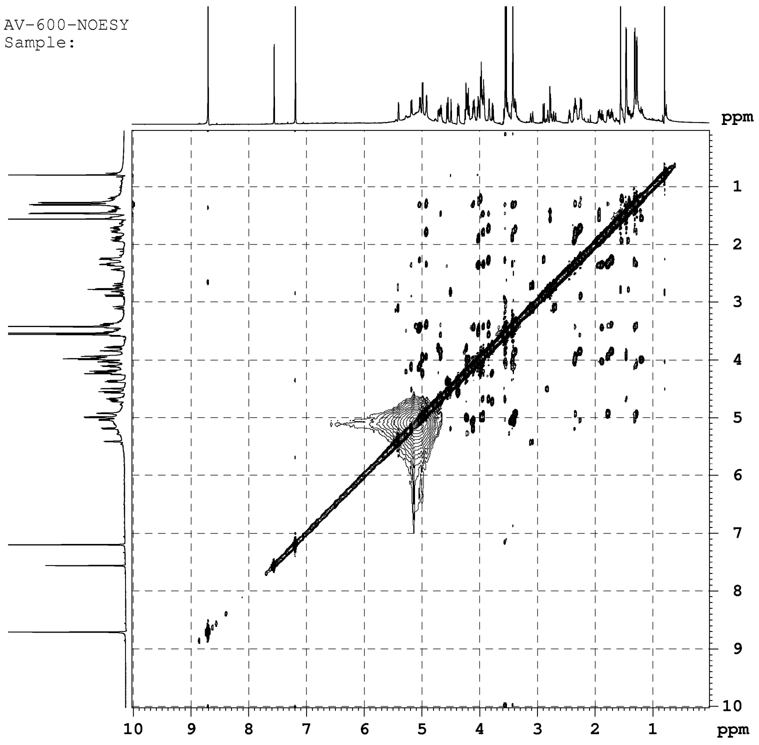

13. The spectra of compound 5

Figure S85. The ^{1}H-NMR spectrum of compound **5**

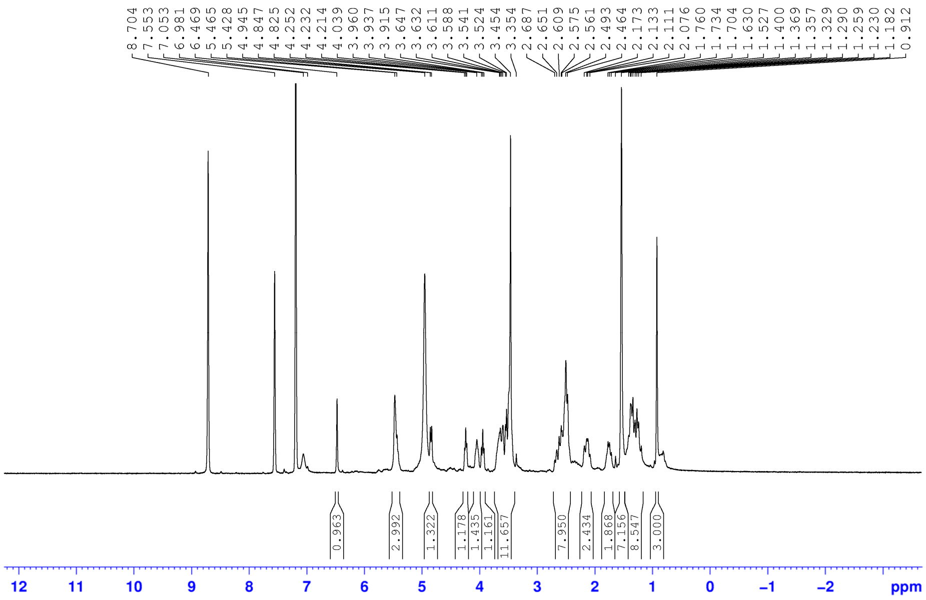

Figure S86. The ^{13}C-NMR spectrum of compound **5**

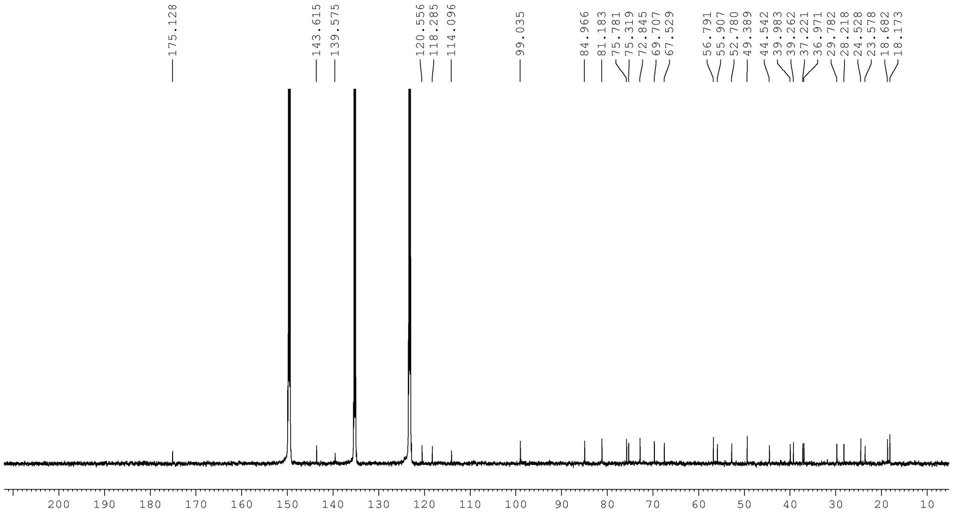

14. The spectra of compound 6

Figure S87. The ^{1}H-NMR spectrum of compound **6**

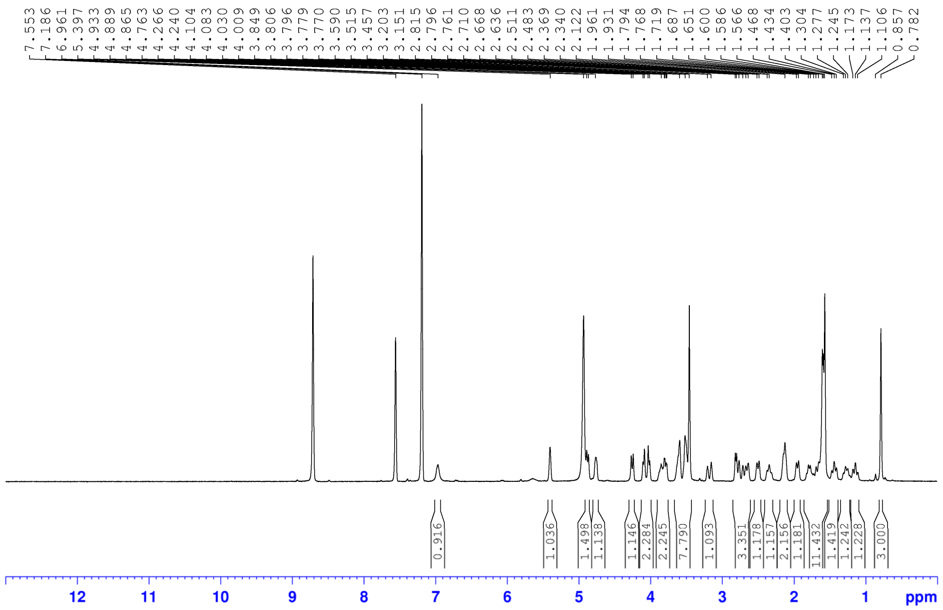

Figure S88. The ^{13}C-NMR spectrum of compound **6**

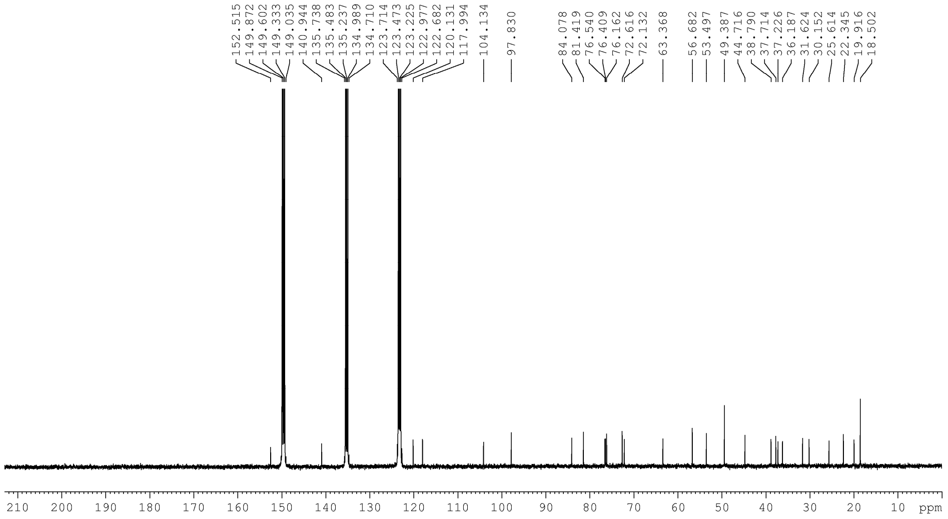

Supplement: Supplementary file 1 [file molecules-27-05500-s001.zip › molecules-1849660-supplementary.pdf]
